# Novel Thiazolidine-2,4-dione-trimethoxybenzene-thiazole Hybrids as Human Topoisomerases Inhibitors

**DOI:** 10.3390/ph16070946

**Published:** 2023-06-29

**Authors:** Maria Stefania Sinicropi, Jessica Ceramella, Patrice Vanelle, Domenico Iacopetta, Camillo Rosano, Omar Khoumeri, Shawkat Abdelmohsen, Wafaa Abdelhady, Hussein El-Kashef

**Affiliations:** 1 Department of Pharmacy, Health and Nutritional Sciences, University of Calabria, 87036 Arcavacata di Rende, Italy; s.sinicropi@unical.it (M.S.S.); jessica.ceramella@unical.it (J.C.); 2 Aix Marseille University, CNRS, ICR UMR 7273, Equipe Pharmaco-Chimie Radicalaire, Faculté de Pharmacie, 27 Boulevard Jean Moulin, CS30064, CEDEX 05, 13385 Marseille, France; patrice.vanelle@univ-amu.fr (P.V.); omar.khoumeri@univ-amu.fr (O.K.); 3 U.O. Proteomica e Spettrometria di Massa, IRCCS Ospedale Policlinico San Martino, Largo R. Benzi 10, 16132 Genova, Italy; camillo.rosano@hsanmartino.it; 4 Department of Chemistry, Faculty of Science, Assiut University, Assiut 71516, Egypt; shawk662001@yahoo.com (S.A.); wafaahadi13@gmail.com (W.A.); 5 Faculty of Pharmacy, Sphinx University, New Assiut 71684, Egypt

**Keywords:** hybrid drug design, trimethoxybenzene, thiazolidinedione, thiazole, anticancer activity, human topoisomerases inhibition

## Abstract

Cancer is a complex and heterogeneous disease and is still one of the leading causes of morbidity and mortality worldwide, mostly as the population ages. Despite the encouraging advances made over the years in chemotherapy, the development of new compounds for cancer treatments is an urgent priority. In recent years, the design and chemical synthesis of several innovative hybrid molecules, which bring different pharmacophores on the same scaffold, have attracted the interest of many researchers. Following this strategy, we designed and synthetized a series of new hybrid compounds that contain three pharmacophores, namely trimethoxybenzene, thiazolidinedione and thiazole, and tested their anticancer properties on two breast cancer (MCF-7 and MDA-MB-231) cell lines and one melanoma (A2058) cell line. The most active compounds were particularly effective against the MCF-7 cells and did not affect the viability of the normal MCF-10A cells. Docking simulations indicated the human Topoisomerases I and II (hTopos I and II) as possible targets of these compounds, the inhibitory activity of which was demonstrated by the mean of direct enzymatic assays. Particularly, compound **7e** was proved to inhibit both the hTopo I and II, whereas compounds **7c,d** blocked only the hTopo II. Finally, compound **7e** was responsible for MCF-7 cell death by apoptosis. The reported results are promising for the further design and synthesis of other analogues potentially active as anticancer tools.

## 1. Introduction

Cancer remains one of the foremost and crucial public health concerns. It is identified as the second leading cause of death after cardiovascular pathologies worldwide [1,2], where about 19.3 million new cancer events and about 10.0 million cancer deaths were recorded in the year 2020 [3]. Among women, breast cancer (BC) is the most frequently diagnosed and prevalent cancer, and has been reported as the second leading cause of death after lung cancer [4]. The World Health Organization estimated 2.3 million cases of breast cancer, with 685,000 deaths around the world, in 2020 [5]. Currently, chemotherapy is the main treatment for BC, but its toxicity to normal cells and acquired drug resistance are the major drawbacks. Thus, the development of safer and more potent anti-BC agents is highly required [6]. Thiazolidine-2,4-diones (TZDs) were proved to be privileged pharmacophore scaffolds in medicinal chemistry [7]. They possess a wide range of pharmacological properties [8,9,10,11,12,13], including anticancer activity [14]. It was described that TZDs may affect cancer onset, progression and metastasis [15,16]. These effects are due to the interference with the Raf/MEK/extracellular signal regulated kinase (ERK) [17], phosphatidylinositol 3-kinase (PI3K)/AKT [18], Wnt signal transduction pathways [19], PIM-1 and PIM-2 protein kinases overexpression [20], androgen receptor regulated genes [21,22] and peroxisome proliferator-activated receptors (PPARs) signaling cascades [23], which are often up-regulated in human cancers. Moreover, thiazole derivatives are known by their anticancer activities [24], in addition to their variety of other biological properties [25,26,27,28,29,30,31,32,33,34,35,36,37]. It is of interest to note that some thiazole-containing anticancer drugs are already available on the market, such as tiazofurin [38], dasatinib [39], and dabrafenib [40]. Considerable improvements in the biological activity were achieved by using the hybrid bioactive molecule approach, which combines two or more different pharmacophore moieties in a new molecule [41]. Thus, the combination of TZD and thiazole scaffolds in a single hybrid molecule may exhibit synergic anticancer effects [42]. We have previously synthesized a series of thiazolidine-2,4-dione derivatives as anti-breast cancer compounds [43] that were tested against MCF-7 and MDA-MB-231 cancer cell lines, as well as against normal breast cells, isolated from the same subjects. Three derivatives were found to be the most active, and they inhibited the breast cancer cells’ proliferation in a dose-dependent fashion. Encouraged by these results, and taking into consideration the hybrid bioactive molecule approach, we present herein the synthesis of new thiazolidinedione scaffolds. The latter contain the three pharmacophores, namely trimethoxybenzene, thiazolidinedione and thiazole, in a single molecule. In this way, we aimed to obtain hybrid compounds with high potential as anti-breast cancer tools. Interestingly, different compounds containing the above-mentioned pharmacophores were investigated as topoisomerase inhibitors [44,45,46]. Topoisomerases are enzymes possessing nuclease and ligase activities and are involved in many important cellular processes, such as DNA transcription and duplication, chromosome segregation and chromatin assembly [47]. There are two types of DNA topoisomerases, I and II, based on their amino acid sequences and structures, catalytic functions and reaction mechanisms. Both are implicated in tumor onset and progression, being often overexpressed in many cancer cells [48]. Thus, by the means of in silico simulations and direct enzymatic assays, we found that our compounds inhibited the human topoisomerase I (hTopo I) and II (hTopo II). Particularly, compound **7e** was able to block both the hTopo I and II activities, whereas compounds **7c** and **7d** were found to block only hTopo II activity. Finally, MCF-7 cells exposed to compound **7e**, which was the most active of the series and induced cancer cell death by activating the intrinsic apoptotic mechanism.

## 2. Results

### 2.1. Chemistry

**Scheme 1:**
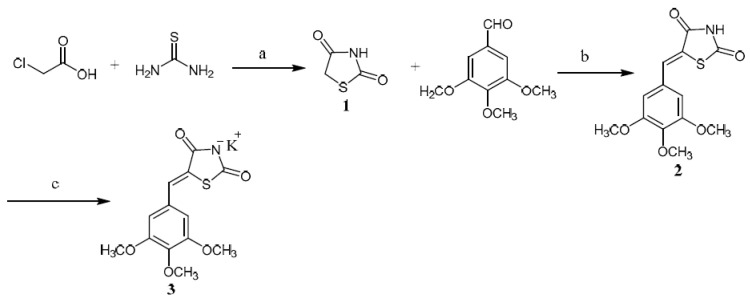
Synthetic route for preparation of the potassium salt **3**. Reagents and conditions: (a) i. water, 0–5 °C, 20 min, ii. HCl, reflux, 12 h; (b) anhydrous toluene, catalytic amount of piperidinium acetate, reflux, 15 h; (c) KOH/ethanol, 80 °C, 20 min.

The aim of this present work was to prepare a new series of thiazolidine-2,4-diones and evaluate their anticancer activity. Our target molecules were designed by combining three pharmacophores known for their anticancer activity, namely thiazolidine-2,4-dione, trimethoxyphenyl, and thiazole moieties, in a single molecule. Thus the thiazolidine-2,4-dione pharmacophore nucleus (**1**) was prepared by the reaction of chloroacetic acid with thiourea, as reported in our earlier paper [43]. The active methylene group of **1** was allowed to condense with 3,4,5-trimethoxybenzaldehyde via Knoevenagel reaction in boiling anhydrous toluene, in the presence of a catalytic amount of piperidinium acetate, using a Dean–Stark apparatus to produce the corresponding (*Z*)-5-(3,4,5-trimethoxybenzylidine)thiazolidine-2,4-dione (**2**). The *Z*-conformation of the latter compound has been proven by x-ray analysis [49]. Treatment of compound **2** with ethanolic KOH solution gave the corresponding potassium salt **3** (Scheme 1). 

**Scheme 2:**
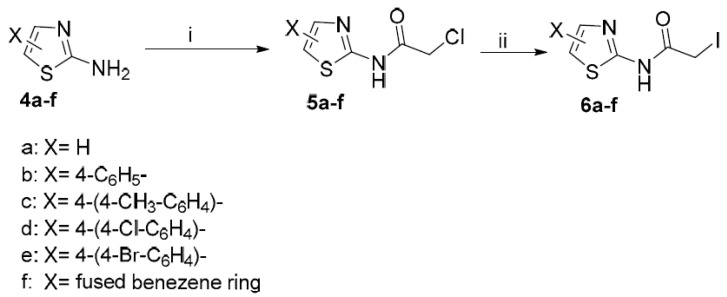
Synthesis of 2-chloro- and 2-iodoacetamides **5a**-**f** and **6a**-**f** respectively. Reagents and conditions: (i) 1. ClCH_2_COCl, anhydrous benzene, boiling water bath, overnight, 2. NaHCO_3_ solution, (ii) KI, acetone, reflux, 4 h.

A thiazole moiety, as a third pharmacophore, was incorporated in the (3,4,5-trimethoxybenzylidene)thiazolidine-2,4-dione system. Thus, a series of 2-aminothiazoles, **4a–f,** were chloroacetylated using 2-chloroacetylchloride in dry benzene to give the 2-chloroacetamides **5a**–**f** (Scheme 2). It is to be noted that **4b**–**e** were prepared using the method reported by Dodson and King [50]. The reaction of 2-chloroacetamides **5a**–**f** with the potassium salt **3,** in dry DMF, gave the target compounds **7a**–**f**, however, in rather low yields (50–54%). In an endeavor to increase the reaction yield, the chlorine atom of **5a**–**f** was displaced by the more labile iodine atom upon treatment with KI in boiling acetone to give the corresponding 2-iodoacetamides **6a**–**f** (Scheme 2).

**Scheme 3:**
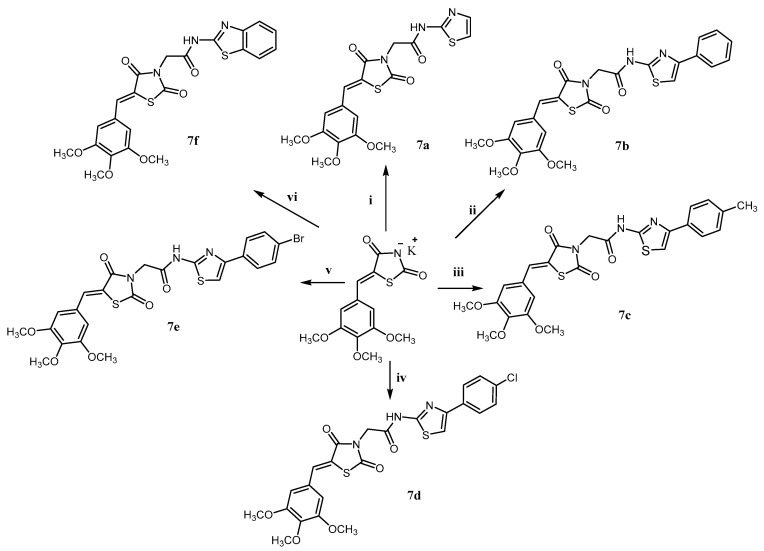
Synthesis of the target hybrid molecules. Reagents and conditions:(i) **6a**, DMF, boiling water bath, overnight; (ii) **6b**, DMF, boiling water bath, overnight; (iii) **6c**, DMF, boiling water bath, overnight; (iv) **6d**, DMF, boiling water bath, overnight; (v) **6e**, DMF, boiling water bath, overnight; **6f**, DMF, boiling water bath, overnight.

The reaction of the latter iodo derivatives **6a–f** with the potassium salt **3** gave **7a**–**f** in excellent yields (69–97%) (Scheme 3).

The structure of compounds **7a**–**f** was confirmed by IR, ^1^H and ^13^C NMR spectral analyses. The IR spectra were characterized by a stretching vibration band (ν NH) at 3423–3184 cm^−1^ in addition to ν C=O bands at 1690–1685 and 1743–1736 cm^−1^. The ^1^H-NMR spectra of the basic skeleton showed, in addition to other protons, characteristic signals of the nine protons of the three methoxyl groups (in addition to those of the other substituents at the thiazole N atom). In DMSO_d6_, these nine protons appeared as two singlets at δ 3.77–3.76 and 3.87–3.86 ppm, integrated for three protons (of the 4-methoxy substituent) and six protons (of the 3,5-dimethoxy substituents), respectively. The proton of the methylidene group appeared as a singlet at δ 7.94–7.58 ppm. The two aromatic protons of the trimethoxyphenyl group appeared as a singlet at δ 7.0–6.98 ppm. The N-CH_2_-CO methylene protons appeared as a singlet at δ 4.69–4.65 ppm. The -NHCO- proton appeared at δ 12.76–12.58 ppm. The ^13^C NMR spectra were consistent with the structures of all of the compounds obtained. 

### 2.2. Docking Studies

**Figure 1:**
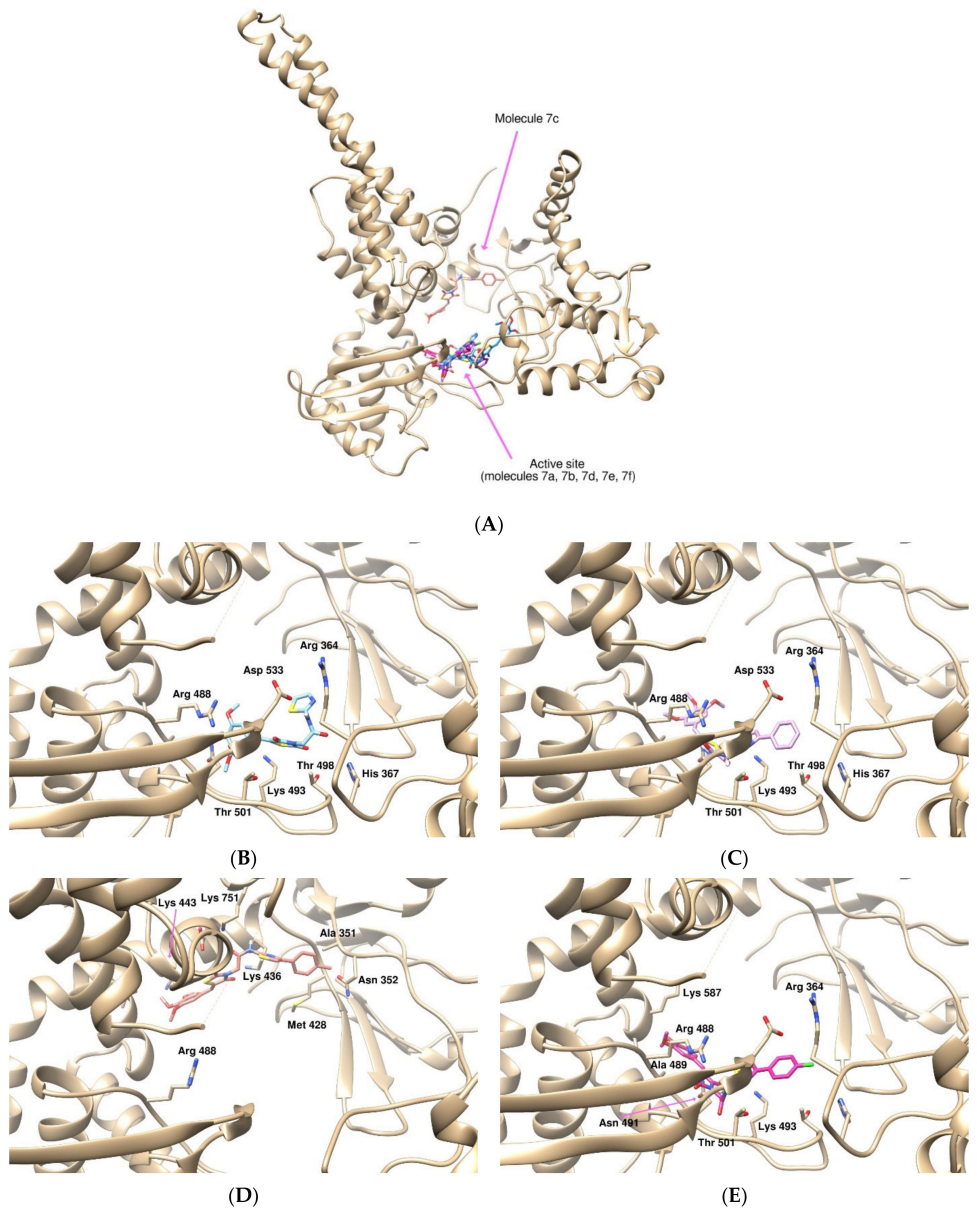

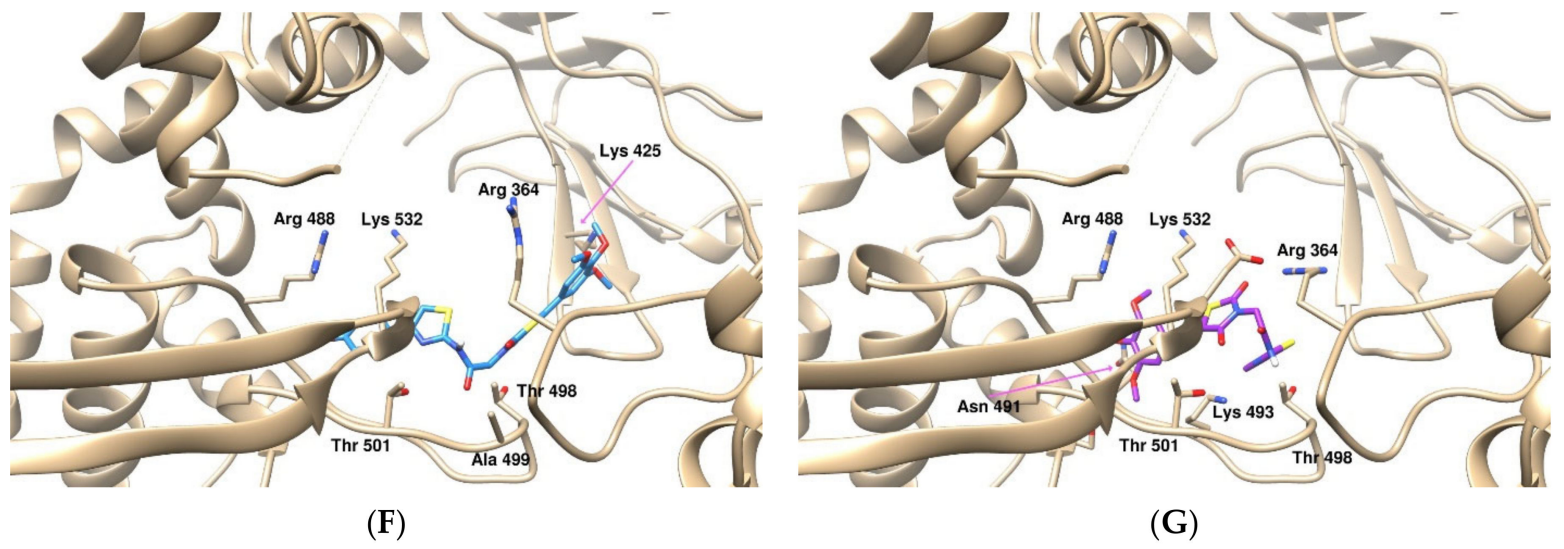
Panel (**A**) The three-dimensional structure of a monomeric hTopo I is drawn as a tanned ribbon. The active site and the binding site for molecule **7c** are highlighted and indicated by arrows. Close-up visualizations of the active site show the binding modes of molecule **7a** (Panel (**B**)), **7b** (Panel (**C**)), **7c** (Panel (**D**)), **7d** (Panel (**E**)), **7e** (Panel (**F**)) and **7f** (Panel (**G**)). The principal amino acids interacting with the different compounds are evidenced as sticks and properly labeled.

To visualize the poses and the possible binding modes among the new compounds and the protein targets (hTopo I and hTopo II), we decided to perform molecular docking simulations. As these molecules had never been investigated before in vitro, for our simulations, we adopted a “blind-docking approach”: no *a priori* information on the binding site was given to the system. This procedure had already been successfully used for other studies [51]. This approach is useful as it allows us, on the one hand, to identify the best candidates among the compounds and, on the other, to improve the atomic structure of our molecules for a better design and synthesis of improved lead compounds. We based our investigation on the compounds’ binding affinity for the two hTopos, using the Autodock software that calculates a binding affinity constant (Ki) on the basis of the binding energy, according to the Ki = exp (deltaG/(R × T) expression. The clustering of the results from the simulations was taken into account for discriminating the most effective candidates, as previously reported [52]. Finally, the binding mode was visually examined for the evaluation of protein–ligand interaction quality. We showed that our compounds can bind the hTopo I and II, creating different hydrogen and hydrophobic interactions. Concerning hTopo I, our molecules are placed in three distinct areas belonging to the region where DNA binds. Most of our compounds (specifically molecules **7a**, **7b**, **7d**, **7e**, **7f**) occupy the whole protein active site region. These molecules are almost superposed, in a region (Figure 1 panel A) nested between two protein subdomains. Molecule **7a** forms hydrogen bonds with residues Arg 364, Ser 423, Lys 463 and Thr 501, and **7b** is bound via residues Arg 488, Lys 493, Thr 501 and Lys 532. The chlorine moiety of molecule **7d** is involved in the formation of a halogen bond with Ser 423. Furthermore, this compound interacts with Arg 488, Lys 493, Thr 501, Lys 532 and Lys 590. Residues Arg 364, Ser 423 and Lys 493 form hydrogen bonds with compound **7e**, while Arg 488 and Trp 416 further stabilize this compound by π–π stacking interactions. Finally, molecule **7f** forms hydrogen bonds with Arg 364, Arg 488, Lys 493, Thr 501 and Arg 532. On the other hand, compound **7c** is located on a distinct site in the N-terminal domain, in proximity of active site residues His 468 and Arg 590 on the opposite part of the channel hosting the DNA. Although this molecule does not occupy the whole active site area, it can still disturb the regular functions of hTopo I by interfering with the process of protein: DNA recognition and binding through the formation of hydrogen bonds with residues Lys 216, Glu 438 and Arg 590 (Figure 1 panels B–G). Two-dimensional plots for hTopo I were calculated using LIGPLOT [53] and included in the Supplementary Materials (Figures S59–S64). 

**Figure 2:**
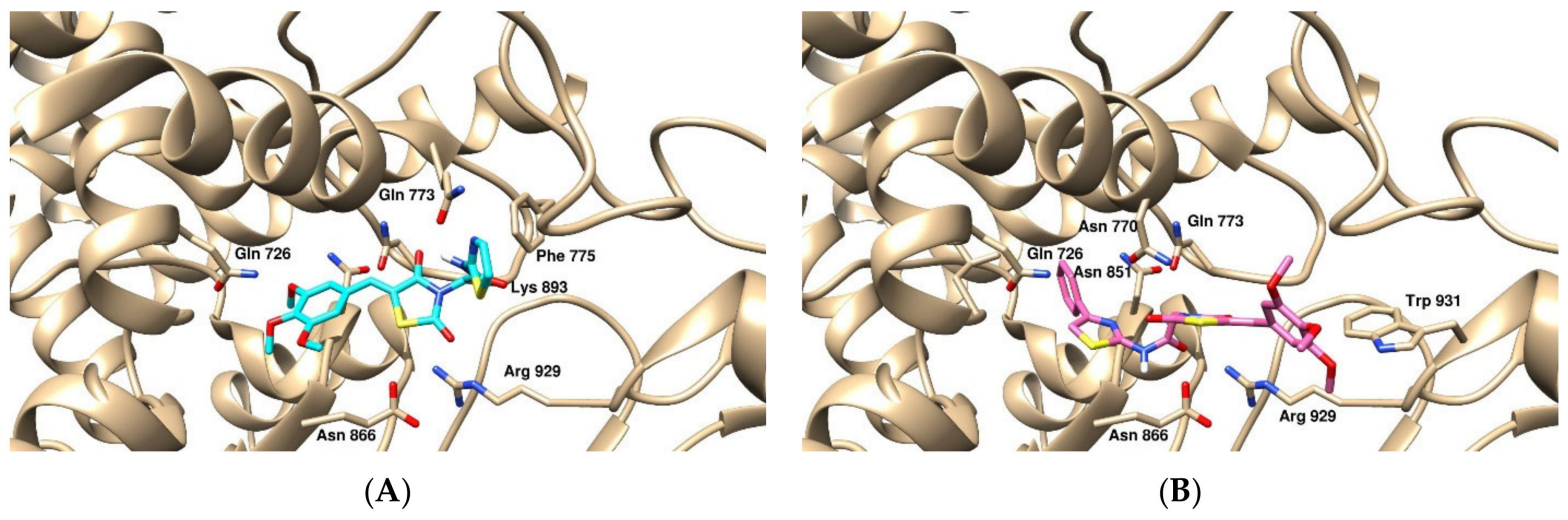

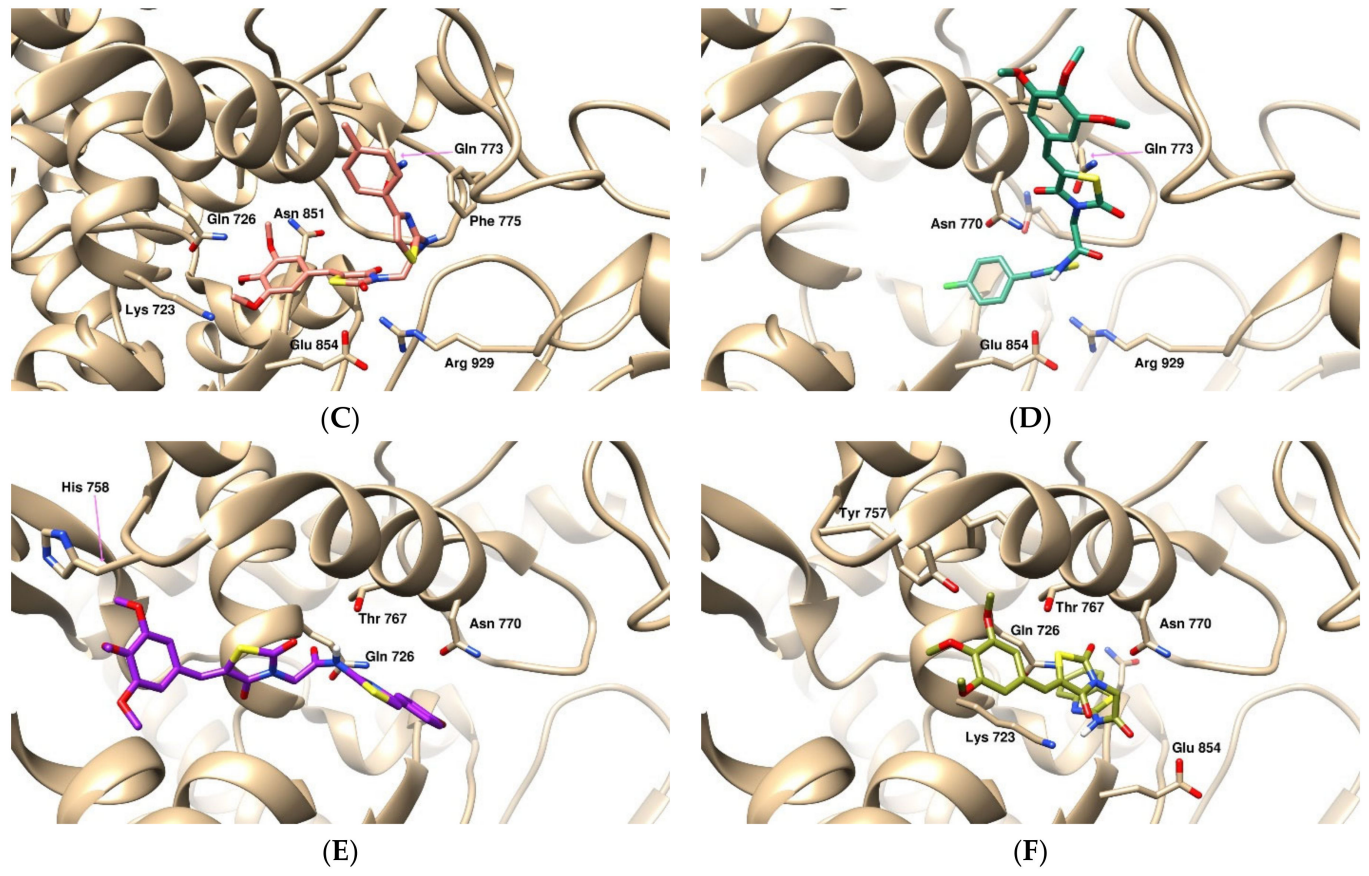
Panel (**A**) A ribbon representation of hTopo II binding cleft in complex with compound **7a**. Panel (**B**) reports the binding mode of compound **7b** to hTopo II. Panel (**C**) illustrates the pose of compound **7c.** Panel (**D**) shows how compound **7d** adopts a position that is head-to-tail rotated with respect to molecule **7c.** Panel (**E**) illustrates the interactions of compound **7e** with hTopo II. Panel (**F**) reports the pose of compound **7f**. In all the figures, we adopted the same orientation of the protein.

The above-described molecules also bind the hTopo II near the DNA gate. Residues Gln 726, Asn 770, Asn 851, Asn 866, and Lys 893 are hydrogen-bonded to molecule **7a**, while Phe 775 forms van der Waals contact. The same residues also form hydrogen bonds with compound **7b**, which is stabilized by a π–π stacking with Trp 931. Molecule **7c** forms hydrogen bonds with Asn 866 and Lys 893, and it is stabilized by hydrophobic interactions with residues Ile 769, Phe 755 and Leu 1178. Residue Lys 723 forms a halogen bond with the Cl moiety of molecule **7d**, which also forms hydrogen bonds with Asn 866, Asn 770 and Lys 893. Furthermore, this compound is stabilized by hydrophobic interactions with residues Ile 769, Phe 755 and Leu 1178. Compounds **7e** and **7f** are positioned in a slightly different place. They both bind residues Ser 763, Asn 766 and Gln 726, but the longer chain of molecule **7e** allows this compound to also interact with residues Lys 723 and His 759 (Figure 2 panels A–F). Two-dimensional plots for hTopo II were calculated using LIGPLOT [53] and included in the Supplementary Material (Figures S65–S70).

### 2.3. Biology

#### 2.3.1. Effects on Cell Viability

All of the synthesized compounds (**7a**–**f**) were studied to test their anticancer activity toward the estrogen receptor-positive (ER+) MCF-7 and the triple-negative MDA-MB-231 breast cancer cells (ER-, PR- and HER-2/Neu not overexpressed) and A2058 melanoma cells. Cells were incubated for 72 h with different concentrations of the compounds, and then the viability was checked by the MTT assay. Colchicine was adopted as the reference molecule for these assays. The results, summarized in Table 1, indicate that all of the compounds diminished the viability of both breast cancer cell lines. In particular, compound **7e** proved the most active against the ER+ MCF-7 breast cancer cells, with an IC_50_ value of 3.1 ± 0.6 µM, followed by compound **7b,** which showed good anticancer activity as well with an IC_50_ value of 8.5 ± 1.2 µM. However, compounds **7c**, **7d** and **7f** were less able to reduce MCF-7 cell growth, showing IC_50_ values of 19.5 ± 0.6, 34.1 ± 0.7 and 17.4 ± 1.1 µM, respectively. Finally, compound **7a** did not interfere with MCF-7 cell viability until the concentration of 100 µM. All of the tested compounds were less active against the triple-negative MDA-MB-231 breast cancer cells, with the exception of compound **7a**. Particularly, **7b** showed the best anticancer activity, with an IC_50_ value of 17.9 ± 1.1 µM, whereas compound **7a** showed an IC_50_ of 27.5 ± 0.9 µM. Good anticancer activity was also found against the A2058 melanoma cells, where compound **7b** was the most active, showing an IC_50_ value of 3.5 ± 1.1 µM. Compounds **7c**, **7d** and **7e** exhibited good anticancer activity on the same melanoma cell line as well, with IC_50_ values of 4.8 ± 1.0, 6.4 ± 0.9 and 8.4 ± 0.9 µM, respectively. In contrast, compound **7f** showed a higher IC_50_ value (24.6 ± 1.1 µM), while compound **7a** did not exhibit any anticancer activity up to 100 µM. Moreover, we checked the cytotoxicity of all of the synthesized compounds against the human MCF-10A non-malignant breast epithelial cells and mouse BALB/3T3 embryonic fibroblasts, in order to evaluate their selectivity on cancer cells. Among them, only **7d** and **7e** exhibited a slight toxicity against the BALB/3T3 cells, with IC_50_ values of 36.1 ± 1.0 and 35.0 ± 0.6 µM, respectively. However, compound **7e** possessed a large therapeutic window; indeed, it was about 11-fold more active against the MCF-7 breast cancer cells than against mouse BALB/3T3 embryonic fibroblasts. Moreover, all of the compounds were non-cytotoxic against the human MCF-10A mammary epithelial cells, with the exception of compound **7c** (IC_50_ = 46.4 µM). Finally, all of the compounds exhibited less activity in comparison with the adopted reference molecules, colchicine and ellipticine; indeed, the latter had higher anticancer activity against all of the cell lines used in these assays compared to the newly synthesized compounds, but they exhibited dramatic cytotoxic effects on the normal cells (colchicine: IC_50_ = 5.2 (± 0.9) × 10^−1^ and 8.9 (± 1.2) × 10^−3^ µM against the BALB/3T3 and MCF-10A cells, respectively; ellipticine: IC_50_ = 0.97 ± 0.1 and 1.09 ± 0.1 µM against the BALB/3T3 and MCF-10A cells, respectively), contrarily to the compounds under study. It is worth noting that an improvement in anticancer activity was observed when the 4-position of the thiazole ring of compound **7a** was substituted by a phenyl- or *p*-substituted phenyl group. Indeed, compounds **7b**, **7c**, **7d** and **7e** showed higher activity than compound **7a** against MCF-7 and A2058 cells, but not against MDA-MB-231 cells. Moreover, the fusion of a benzene ring to the thiazole moiety, as in **7f**, increased the anticancer activity, even if to a lesser extent, against MCF-7 and A2058 cells. Finally, we decided to investigate the mechanism of action of the compounds that showed higher anticancer activity, **7b, 7c**, **7d** and **7e**, bearing at position 4 of their thiazole ring a non-substituted phenyl-, *p*-tolyl-, *p*-chlorophenyl-, and *p*-bromophenyl group, respectively.

#### 2.3.2. Inhibition of Human Topoisomerases I and II

**Figure 3:**
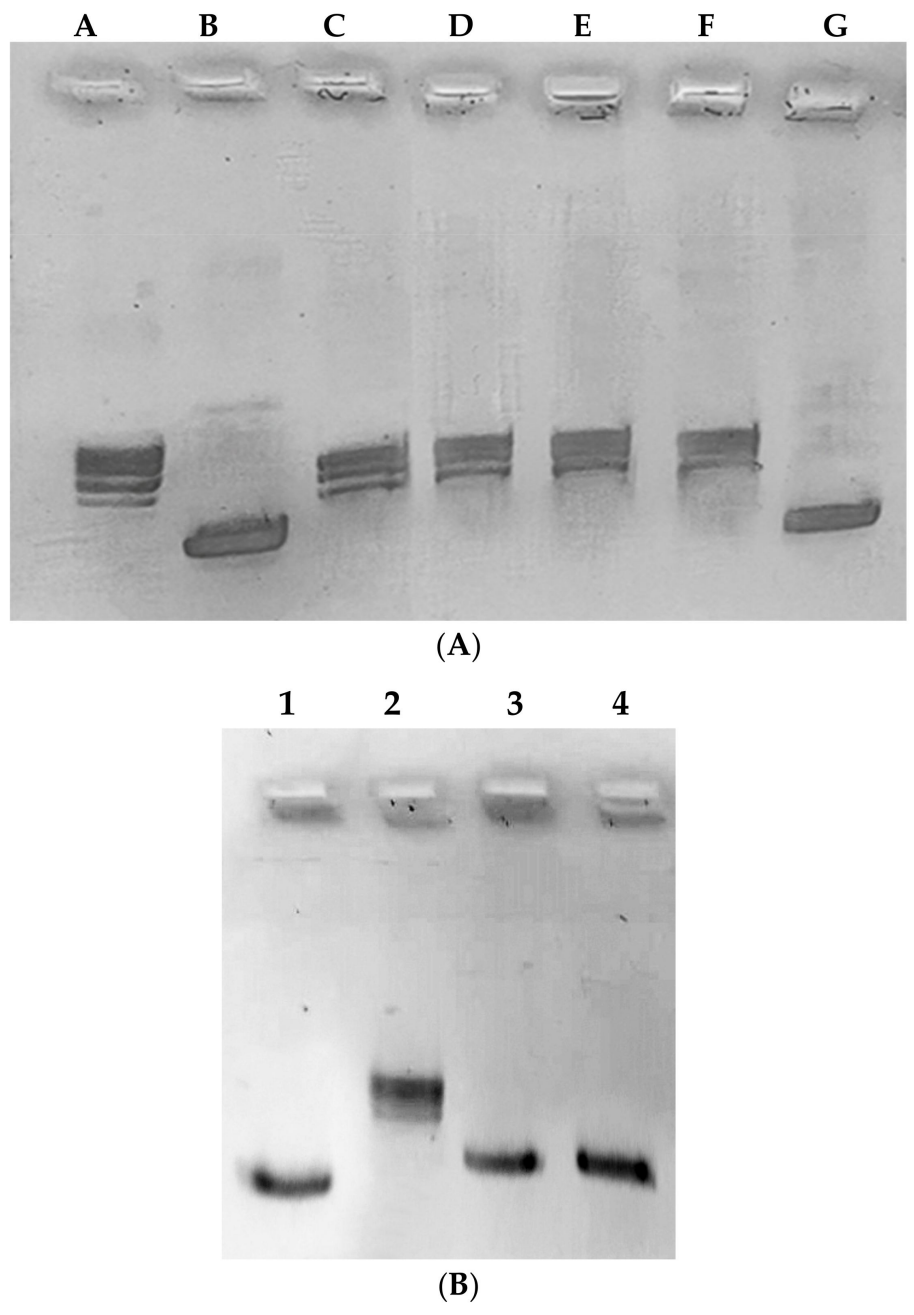
hTopo I relaxation assay. Plasmid pHOT1, used as substrate, was incubated with hTopo I with or without the studied compounds **7b**–**7e** (10 µM concentration), panel (**A**), and with or without ellipticine (10 and 50 µM concentration), panel (**B**). Lane A, relaxed DNA marker; lane B, pHOT1 supercoiled DNA, lane C, CTRL (DMSO), lane D–G, compounds **7b**–**7e** 10 µM, respectively. Lane 1, pHOT1 supercoiled DNA; lane 2, CTRL (DMSO), lanes 3–4, ellipticine 10 and 50 µM, respectively.

Thiazoles and their derivatives are extensively recognized as chemical nuclei of great interest for producing molecules with multiple biological properties [54]. In particular, several compounds bearing these heterocycles were studied for their capability to interfere with DNA topoisomerases (Topos) [54,55,56], ubiquitous enzymes essential in several key cellular processes. In recent years, these enzymes have become popular targets in cancer therapy; indeed, the topoisomerase inhibitors can induce damage to genome integrity. Consequently, the inhibition of topoisomerases leads to cell death by apoptosis and, thus, is fatal [57]. Thus, we carried out two specific tests in vitro for both of the Topos, namely the hTopo I relaxation and the hTopo II decatenation assays. For the hTopo I relaxation assay, we exposed the enzyme to the most interesting compounds, **7b**–**7e,** at the final concentration of 10 µM for 1 h at 37 °C, using as substrate the supercoiled pHOT1 (see Section 4 Materials and Methods for more details). As evidenced in Figure 3, panel A, only compound **7e** was able to inhibit hTopo I activity under the adopted experimental conditions; indeed, a single band in the lower portion of the agarose gel, representing the uncleaved plasmid pHOT1, was visible (Figure 3, lane G). Contrarily, in the CTRL lane, where the hTopo I was exposed to only vehicle (DMSO), several bands due to pHOT1 DNA cleavage were present (Figure 3, lane C). A similar behavior was obtained by incubating the enzyme with compounds **7b**, **7c** or **7d** (Figure 3, lanes D–F), indicating that these compounds were not able to inhibit the relaxation activity of the enzyme. In Figure 3, panel B, the hTopo I inhibition results using ellipticine, as reference molecule are shown. The latter was able to block the hTopo I activity at both of the used concentrations (10 and 50 µM, lanes 3 and 4, respectively). An electrophoretic mobility shift of the pHOT1 DNA was also noticeable, due to the intercalating properties of ellipticine [58]. 

**Figure 4:**
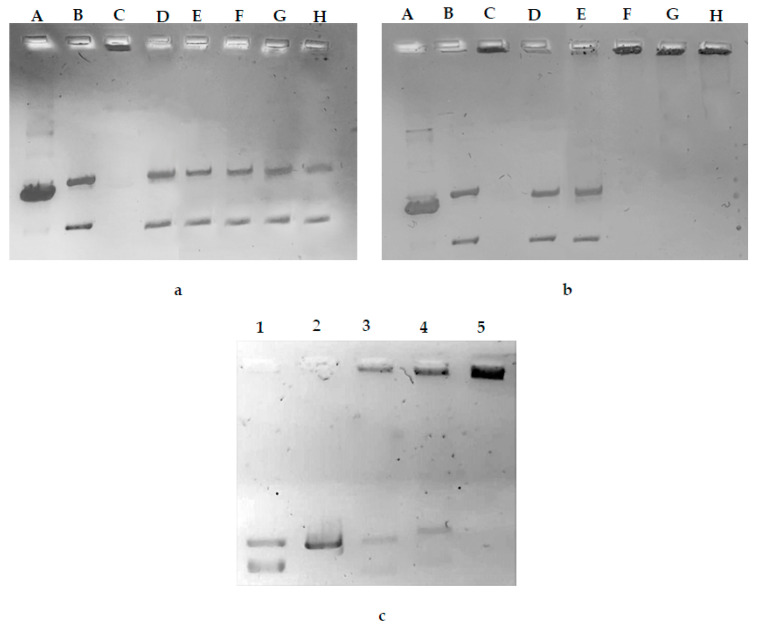
hTopo II decatenation assay. The catenated circles of kDNA, used as substrate, incubated with hTopo II with or without the studied compounds **7b**–**7e** used at the concentrations of 10 µM (**a**) and 50 µM (**b**). Lane A, linear kDNA marker; lane B, decatenated kDNA marker; lane C, catenated circles of kDNA; lane D, CTRL (DMSO), lanes E–H, compounds **7b**–**7e**, respectively. The same experiment was performed using ellipticine (**c**). Lane 1, decatenated kDNA marker; lane 2, linear kDNA marker; lane 3, CTRL (DMSO), lanes 4–5, ellipticine 10 and 50 µM, respectively.

After that, we also tested the inhibitory activity of compounds **7b**–**7e** toward hTopo II activity, exposing the enzyme to the mentioned compounds at a concentration of 10 µM for 1 h at 37 °C (Figure 4a). We found that none of the compounds were capable of blocking hTopo II activity, as evidenced by the two DNA bands at the bottom of the gel (decatenation products of kDNA, used as substrate, Figure 4a, lanes E–H). The same pattern was visible in the CTRL reaction, in which the enzyme was incubated with DMSO only, and for the decatenated kDNA marker (Figure 4a, lanes D and B, respectively). However, increasing the compound concentrations to 50 µM (Figure 4b), compounds **7c**–**7e** fully blocked the enzyme activity, as confirmed by a clear band in the upper of the gel that is the catenated kDNA (Figure 4b, lanes F–H). On the contrary, the compound **7b** was unable to inhibit the hTopo II activity (Figure 4b, lane E). The linear kDNA (Figure 4a,b, lanes A), decatenated kDNA (Figure 4a,b, lanes B) and the catenated circles of kDNA (Figure 4a,b, lanes C) were used as markers. A parallel experiment was performed using ellipticine as reference molecule. As visible in Figure 4c, ellipticine was able to totally block the hTopo II activity only at 50 µM and partially at 10 µM (lanes 4 and 5, respectively). As well in these experiments, ellipticine intercalates the decatenated kDNA products, producing their electrophoretic mobility shift. Finally, the obtained hTopo I and II inhibition results were resumed in Table 2.

#### 2.3.3. Compound **7e** Induced Apoptosis via Cytochrome c Release in MCF-7 Cells

**Figure 5:**
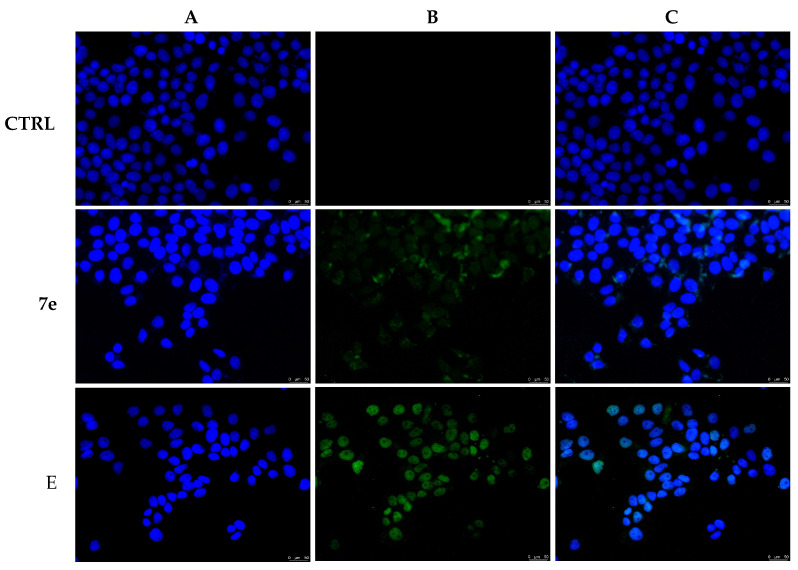
TUNEL assay. MCF-7 breast cancer cells were exposed to compounds **7e** and ellipticine (E) at their IC_50_ values, or to the vehicle (DMSO) for 24 h. Then, they were further processed, as reported in Section 4, and imaged under a fluorescence microscope (20× magnification). Panels (**A**), DAPI λ_ex,em_= 350 nm/460 nm; panels (**B**), CF^TM^488A, λ_ex,em_ = 490 nm/515 nm; panels (**C**) show the merge channel.

Numerous data in the literature indicate that topoisomerase inhibitors are among the most efficient inducers of apoptosis [59,60,61]. Thus, we carried out the TUNEL assay to assess the capability of compound **7e**, which was the most active compound and also the only one able to inhibit both of the hTopos, to trigger apoptosis. As reference molecule, we adopted ellipticine for this assay, also. As shown in Figure 5, the green nuclear fluorescence indicated that MCF-7 cells treated with compound **7e,** at its IC_50_ value, had undergone the apoptotic process (Figure 5, **7e**, panel B). Ellipticine exhibited similar behavior (Figure 5, E, panel B). In contrast, no fluorescence was visible in the DMSO-treated cells used as negative control (Figure 5, CTRL, panel B). These outcomes indicated the ability of the studied compound to induce DNA damage, as ellipticine did, triggering cancer cells death. 

**Figure 6:**
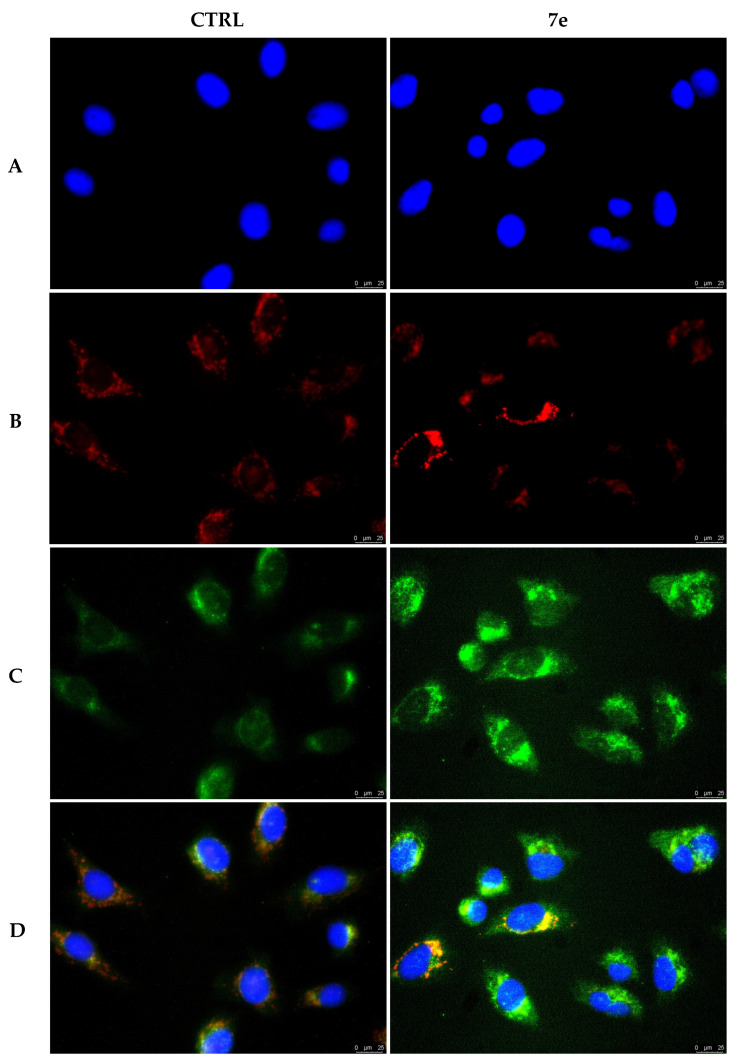
Mitochondria and cytochrome c immunostaining in MDA-MB-231 cells. In DMSO-treated cells, cytochrome c (panel (**C**), CTRL) resides within mitochondria (panel (**B**), CTRL), as visible in the overlay channel (panel (**D**), CTRL). MCF-7 cells treated with **7e** (concentration equal to its IC_50_ value, for 24 h) show cytochrome c release in the cytosol (panel (**C**), **7e**) and changes in mitochondrial structure (panel (**B**), **7e**). (**A**) Nuclear stain with DAPI (excitation/emission wavelength 350 nm/460 nm). (**B**) MitoTracker Deep Red FM probe (excitation/emission wavelength 644 nm/665 nm). (**C**) Alexa Fluor CF 488 (excitation/emission wavelength 490 nm/515 nm). (**D**) Merge channels. Images were taken at 40× magnification, and representative fields are shown.

In order to establish whether the observed cell death could be ascribed to the induction of the apoptotic pathway, we performed immunostaining assays using an anti-cytochrome c (cyt c) antibody and the mitochondrial probe MitoTracker Deep Red FM, which would allow us to verify whether cyt c was released from mitochondria to the cell cytoplasm or not. In Figure 6, panels B,C, it is possible to visualize the intracellular localization of cyt c (green fluorescence) and mitochondria (red fluorescence). In MCF-7 cells exposed to only the vehicle (DMSO), there was a perfect merge between the two fluorescence signals (panel D), indicating that cyt c resided within the mitochondrial compartment. On the contrary, under compound **7e** exposure, the mitochondria became piled up (panel C) and, most importantly, the cyt c was released into the cell cytoplasm, as indicated by the rise in a diffused green fluorescence (panel B). Indeed, as visible in the overlay channel (panel D), the two fluorescence signals (red and green) were no longer superimposable.

**Figure 7:**
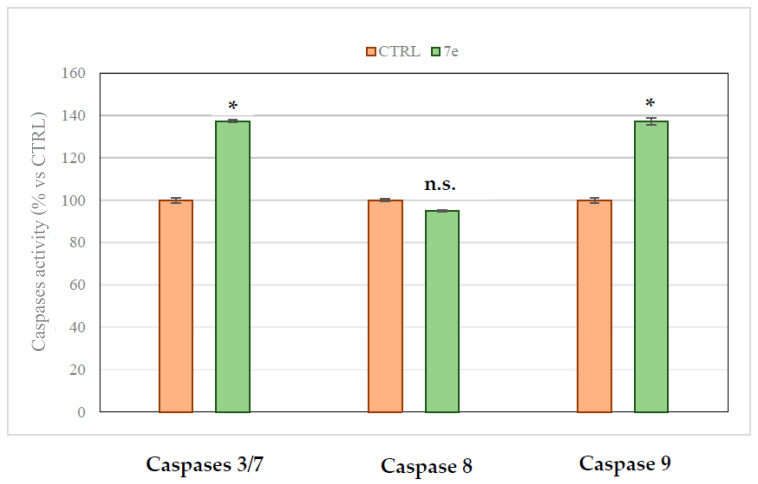
Caspase activity in MCF-7 cells. The treatment of MCF-7 cells with the compound **7e**, at a concentration equal to its IC_50_ value for 24 h, induced caspase 3/7 and 9 activation (compared to DMSO-treated cells, CTRL). Columns mean, bars SD; * *p* < 0.001; n.s., not significant (treated vs. CTRL).

Once established that mitochondria were damaged and cyt c released into the cytoplasm, we evaluated the ability of compound **7e** treatment (used at its IC50 value) in modulating caspase activity. Figure 7 shows that there was a noticeable increase in caspase-9 activity and no change in caspase-8 activity in MCF-7 cells treated with compound **7e** compared to the vehicle-treated ones. Furthermore, MCF-7 cells treated with compound **7e** exhibited higher activity of caspases 3/7 (versus vehicle-treated cells), which are substrates of the initiator caspase-9. Altogether, these data suggest that compound **7e** is able to trigger the intrinsic apoptotic pathway in MCF-7 cells.

## 3. Discussion

Cancer therapies have been subjected to a considerable evolution in recent years, contributing to improve the quality of life and survival of patients with cancer. These advances mirror the efforts made by governments and research institutes worldwide, and the clinical use of chemotherapy has been significantly improved using personalized dosing regimens and combination therapies. Moreover, the advent of targeted therapy revolutionized cancer treatment and, for this purpose, drug design strategies still play an essential role in drug discovery and development [62]. An important strategy in medicinal chemistry is hybrid drug design, which integrates different compounds or pharmacophores together, generating new bioactive molecules [63]. Herein, we present the synthesis of new thiazolidinedione scaffolds containing three pharmacophores, namely trimethoxybenzene, thiazolidinedione and thiazole, in a single hybrid molecule. Several studies already reported the anticancer potential of the above-mentioned pharmacophores [16,64,65], but in different cases, several issues, such as low activity and eventually, selectivity or the lack of a specific target, were raised. In some cases, the hybrid derivatives may represent a valid alternative, in particular, our compounds were proved to exert good anticancer activity, particularly against the MCF-7 cells, and most interestingly, without affecting the viability of the tested normal cells. This is a very promising parameter to consider, since one of the major limitations of current anticancer chemotherapy regimens is the high incidence of side effects. Moreover, by means of in silico and in vitro assays, we individuated two intracellular targets, i.e., the human topoisomerases I and II, as candidates responsible for the observed anticancer properties. The importance of these enzymes in cancer onset and progression is currently being ascertained, and a still growing number of studies are focused on this [66]. Most importantly, targeting both of the enzymes, our compounds could also diminish the frequent onset of resistance phenomena related to the compensative pathways that cancer cells may trigger [67]. The blockade of these enzymes produced DNA damage, as demonstrated by the TUNEL assays, and induced death of the cancer cells through the intrinsic apoptotic pathway. Indeed, the treatment with 7e promoted the activation of caspases 3/7 and 9, mitochondria destabilization and cyt c migration into the cytoplasm. Summing up, we are confident that our outcomes may bring an important contribution to the field of hybrid drug design, to be further developed for cancer chemotherapy.

## 4. Materials and Methods

### 4.1. Chemistry

#### 4.1.1. General Methods

All melting points were determined on a Stuart melting point apparatus SMP3 and are uncorrected. IR spectra were recorded on a Nicolet iS10 FT-IR spectrometer using the KBr wafer technique (Thermo Fisher, Waltham, MA, USA). The ^1^H-NMR spectra were recorded on a Bruker Avance III spectrometer operating at 400 MHz (^1^H) or 100 MHz (^13^C) (Bruker, Billerica, MA, USA). ^1^H- and ^13^C-NMR chemical shifts were reported in parts per million (ppm) and were referenced to the residual proton peaks in a deuterated solvent, DMSO-*d*_6_ (2.50 ppm for ^1^H and 39.70 ppm for ^13^C). Multiplicities are represented by s (singlet), d (doublet), t (triplet), q (quartet), and m (multiplet). Coupling constants (*J*) are reported in Hertz (Hz). The reactions were monitored by thin-layer chromatography (TLC). Elemental analyses (C, H, N) were conducted using a Perkin Elmer 240 C Microanalyzer (Perkin Elmer, Waltham, MA, USA) and found to be in good agreement with the theoretical values within ± 0.4%. 2-Aminothiazole and 2-aminobenzothiazoles were purchased from Sigma-Aldrich (St. Louis, MO, USA). 1,3-thiazolidine-2,4-dione (1), (*Z*)-5-(3,4,5-trimethoxy benzylidene)-1,3-thiazolidine-2,4-dione (2) and (Z)-5-(3,4,5-trimethoxy benzylidene)-1,3-thiazolidine-2,4-dione potassium salt (3) were prepared as described in our previous publication [43]. 

#### 4.1.2. *Preparation of 2-amino-4-aryl-1,3-thiazoles **4b–e***

These compounds were prepared according to the literature [50]. To a slurry consisting of thiourea (1.522 g, 0.02 mol), acetophenone (1.1 mL, 0.01 mol), 4-methylacetophenone (1.34 g, 0.01 mol), 4-chloroacetophenone (1.25 mL, 0.01 mol), or 4-bromoacetophenone (1.9 g, 0.01 mol),iodine (2.5 g, 0.01 mol) were added all at once. The reaction mixture was heated on a boiling water bath overnight with occasional swirling. The reddish reaction product obtained was diluted with water, heated under boiling until a nearly clear solution was obtained. This was filtered, cooled and made alkaline with conc. NH_4_OH solution. The yellow product was filtered, washed with water, dried and crystallized from ethanol. 

*2-Amino-4-phenylthiazole* (**4b**). Yellow crystals, yield 1.7 g (99%), mp 145–147 °C (lit. [50] 147 °C). **IR** (KBr, cm^−1^): 3435 and 3250 (*ν* NH_2_), 3114 (*ν* C-H arom.), 1637 (δNH_2_).

*2-Amino-4-(4-methylphenyl)thiazole* (**4c**). Yellow crystals, yield 1.6 g (87%), mp 135–137 °C (lit. [68] 135–136 °C). **IR** (KBr, cm^−1^) 3454 and 3297 (*ν* NH_2_), 3117 (*ν* C-H arom.), 2974 (*ν* C-H aliph.), 1636 δNH_2_). 

*2-Amino-4-(4-chlorolphenyl)thiazole* (**4d**). Yellow crystals, yield 1.5 g (73%), mp 167–169 °C (lit. [68] 167–168 °C). **IR** (KBr, cm^−1^) 3438 and 3283 (*ν* NH_2_), 3111 (*ν* C-H arom.), 2987 (*ν* C-H aliph.), 1633 δNH_2_). 

*2-Amino-4-(4-bromolphenyl)thiazole* (**4e**). Yellow crystals, yield 2.5 g (99%), mp 180–183 °C (lit. [69] 179–181 °C). **IR** (KBr, cm^−1^) 3428, 3281 (*ν* NH_2_), 3111 (*ν* C-H arom.), 2987 (*ν* C-H aliph.) and 1633 (δNH_2_). 

#### 4.1.3. *Chloroacetylation of 2-Aminothiazoles; Preparation of 2-chloroacetamides **5a–f***

To a cold solution 0–5 °C of **4a**–**f** (0.002 mol) in dry benzene, a cold solution of chloroacetyl chloride (0.0033 mol) in dry benzene was added dropwise with stirring. The reaction mixture was then heated in a boiling water bath for 3 h. After cooling, the reaction was evaporated under reduced pressure until dryness. The solid product obtained was washed with 5% NaHCO_3_, then with water, filtered, dried and crystallized from ethanol.

*2-Chloro-N-(thiazol-2-yl)acetamide* (**5a**). White needles, yield 0.759 g (86%), mp 171–172 °C (lit. [70] 170–170.5 °C). **IR** (KBr, cm^−1^): 3186 (*ν* N-H), 3081 (*ν* C-H arom.), 2949 and 2882 (*ν* C-H aliph.), 1703 (*ν* C=O). **^1^H**-**NMR** (400 MHz, DMSO-*d*_6_) δ (ppm): 10.53 (s, 1H, NH), 7.45 (s, 1H, thiazole-H), 7.19 (s, 1H, thiazole-H), 4.20 (s, 2H, CH_2_). Anal. calcd. for C_5_H_5_ClN_2_OS (176.62): C, 34.00; H, 2.85; N, 15.86; S, 18.15. Found: C, 34.18; H, 2.79; N, 15.92; S, 18.25%.

*2-Chloro-N-(4-phenylthiazol-2-yl)acetamide* (**5b**). Colorless crystals, yield 0.327 g (65%), mp 150–151 °C (lit. [70] 164 °C). **IR** (KBr, cm^−1^): 3181 (*ν* N-H), 3022 (*ν* C-H arom.), 2983 (*ν* C-H aliph.), 1655 (*ν* C=O). **^1^H**-**NMR** (400 MHz, DMSO-*d*_6_) δ (ppm): 9.95 (s, 1H, NH), 7.76 (d, 2H, *J* = 7.2 Hz, Ph-H), 7.35 (t, 2H, *J* = 7.3 Hz, Ph-H), 7.27 (m, 1H, Ph-H), 7.12 (s, 1H, thiazole-H), 4.13 (s, 2H, CH_2_). Anal. calcd. for C_11_H_9_ClN_2_OS (252.72): C, 52.28; H, 3.59; N, 11.09; S, 12.69. Found: C, 52.39; H, 3.43; N, 11.39; S, 12.73%.

*2-Chloro-N-(4-methylphenylthiazol-2-yl)acetamide* (**5c**). Colorless crystals, yield 0.499 g (94%), mp 162–163 °C (lit. [69] 157–158 °C). **IR** (KBr, cm^−1^): 3157 (*ν* N-H), 3047 (*ν* C-H arom.), 2921 (*ν* C-H aliph.), 1700 (*ν* C=O). **^1^H**-**NMR** (400 MHz, DMSO-*d*_6_) δ (ppm): 10.07 (s, 1H, NH), 7.63 (d, 1H, *J* = 7.8 Hz, Ph-H), 7.15 (d, 1H, *J* = 7.8 Hz, Ph-H), 7.05 (s, 1H, thiazole-H), 4.08 (s, 2H, CH_2_), 2.30 (s, 3H, CH_3_). Anal. calcd. for C_12_H_11_ClN_2_OS (266.74): C, 54.03; H, 4.16; N, 10.50; S, 12.02. Found: C, 54.13; H, 4.01; N, 10.41; S, 12.11%.

*2-Chloro-N-(4-chlorophenylthiazol-2-yl)acetamide* (**5d**). Colorless crystals, yield 0.48 g (86%), mp 175–177 °C (lit. [71] 170–173 °C). **IR** (KBr, cm^−1^): 3373 (*ν* N-H), 3109 (*ν* C-H arom.), 2988 (*ν* C-H aliph.), 1692 (*ν* C=O). **^1^H**-**NMR** (400 MHz, DMSO-*d*_6_) δ (ppm): 9.82 (s, 1H, NH), 7.68 (d, 2H, *J* = 8.3 Hz, Ph-H), 7.30 (d, 2H, *J* = 8.3 Hz, Ph-H), 7.09 (s, 1H, thiazole-H), 4.17 (s, 2H, CH_2_). Anal. calcd. for C_11_H_8_Cl_2_N_2_OS (287.16): C, 46.01; H, 2.81; N, 9.76; S, 11.16. Found: C, 46.10; H, 2.89; N, 9.84; S, 11.21%.

*2-Chloro-N-(4-bromophenylthiazol-2-yl)acetamide* (**5e**). Colorless crystals, yield 0.408 g (62%), mp 174–175 °C (lit. [69] 169–172 °C). **IR** (KBr, cm^−1^): 3366 (*ν* N-H), 3109 (*ν* C-H arom.), 2940 (*ν* C-H aliph.), 1692 (*ν* C=O). **^1^H**-**NMR** (400 MHz, DMSO-*d*_6_) δ (ppm): 9.75 (s, 1H, NH), 7.62 (d, 2H, *J* = 8.4 Hz, Ph-H), 7.46 (d, 2H, *J* = 8.4Hz, Ph-H), 7.11 (s, 1H, thiazole-H), 4.17 (s, 2H, CH_2_). Anal. calcd. for C_11_H_8_BrClN_2_OS (331.61): C, 39.84; H, 2.43; N, 8.45; S, 9.67. Found: C, 39.91; H, 2.39; N, 8.44; S, 9.70%.

*2-Chloro-N-(benzo[d]thiazol-2-yl)acetamide* (**5f**). White crystals, yield 0.424 g (94%), mp 161–162 °C (lit. [72] 140–143 °C). **IR** (KBr, cm^−1^): 3507 (*ν* NH), 3048 (*ν* CH arom.), 2986 (*ν* CH aliph.), 1695 (*ν* C=O). **^1^H**-**NMR** (400 MHz, DMSO-*d*_6_) δ (ppm): 9.81 (s, 1H, NH), 7.76 (t, 2H, *J* = 7.0 Hz, Ph-H), 7.40 (t, 1H, *J* = 7.3 Hz, Ph-H), 7.28 (t, 1H, *J* = 7.2 Hz, Ph-H), 4.23 (s, 2H, CH_2_). Anal. calcd. for C_9_H_7_ClN_2_OS (226.68): C, 47.69; H, 3.11; N, 12.36; S, 14.14. Found: C, 47.78; H, 3.00; N, 12.44; S, 14.04%.

#### 4.1.4. *Preparation of 2-Iodoacetamides **6a–f***

A mixture of **5a**–**f** (5 mmole) and KI (6 mmole) in acetone (50 mL) was heated under reflux for 4 h. The solvent was then evaporated under reduced pressure. The solid product obtained was washed thoroughly with water, filtered and recrystallized from ethanol. 

*2-Iodo-N-(thiazol-2-yl)acetamide* (**6a**). Colorless crystals, yield 0.925 g (69%), m.p 175.9–177.5 °C. **IR** (KBr, cm^−1^): 3166 (*ν* N-H), 3107 (*ν* C-H arom.), 2964 (*ν* C-H aliph.), 1647 (*ν* C=O). **^1^H**-**NMR** (400 MHz, DMSO-*d*_6_) δ (ppm): 12.42 (s, 1H, NH), 7.50 (d, *J* = 3.5 Hz, 1H, Ph-H), 7.26 (d, *J* = 3.5 Hz, 1H, Ph-H), 3.93 (s, 2H, CH_2_). **^13^C**-**NMR** (100 MHz, DMSO-*d*_6_) δ (ppm): 165.8 (C=O), 159.7 (C), 134.9 (CH), 114.2 (CH), 3.9 (CH_2_). Anal. calcd. for C_5_H_5_IN_2_OS (268.07): C, 22.40; H, 1.88; N, 10.45; S, 11.96. Found: C, 22.19; H, 1.77; N, 10.50; S, 11.71%.

*2-Iodo-N-(4-phenylthiazol-2-yl)acetamide* (**6b**). Colorless crystals, yield 1.585 g (92%), m.p 188–190 °C. **IR** (KBr, cm^−1^): 3174 (*ν* N-H), 3039 (*ν* C-H arom.), 2988 (*ν* C-H aliph.), 1648 (*ν* C=O). **^1^H**-**NMR** (400 MHz, DMSO-*d*_6_) δ (ppm): 12.60 (s, 1H, NH), 7.90 (d, 2H, *J* = 7.2 Hz, Ph-H), 7.67 (s, 1H, thiazole-H), 7.44 (t, 2H, *J* = 7.6 Hz, Ph-H), 7.33 (t, 1H, *J* = 7.3 Hz, Ph-H), 3.97 (s, 2H, CH_2_). **^13^C**-**NMR** (100 MHz, DMSO-*d*_6_) δ (ppm): 167.9 (C=O), 158.4 (C), 149.7 (C), 134.8 (C), 129.5 (2CH), 128.6 (CH), 126.4 (2CH), 109.2 (CH), −0.51 (CH_2_). Anal. calcd. for C_11_H_9_IN_2_OS (344.17): C, 38.39; H, 2.64; N, 8.14; S, 9.32. Found: C, 38.43; H, 2.57; N, 8.20; S, 9.25%.

*2-Iodo-N-(4-methylphenylthiazol-2-yl)acetamide* (**6c**). Colorless crystals, yield 1.535 g (89%), m.p 180–182 °C. **IR** (KBr, cm^−1^): 3182 (*ν* N-H), 3073 (*ν* C-H arom.), 2972 (*ν* C-H aliph.), 1646 (*ν* C=O), **^1^H**-**NMR** (400 MHz, DMSO-*d*_6_) δ (ppm): 12.58 (s, 1H, NH), 7.78 (d, *J* = 8.1 Hz, 2H, Ph-H), 7.56 (s, 1H, thiazole-H), 7.23 (d, 2H, *J* = 8.0 Hz, Ph-H), 3.97 (s, 2H, CH_2_), 2.31 (s, 3H, CH_3_). **^13^C**-**NMR** (100 MHz, DMSO-*d*_6_) δ (ppm): 167.8 (C=O). 158.2 (C), 149.8 (C), 137.8 (C), 132.1 (C), 130.1 (2CH), 126.3 (2CH), 108.2 (CH), 21.5 (CH_3_), −0.51 (CH_2_). Anal. calcd. for C_12_H_11_IN_2_OS (358.20): C, 40.24; H, 3.10; N, 7.82; S, 8.95. Found: C, 40.33; H, 3.22; N, 7.88; S, 9.01%. 

*2-Iodo-N-(4-chlorophenylthiazol-2-yl)acetamide* (**6d**). Colorless crystals, yield 1.75 g (70%), m.p 184.7–186 °C. **IR** (KBr, cm^−1^): 3181 (*ν* N-H), 3071 (*ν* C-H arom.), 2974 (*ν* C-H aliph.), 1652 (*ν* C=O). **^1^H**-**NMR** (400 MHz, DMSO-*d*_6_) δ (ppm): 12.61 (s, 1H, NH), 7.91 (d, 2H, *J* = 8.6 Hz, Ph-H), 7.73 (s, 1H, thiazole-H), 7.49 (d, 2H, *J* = 8.6 Hz, Ph-H), 3.96 (s, 2H, CH_2_). **^13^C**-**NMR** (100 MHz, DMSO-*d*_6_) δ (ppm): 168.0 (C=O). 158.7 (C), 148.6 (C), 138.8 (C), 133.1 (C), 129.5 (2CH), 128.1 (2CH), 110.6 (CH), −0.51 (CH_2_). Anal. calcd. for C_11_H_8_ClIN_2_OS (378.61): C, 34.90; H, 2.13; N, 7.40; S, 8.47. Found: C, 35.11; H, 2.09; N, 7.49; S, 8.51%.

*2-Iodo-N-(4-bromophenylthiazol-2-yl)acetamide* (**6e**). Colorless crystals, yield 1.89 g (89%), m.p 192.7–194 °C. **IR** (KBr, cm^−1^): 3179 (*ν* N-H), 3072 (*ν* C-H arom.), 2974 (*ν* C-H aliph.), 1652 (*ν* C=O). ^**1**^**H**-**NMR** (400 MHz, DMSO-*d*_6_) δ (ppm): 12.61 (s, 1H, NH), 7.85 (d, 2H, *J* = 8.6 Hz, Ph-H), 7.74 (s, 1H, thiazole-H), 7.63 (d, 2H, *J* = 8.6 Hz, Ph-H), 3.93 (s, 2H, CH_2_). **^13^C**-**NMR** (100 MHz, DMSO-*d*_6_) δ (ppm): 168.0 (C=O), 158.7 (C), 148.7 (C), 134.2 (C), 132.6 (2CH), 128.5 (2CH), 121.7 (C), 110.1 (CH), −0.51 (CH_2_). Anal. calcd. for C_11_H_8_BrIN_2_OS (423.07): C, 31.23; H, 1.91; N, 6.62; S, 7.58. Found: C, 31.32; H, 1.82; N, 6.69; S, 7.60%.

*2-Iodo-N-(benzo[d]thiazol-2-yl)acetamide* (**6f**). Yellow crystals, yield 1.295 g (82%), m.p 182–184 °C. **IR** (KBr, cm^−1^): 3171 (*ν* N-H), 3057 (*ν* C-H arom.), 2989 (*ν* C-H aliph.) and 1656 (*ν* C=O). **^1^H**-**NMR** (400 MHz, DMSO-*d*_6_) δ (ppm): 12.67 (s, 1H, NH), 7.98 (d, 1H, *J* = 7.8 Hz, Ph-H), 7.76 (d, 1H, *J* = 8.0 Hz, Ph-H), 7.44 (t, 1H, *J* = 7.2 Hz, Ph-H), 7.31 (t, 1H, *J* = 7.2 Hz, Ph-H), 3.99 (s, 2H, CH_2_). **^13^C**-**NMR** (100 MHz, DMSO-*d*_6_) δ (ppm): 171.6 (C), 167.5 (C=O), 158.5 (C), 158.1 (C), 138.5 (CH), 138.3 (CH), 114.6 (CH), 114.2 (CH), −0.51 (CH_2_). Anal. calcd. for C_9_H_7_IN_2_OS (318.13): C, 33.98; H, 2.22; N, 8.81; S, 10.08. Found: C, 34.05; H, 2.29; N, 8.90; S, 10.11%.

#### 4.1.5. *General Procedure for Synthesis of the Target Compounds **7a–f***

A mixture of the potassium salt **3** (0.333 g, 1 mmole) and **6a**–**f** (1 mmole) in DMF (5 mL) was heated on a boiling water bath overnight. After cooling, the reaction mixture was diluted with ice-cold water. The solid product obtained was collected and recrystallized from a mixture of ethanol/dioxane. 

*(Z)-2-(5-(3,4,5-Trimethoxybenzylidene)thiazolidin-2,4-dion-3-yl)-N-(thiazol-2-yl)acetamide (**7a**).*

Yellow crystals, yield 0.3 g (69%), mp 245–247 °C. **IR** (KBr, cm^−1^): 3189 (*ν* N-H), 3093 (*ν* C-H arom.), 2936 (*ν* C-H aliph.), 1742 and 1685 (*ν* C=O), 1604, 1577, 1504 and 1453 (C=C arom). **^1^H**-**NMR** (400 MHz, DMSO-*d*_6_) δ (ppm): 12.58 (s, 1H, NH), 7.94 (s, 1H, -CH=), 7.50 (d, 1H, *J* = 3.4 Hz, thiazole-H), 7.25 (d, 1H, *J* = 3.4 Hz, thiazole-H), 6.98 (s, 2H, Ph-H), 4.65 (s, 2H, CH_2_), 3.86 (s, 6H, 2-OCH_3_), 3.76 (s, 3H, -OCH_3_). **^13^C**-**NMR** (100 MHz, DMSO-*d*_6_) δ (ppm): 167.5 (C=O), 165.5 (C=O), 164.8 (C=O), 157.9 (C), 153.7 (2 C), 140.3 (CH), 138.2 (C), 134.4 (CH), 128.7 (C), 120.3 (C), 114.4 (CH), 108.3 (2 CH), 60.7 (OCH_3_), 56.5 (2 OCH_3_), 43.8 (CH_2_). Anal. calcd. for C_18_H_17_N_3_O_6_S_2_ (435.47): C, 49.65; H, 3.93; N, 9.65; S, 14.73. Found: C, 49.88; H, 4.18; N, 9.77; S, 15.03%.

*(Z)-2-(5-(3,4,5-Trimethoxybenzylidene)thiazolidin-2,4-dion-3-yl)-N-(4-phenylthiazol-2-yl)acetamide (**7b**).*

Yellow crystals, yield 0.48 g (94%), mp 222–224 °C. **IR** (KBr, cm^−1^): 3196 (*ν* N-H), 3077 (*ν* C-H arom.), 2974 (*ν* C-H aliph.), 1748 and 1693 (*ν* C=O). **^1^H**-**NMR** (400 MHz, DMSO-*d*_6_): δ (ppm): 12.75 (s, 1H, NH), 7.95 (s, 1H, thiazole-H), 7.91 (d, 2H, *J* = 7.5 Hz, Ph-H), 7.65 (s, 1H, -CH=), 7.44 (t, 2H, *J* = 7.5 Hz, Ph-H), 7.34 (t, 1H, *J* = 7.2 Hz, Ph-H), 6.98 (s, 2H, Ph-H), 4.69 (s, 2H, CH_2_), 3.86 (s, 6H, 2 -OCH_3_), 3.77 (s, 3H, -OCH_3_). **^13^C**-**NMR** (100 MHz, DMSO-*d*_6_) δ (ppm): 167.5 (C=O), 165.5 (C=O), 157.8 (C), 165.1 (C=O), 153.7 (2 C), 149.5 (C), 140.3 (CH), 134.6 (C), 134.4 (C), 129.2 (C), 128.7 (2 CH), 128.3 (CH), 126.1 (2 CH), 120.3 (C), 108.9 (CH), 108.3 (2 CH), 60.7 (OCH_3_), 56.5 (2 OCH_3_), 43.8 (CH_2_). Anal. calcd. for C_24_H_21_N_3_O_6_S_2_ (511.57): C, 56.35; H, 4.14; N, 8.21; S, 12.54. Found: C, 56.56; H, 4,27; N, 8.15; S, 12,71%.

*(Z)-2-(5-(3,4,5-Trimethoxybenzylidene)thiazolidin-2,4-dion-3-yl)-N-(4-(4-methylphenyl)thiazol-2-yl)acetamide (**7c**).*

Yellow crystals, yield 0.48 g (93%), mp 213–214 °C. **IR** (KBr, cm^−1^): 3190 (*ν* N-H), 3070 (*ν* C-H arom.), 2962 (*ν* C-H aliph.), 1738 and 1687 (*ν* C=O). **^1^H**-**NMR** (400 MHz, DMSO-*d*_6_): δ (ppm) 12.71 (s, 1H, NH), 7.96 (s, 1H, thiazole-H), 7.80 (d, 2H, *J* = 7.6 Hz, Ph-H), 7.58 (s, 1H, -CH=), 7.25 (d, 2H, *J* = 7.7 Hz, Ph-H), 7.00 (s, 2H, Ph-H), 4.67 (s, 2H, CH_2_), 3.87 (s, 6H, 2 -OCH_3_), 3.77 (s, 3H, -OCH_3_), 2.34 (s, 3H, CH_3_). **^13^C**-**NMR** (100 MHz, DMSO-*d*_6_): δ (ppm) 167.5 (C=O), 165.6 (C=O), 165.2 (C=O), 157.6 (C), 153.7 (2 C), 149.6 (C), 140.3 (CH), 137.7 (C), 134.4 (C), 131.9 (C), 129.7 (C), 128.7 (2 C), 126.1 (2 CH), 120.4 (C), 108.3 (CH), 108.1 (2 CH), 60.7 (OCH_3_), 56.5 (2 OCH_3_), 43.8 (CH_2_), 21.2 (CH_3_). MS (ESI) *m*/*z* 525.25. Anal. calcd. for C_25_H_23_N_3_O_6_S_2_ (525.59): C, 57.13; H, 4.41; N, 7.99; S, 12.20. Found: C, 57.33; H, 4.59; N, 8.11; S, 12.31%.

*(Z)-2-(5-(3,4,5-Trimethoxybenzylidene)thiazolidin-2,4-dion-3-yl)-N-(4-(4-chlorophenyl)thiazol-2-yl)acetamide (**7d**).*

Yellow crystals, yield 0.52 g (95%), mp 243–246 °C. **IR** (KBr, cm^−1^): 3188 (*ν* N-H), 3065 (*ν* C-H arom.), 2962 (*ν* C-H aliph.), 1736 and 1685 (*ν* C=O). **^1^H**-**NMR** (400 MHz, DMSO-*d*_6_): δ (ppm) 12.80 (s, 1H, NH), 7.95 (s, 1H, thiazole-H), 7.92 (d, 2H, *J* = 8.5 Hz, Ph-H), 7.74 (s, 1H, -CH=),7.50 (d, 2H, *J* = 8.5 Hz, Ph-H), 6.99 (s, 2H, Ph-H), 4.67 (s, 2H, CH_2_), 3.86 (s, 6H, 2 -OCH_3_), 3.75 (s, 3H, -OCH_3_). **^13^C**-**NMR** (100 MHz, DMSO-*d*_6_): δ (ppm) 167.5 (C=O), 165.6 (C=O), 165.2 (C=O), 157.9 (C), 153.7 (2 C), 148.2 (C), 140.2 (CH), 134.4 (C), 133.4 (C), 132.8 (C), 129.2 (C), 128.7 (2 CH), 127.8 (2 CH), 120.3 (C), 109.7 (CH), 108.2 (2 CH), 60.7 (OCH_3_), 56.5 (2 OCH_3_), 43.8 (CH_2_). Anal. calcd. for C_24_H_20_ClN_3_O_6_S_2_ (546.02): C, 52.79; H, 3.69; N, 7.70; S, 11.75. Found: C, 52.94; H, 3.98; N, 7.85; S, 11.99%.

*(Z)-2-(5-(3,4,5-Trimethoxybenzylidene)thiazolidin-2,4-dion-3-yl)-N-(4-(4-bromophenyl)thiazol-2-yl)acetamide (**7e**).*

Yellow crystals, yield 0.58 g (97%), mp 250–251 °C. **IR** (KBr, cm^−1^): 3248 (*ν* N-H), 3097 (*ν* C-H arom.), 2941 (*ν* C-H aliph.), 1741 and 1682 (*ν* C=O). **^1^H**-**NMR** (400 MHz, DMSO-*d*_6_): δ (ppm) 12.76 (s, 1H, NH), 7.96 (s, 1H, thiazole-H), 7.85 (d, 2H, *J* = 8.1 Hz, Ph-H), 7.74 (s, 1H, -CH=),7.63 (d, 2H, *J* = 8.2 Hz, Ph-H), 6.99 (s, 2H, Ph-H), 4.68 (s, 2H, CH_2_), 3.86 (s, 6H, 2 -OCH_3_), 3.77 (s, 3H, -OCH_3_). **^13^C**-**NMR** (100 MHz, DMSO-*d*_6_): δ (ppm) 167.5 (C=O), 165.5 (C=O), 165.2 (C=O), 158 (C), 153.7 (2 C), 148.3 (C), 140.3 (CH), 134.4 (C), 133.8 (2 CH), 132.1 (C), 128.7 (C), 128.1 (2 CH), 121.4 (C), 120.3 (C), 109.8 (CH), 108.3 (2 CH), 60.7 (OCH_3_), 56.6 (2 OCH_3_), 43.8 (CH_2_). Anal. calcd. for C_24_H_20_BrN_3_O_6_S_2_ (590.47): C, 48.82; H, 3.41; N, 7.12; S, 10.86. Found: C, 49.11; H, 3.49; N, 7.40; S, 10.92%.

*(Z)-2-(5-(3,4,5-Trimethoxybenzylidene)thiazolidin-2,4-dion-3-yl)-N-(benzo[d]thiazol-2-yl)acetamide (**7f**).*

Yellow crystals, yield 0.426 g (88%), mp 143–144 °C. **IR** (KBr, cm^−1^): 3423 (*ν* N-H), (*ν* C-H arom.), 2932 (*ν* C-H aliph.), 1743 and 1690 (*ν* C=O). **^1^H**-**NMR** (400 MHz, DMSO-*d*_6_): δ (ppm) 12.87 (s, 1H, NH), 7.80 (d, 1H, *J* = 7.8 Hz, Ph-H), 7.96 (s, 1H, -CH=),7.78 (d, 1H, *J* = 8.0 Hz, Ph-H), 7.45 (t, 1H, *J* = 7.1 Hz, Ph-H), 7.34 (t, 1H, *J* = 8.0 Hz, Ph-H), 6.99 (s, 2H, Ph-H), 4.71 (s, 2H, CH_2_), 3.86 (s, 6H, 2 -OCH_3_), 3.75 (s, 3H, -OCH_3_), **^13^C**-**NMR** (100 MHz, DMSO-*d*_6_): δ (ppm) 167.5(C), 165.9 (C=O), 165.6 (C=O), 157.8 (C=O), 153.7 (2 C), 148.9 (C), 140.2 (CH), 134.5 (C), 131.9 (C), 128.7 (C), 126.7 (CH), 124.3 (CH), 122.3 (CH), 121.2 (CH), 120.3 (C), 108.2 (2 CH), 60.4 (OCH_3_), 56.5 (2 OCH_3_), 44.0 (CH_2_). Anal. calcd. for C_22_H_19_N_3_O_6_S_2_ (485.53): C, 54.42; H, 3.94; N, 8.65; S, 13.21. Found: C, 54.79; H, 4.13; N, 8.93; S, 13.38%.

### 4.2. Docking Studies

To build the three-dimensional atomic structures of the molecular targets for our compounds, we used as templates, as previously described [73], the crystal structures of the human Topoisomerase I in covalent and noncovalent complexes with DNA (PDB code 1A35) [74] and the structure of Topoisomerase IIα in complex with a short DNA fragment and etoposide (PDB Code 5gwk) [75] (hTopo I and hTopo II). The atomic coordinates of compounds **7a** to **7f** were built and energy-minimized using the software MarvinSketch [ChemAxon Ltd., Budapest, Hungary]. We performed all of the simulations using the Autodock v.4.2.2. [76] program suite in order to identify the possible poses and assess the binding modes and binding energies of the different compounds to these proteins. We adopted a “blind docking” simulation approach in which the docking of the small molecules to the targets was performed without *a priori* knowledge of the position of the binding site by the system. The simulations were conducted using the standard default values. Proteins and ligands were prepared using the ADT graphical interface [77]. For each protein, we added polar hydrogens, assigned Kollman charges and calculated solvation parameters. Each protein was considered as a rigid object, and all ligands as totally flexible. We built a searching grid and extended it all over the protein, and finally, we calculated affinity maps. The search was conducted with a Lamarckian genetic algorithm: a population of 100 individuals with a mutation rate of 0.02 was evolved for 100 generations. Amino acidic residues involved in binding are labeled and reported in Figure 1 and Figure 2. Evaluation of the results was performed by listing the different ligand poses according to their predicted binding energies. A cluster analysis was conducted on the basis of the root mean squares deviation (RMSD) values from the starting geometry. The lowest energetic conformation of the most populated cluster was considered as the best candidate. Clusters almost equipopulated and with a spread energy distribution indicated molecules considered as bad ligands [78]. 

The obtained docking poses were ranked in terms of increasing binding energy values and clustered using an RMSD cut-off value of 2.0 Å. From the structural analysis of the lowest energy solutions of each cluster, the protein binding site could be spotted. Figures were drawn using the Chimera software [79]. Two-dimensional plots were calculated using LIGPLOT [53]

### 4.3. Cell Cultures

The used cell lines (breast cancer cell lines MCF-7 and MDA-MB-231, melanoma cell line A2058 and human mammary epithelial cell line MCF-10A) were obtained from American Type Culture Collection (ATCC, Manassas, VA, USA) and cultured as already reported [73]. The mouse BALB/3T3 embryonic fibroblasts were also purchased from American Type Culture Collection (ATCC, Manassas, VA, USA) and cultured in DMEM high glucose supplemented with 10% bovine calf serum (BCS) and 100 U mL^−1^ penicillin/streptomycin.

### 4.4. Biology

#### 4.4.1. MTT Assay

MTT assay (Sigma Aldrich (St. Louis, MO, USA)) was performed for evaluating the in vitro anticancer activities of the compounds, as previously indicated [80,81]. Different concentrations (0.1, 1, 10, 25, 50, and 100 μM) of the compounds were employed, with a 72 h endpoint. IC_50_ values were obtained from the percent (%) of control using GraphPad Prism 9 (GraphPad Software, La Jolla, CA, USA).

#### 4.4.2. hTopo I Relaxation Assay and hTopo II Decatenation Assay

hTopo I relaxation assays were performed by incubating the substrate (supercoiled pHOT1) with the recombinant hTopo I (TopoGEN, Port Orange, FL, USA) and compounds, as indicated in the supplier protocol (TopoGEN, Port Orange, FL, USA), with some revisions [80]. hTopo II decatenation assays were conducted by incubating the kinetoplast DNA substrate (kDNA) with the hTopo II (TopoGEN, Port Orange, FL) and studied compounds, as indicated in the supplier procedures (TopoGEN, Port Orange, FL, USA), with some changes as in [80].

#### 4.4.3. TUNEL Assay

Cell apoptosis was detected by means of the TUNEL assay, following the manufacturer’s protocols (CF™488A TUNEL Assay Apoptosis Detection Kit, Biotium, Hayward, CA, USA), with few modifications, as already reported [60]. DAPI staining (0.2 μg/mL, Sigma Aldrich, Milan, Italy) was used for cell nuclei. A fluorescence microscope (Leica DM 6000) was used for the observations at 20× magnification. LAS-X software allowed the acquisition and processing of all of the images, which were representative of three separate experiments.

#### 4.4.4. Immunofluorescence Assays

Cells grown on glass coverslips in full medium were serum-deprived for 24 h, then the compound (or only vehicle) was added (24 h, **7e** at its IC_50_ value). Next, cells were incubated with a staining solution containing MitoTracker^®^ Deep Red FM probe (MitoTracker^®^ Mitochondrion-Selective Probes, Invitrogen European Headquarters, Paisley PA4 9RF, UK; fluorescence excitation = 644, fluorescence emission = 665) for 20 min, at 37 °C. After a PBS wash, cells were fixed (cold methanol, 15 min at −20 °C) and incubated with the mouse anti-cytochrome c (556 433) primary antibody, purchased from BD, diluted in the blocking solution (4 °C/overnight), as described in [60]. Alexa Fluor^®^ 488 conjugate goat-anti-mouse (1:500 dilution), purchased from Thermo Fisher Scientific, MA, USA, was used as secondary antibody. DAPI (Sigma Aldrich, Mila, Italy), for nuclei counter-stain, was added (10 min, concentration of 0.2 μg/mL), then the mixture was washed thrice with PBS. A fluorescence microscope (Leica DM 6000) was used for visualizing images, and LAS-X software for acquisition and processing.

#### 4.4.5. Caspase Assay

The caspase-Glo Assay (Caspase-Glo^®^ 3/7, 8 and 9 Assay Systems, Promega Corporation, Madison, WI) was used to detect caspase-3/7, -8 and -9 activities, as reported in [82]. Briefly, cells were grown in white-walled 96-well plates, treated with the compound, and 100 µL of each caspase was added.

#### 4.4.6. Statistical Analysis

One-way ANOVA followed by Dunnett’s test was performed by GraphPad Prism 9. Data were adopted for analysis of statistical significance (*p* < 0.001). Standard deviations (SD) are shown. 

## 5. Conclusions

In this paper, we demonstrated the anticancer activity of new hybrid molecules containing three pharmacophores, namely trimethoxybenzene, thiazolidinedione and thiazole. The most active compound targets were hTopo I and II, the blockade of which induced the intrinsic apoptotic mechanism. Our data highlight the importance of the hybrid drug design and shed light on the intracellular mechanisms underlying the biological activity.

## Figures and Tables

**Table 1:** IC_50_ values, expressed in µM, of the studied compounds **7a**–**f** and colchicine and ellipticine against different cell lines.

IC_50_ (µM)					
Compounds	MDA-MB-231	MCF-7	A2058	3T3 BALB	MCF-10A
**7a**	27.5 ± 0.9	>100	>100	89.2 ± 1.1	>100
**7b**	17.9 ± 1.1	8.5 ± 1.2	3.5 ± 1.1	>100	>100
**7c**	32.8 ± 0.6	19.5 ± 0.6	4.8 ± 1.0	>100	46.4 ± 1.1
**7d**	33.4 ± 1.0	34.1 ± 0.7	6.4 ± 0.9	36.1 ± 1.0	>100
**7e**	41.2 ± 0.9	3.1 ± 0.6	8.4 ± 0.9	35.0 ± 0.6	>100
**7f**	50.5 ± 0.8	17.4 ± 1.1	24.6 ± 1.1	>100	>100
**colchicine**	8.1 (± 1.0) × 10^−2^	1.6 (± 0.8) × 10^−2^	3.4 (± 0.7) × 10^−2^	5.2 (± 0.9) × 10^−1^	8.9 (± 1.2) × 10^−3^
**ellipticine**	1.73 ± 0.4	1.15 ± 0.5	1.33 ± 0.9	0.97 ± 0.1	1.09 ± 0.1

**Table 2:** Overview of the hTopo assays: (+) inhibition; (−) no inhibition; (+/−) partial inhibition.

Topoisomerase Inhibition			
Compounds	[µM]	hTopo I	hTopo II
**7b**	10	−	−
	50		−
**7c**	10	−	−
	50		+
**7d**	10	−	−
	50		+
**7e**	10	−	−
	50		+
**7f**	10	+	−
	50		+
**ellipticine**	10	+	+/−
	50	+	+

The color indicates the degree of inhibition (null = orange, intermediate = yellow, total = green).

## Data Availability

Data are contained within the article and Supplementary Materials.

## Supplementary Materials

The following supporting information can be downloaded at: https://www.mdpi.com/article/10.3390/ph16070946/s1, Figures S1–S25, IR spectra; Figures S26–S58 ^1^H- and ^13^C-NMR spectra. Figures S59–S64 2D diagrams for hTopo I; Figures S65–S70 2D diagrams for hTopo II.
