# Supplementary material for: Novel Thiazolidine-2,4-dione-trimethoxybenzene-thiazole Hybrids as Human Topoisomerases Inhibitors
Source: Pharmaceuticals (Basel). 2023 Jun 29;16(7):946. doi: 10.3390/ph16070946 (PMC10384675; doi:10.3390/ph16070946)
Supplement: Supplementary file 1 [file pharmaceuticals-16-00946-s001.zip › pharmaceuticals-2393787-supplementary.pdf]

Supplementary materials

Novel Thiazolidine-2,4-Dione-Trimethoxybenzene-Thiazole Hybrids as Human Topoisomerases Inhibitors

Maria Stefania Sinicropi[1,†], Jessica Ceramella[1,†], Patrice Vanelle[2], Domenico Iacopetta[1]*, Camillo Rosano[3], Omar Khoumeri[2], Shawkat Abdelmohsen[4], Wafaa Abdelhady[4] and Hussein El-Kashef[4,5]*

[1] Department of Pharmacy, Health and Nutritional Sciences, University of Calabria, 87036 Arcavacata di Rende, Italy. s.sinicropi@unical.it (M.S.S.); jessica.ceramella@unical.it (J.C.).

[2] Aix Marseille University, CNRS, ICR UMR 7273, Equipe Pharmaco-Chimie Radicalaire, Faculté de Pharmacie, 27 Boulevard Jean Moulin, CS30064, 13385, Marseille Cedex 05, France. patrice.vanelle@univ-amu.fr (P.V.); omar.khoumeri@univ-amu.fr (O.K.).

[3] U.O. Proteomica e Spettrometria di Massa, IRCCS Ospedale Policlinico San Martino, Largo R. Benzi 10, 16132 Genova, Italy. camillo.rosano@hsanmartino.it (C.R.)

[4] Department of Chemistry, Faculty of Science, Assiut University, 71516 Assiut, Egypt. shawk662001@yahoo.com (S.A.); wafaahadi13@gmail.com (W.A.).

[5] Faculty of Pharmacy, Sphinx University, 71684 New Assiut, Egypt.

† These authors equally contributed.

* Correspondence: domenico.iacopetta@unical.it (D.I.), Tel. +39-0984-493200; elkashef@aun.edu.eg (H.E.K.), Tel. +20 1005075881

Contents

IR spectra, ^{1}H- and ^{13}C-NMR spectra

1) IR spectra

Fig. S1. IR spectrum of 1,3-thiazolidine-2,4-dione (**1**).

Fig. S2. IR spectrum of (*Z*)-5-(3,4,5-Trimethoxybenzylidene)thiazolidine-2,4-dione (**2**).

Fig. S3. IR spectrum of potassium (Z)-5-(3,4,5-Trimethoxybenzylidene)thiazolidine-2,4-dion-3-ide (**3**).

Fig. S4. IR spectrum of 2-amino-4-phenylthiazole (**4b**).

Fig. S5. IR spectrum of 2-amino-4-(4-methylphenyl)thiazole (**4c**).

Fig. S6. IR spectrum of 2-amino-4-(4-chlorolphenyl)thiazole (**4d**).

Fig. S7. IR spectrum of 2-amino-4-(4-bromolphenyl)thiazole (**4e**).

Fig. S8. IR spectrum of 2-chloro-N-(thiazol-2-yl)acetamide (**5a**).

Fig. S9. IR spectrum of 2-chloro-N-(4-phenylthiazol-2-yl)acetamide (**5b**).

Fig. S10. IR spectrum of 2-chloro-N-(4-methylphenylthiazol-2-yl)acetamide (**5c**).

Fig. S11. IR spectrum of 2-chloro-N-(4-chlorophenylthiazol-2-yl)acetamide (**5d**).

Fig. S12. IR spectrum of 2-chloro-N-(4-bromophenylthiazol-2-yl)acetamide (**5e**).

Fig. S13. IR spectrum of 2-chloro-N-(benzo[*d*] thiazol-2-yl)acetamide (**5f**).

Fig. S14. IR spectrum of 2-iodo-N-(thiazol-2-yl)acetamide (**6a**).

Fig. S15. IR spectrum of 2-iodo-N-(4-phenylthiazol-2-yl)acetamide (**6b**).

Fig. S16. IR spectrum of 2-iodo-N-(4-methyl phenylthiazol-2-yl)acetamide (**6c**).

Fig. S17. IR spectrum of 2-iodo -N-(4-chlorophenylthiazol-2-yl)acetamide (**6d**).

Fig. S18. IR spectrum of 2-iodo -N-(4-bromophenylthiazol-2-yl)acetamide (**6e**).

Fig. S19. IR spectrum of 2-iodo-N-(benzo[*d*]thiazol-2-yl)acetamide (**6f**).

Fig. S20. IR spectrum of (Z)-2-(5-(3,4,5-trimethoxybenzylidene)thiazolidin-2,4-dion-3-yl)-N-(thiazol-2-yl)acetamide (**7a**).

Fig. S21. IR spectrum of (*Z*)-2-(5-(3,4,5-trimethoxybenzlidene)thiazolidine-2,4-dion-3-yl)-N-(4-phenylthiazole-2-yl)acetamide (**7b**).

Fig. S22. IR spectrum of (Z)-2-(5-(3,4,5-trimethoxybenzylidene)thiazolidin-2,4-dion-3-yl)-N-(4-(4-methylphenyl)thiazol-2-yl)acetamide (**7c**).

Fig. S23. IR spectrum of (*Z*)-2-(5-(3,4,5-trimethoxybenzylidene)thiazolidin-2,4-dion-3-yl)-N-(4-(4-chlorophenyl)thiazol-2-yl)acetamide (**7d**).

Fig. S24. IR spectrum of (*Z*)-2-(5-(3,4,5-trimethoxybenzylidene)thiazolidin-2,4-dion-3-yl)-N-(4-(4-bromophenyl)thiazol-2-yl)acetamide (**7e**).

Fig. S25. IR spectrum of (*Z*)-2-(5-(3,4,5-trimethoxybenzylidene)thiazolidin-2,4-dion-3-yl)-N-(benzo[*d*]thiazol-2-yl)acetamide (**7e**).

2) ^{1}H- and ^{13}C-NMR spectra in DMSO-d_6 if not otherwise indicated

Fig. S26. ^{1}H-NMR spectrum of (*Z*)-5-(3,4,5-Trimethoxybenzylidene) thiazolidine-2,4-dione **(2).**

Fig. S27. ^{13}C-NMR spectrum of (*Z*)-5-(3,4,5-Trimethoxybenzylidene)thiazolidine-2,4-dione **(2).**

Fig. S28. ^{1}H-NMR spectrum of potassium (Z)-5-(3,4,5-Trimethoxybenzylidene)thiazolidine-2,4-dion-3-ide (**3**).

Fig. S29. ^{13}C-NMR spectrum of potassium (Z)-5-(3,4,5-Trimethoxybenzylidene)thiazolidine-2,4-dion-3-ide (**3**).

Fig. S30. ^{1}H-NMR spectrum ($CDCl_3$) of 2-chloro-N-(thiazol-2-yl)acetamide (**5a**).

Fig. S31. ^{1}H-NMR spectrum ($CDCl_3$) of 2-chloro-N-(4-phenylthiazol-2-yl)acetamide (**5b**).

Fig. S32. ^{1}H-NMR spectrum ($CDCl_3$) of 2-chloro-N-(4-methylphenylthiazol-2-yl)acetamide (**5c**).

Fig. S33. ^{1}H-NMR spectrum ($CDCl_3$) of 2-chloro-N-(4-chlorophenylthiazol-2-yl)acetamide (**5d**).

Fig. S34. ^{1}H-NMR spectrum ($CDCl_3$) of 2-chloro -N-(4-bromophenylthiazol-2-yl)acetamide (**5e**).

Fig. S35. ^{1}H-NMR spectrum ($CDCl_3$) of 2-chloro-N-(benzo[*d*]thiazol-2-yl)acetamide (**5f**).

Fig. S36. ^{1}H-NMR spectrum of 2-iodo-N-(thiazol-2-yl)acetamide (**6a**).

Fig. S37. ^{13}C-NMR spectrum ($CDCl_3$) of 2-iodo-N-(thiazol-2-yl)acetamide (**6a**).

Fig. S38. ^{1}H-NMR spectrum of 2-iodo-N-(4-phenylthiazol-2-yl)acetamide (**6b**).

Fig. S39. ^{13}C-NMR spectrum of 2-iodo-N-(4-phenylthiazol-2-yl)acetamide (**6b**).

Fig. S40. ^{1}H-NMR spectrum of 2-iodo-N-(4-methylphenylthiazol-2-yl)acetamide (**6c**).

Fig. S41. ^{13}C-NMR spectrum of 2-iodo-N-(4-methyl phenylthiazol-2-yl)acetamide (**6c**).

Fig. S42. ^{1}H-NMR spectrum of 2-iodo-N-(4-chlorophenylthiazol-2-yl)acetamide (**6d**).

Fig. S43. ^{13}C-NMR spectrum of 2-iodo-N-(4-chlorophenylthiazol-2-yl)acetamide (**6d**).

Fig. S44. ^{1}H-NMR spectrum of 2-iodo -N-(4-bromophenylthiazol-2-yl)acetamide (**6e**).

Fig. S45. ^{13}C-NMR spectrum of 2-iodo -N-(4-bromophenylthiazol-2-yl)acetamide (**6e**).

Fig. S46. ^{1}H-NMR spectrum of 2-iodo-N-(benzo[*d*]thiazol-2-yl)acetamide (**6f**).

Fig. S47. ^{13}C-NMR spectrum of 2-iodo-N-(benzo[*d*]thiazol-2-yl)acetamide (**6f**).

Fig. S48. ^{1}H-NMR spectrum of (*Z*)-2-(5-(3,4,5-trimethoxybenzlidene)thiazolidine-2,4-dion-3-yl)-N-(thiazole-2-yl)acetamide (**7a**).

Fig. S49. ^{13}C-NMR spectrum of (*Z*)-2-(5-(3,4,5-trimethoxybenzlidene)thiazolidine-2,4-dion-3-yl)-N-(thiazole-2-yl)acetamide (**7a**).

Fig. S50. ^{1}H-NMR spectrum of (*Z*)-2-(5-(3,4,5-trimethoxybenzlidene)thiazolidine-2,4-dion-3-yl)-N-(4-phenylthiazole-2-yl)acetamide (**7b**).

Fig. S51. ^{13}C-NMR spectrum of (*Z*)-2-(5-(3,4,5-trimethoxybenzlidene)thiazolidine-2,4-dion-3-yl)-N-(4-phenylthiazole-2-yl)acetamide (**7b**).

Fig. S52. ^{1}H-NMR spectrum of (Z)-2-(5-(3,4,5-trimethoxybenzylidene)thiazolidin-2,4-dion-3-yl)-N-(4-(4-methylphenyl)thiazol-2-yl)acetamide (**7c**).

Fig. S53. ^{13}C-NMR spectrum of (Z)-2-(5-(3,4,5-trimethoxybenzylidene)thiazolidin-2,4-dion-3-yl)-N-(4-(4-methylphenyl)thiazol-2-yl)acetamide (**7c**).

Fig. S54. ^{1}H-NMR spectrum of (*Z*)-2-(5-(3,4,5-trimethoxybenzylidene)thiazolidin-2,4-dion-3-yl)-N-(4-(4-chlorophenyl)thiazol-2-yl)acetamide (**7d**).

Fig. S55. ^{13}C-NMR spectrum of (*Z*)-2-(5-(3,4,5-trimethoxybenzylidene)thiazolidin-2,4-dion-3-yl)-N-(4-(4-chlorophenyl)thiazol-2-yl)acetamide (**7d**).

Fig. S56. ^{1}H-NMR spectrum of (*Z*)-2-(5-(3,4,5-trimethoxybenzylidene)thiazolidin-2,4-dion-3-yl)-N-(4-(4-bromophenyl)thiazol-2-yl)acetamide **(7e)**.

Fig. S57. ^{13}C-NMR spectrum of (*Z*)-2-(5-(3,4,5-trimethoxybenzylidene)thiazolidin-2,4-dion-3-yl)-N-(4-(4-bromophenyl)thiazol-2-yl)acetamide (**7e**).

Fig. S58. ^{1}H-NMR spectrum of (*Z*)-2-(5-(3,4,5-trimethoxybenzylidene)thiazolidin-2,4-dion-3-yl)-N-(benzo[*d*]thiazol-2-yl)acetamide (**7f**).

3) 2D-schematic representation of ligand-protein interactions. All the plots were calculated using LIGPLOT (Wallace A C, Laskowski R A & Thornton J M (1995). LIGPLOT: A program to generate schematic diagrams of protein-ligand interactions. *Prot. Eng.*, 8, 127-134.). Figures S59-S64 are relative to hTopo I; Figures S65-S70 are relative to hTopo II.

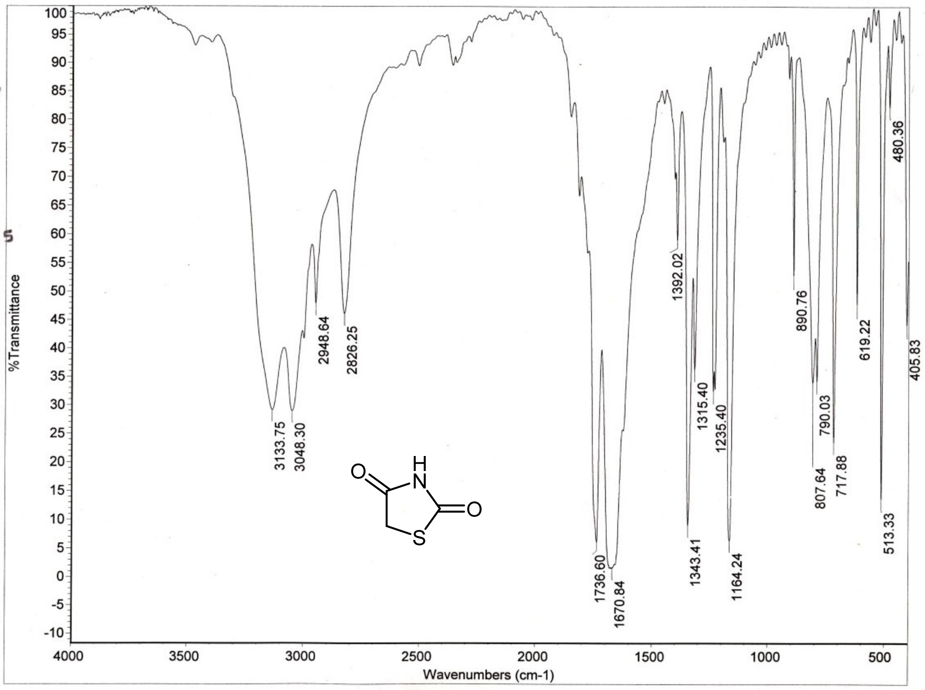

Fig. S1. IR spectrum of 1,3-thiazolidine-2,4-dione (**1**).

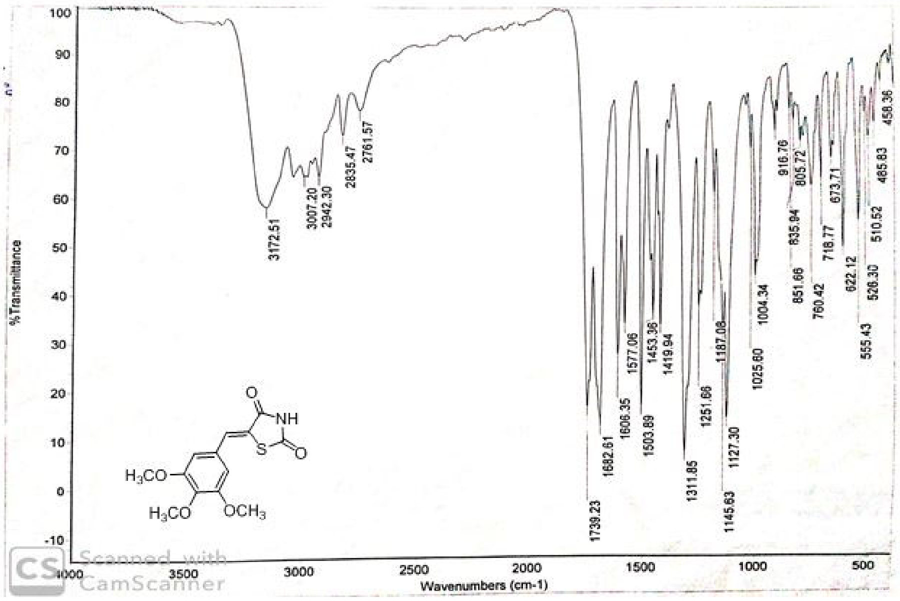

Fig. S2. IR spectrum of (*Z*)-5-(3,4,5-Trimethoxybenzylidene)thiazolidine-2,4-dione (**2**).

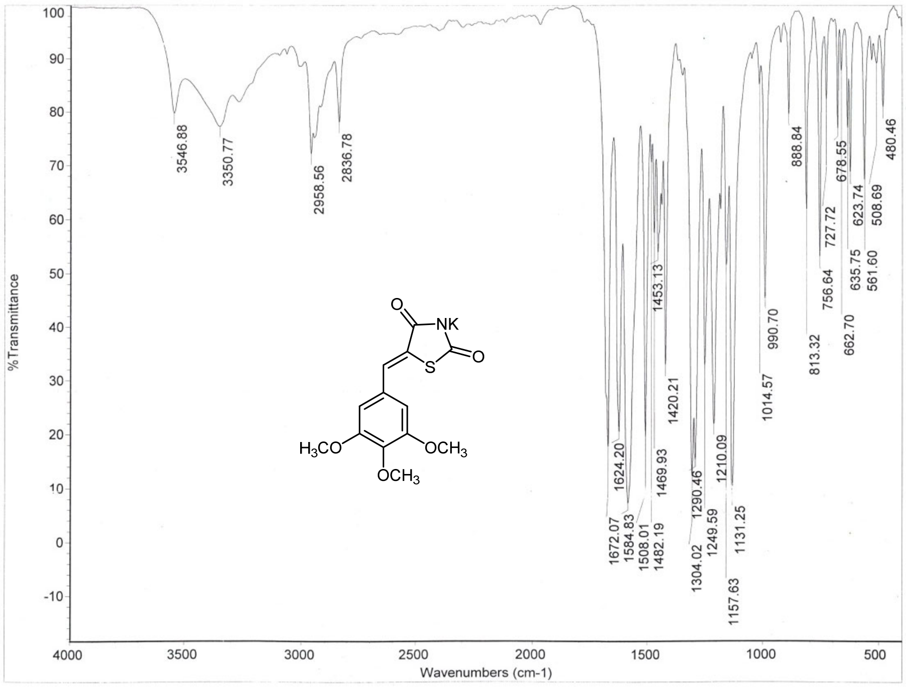

Fig. S3. IR spectrum of potassium (Z)-5-(3,4,5-Trimethoxybenzylidene)thiazolidine-2,4-dion-3-ide (**3**).

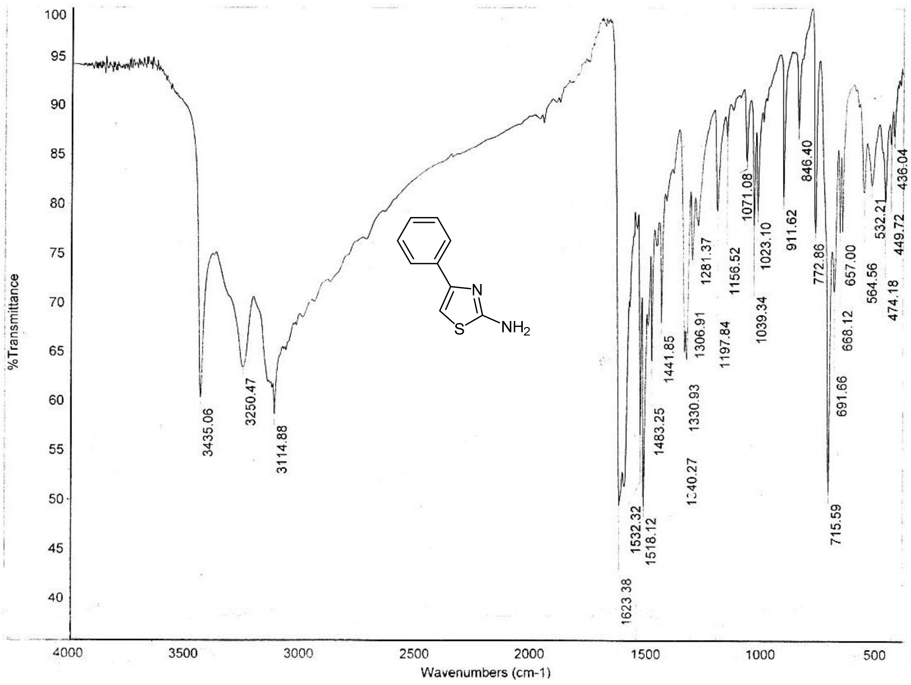

Fig. S4. IR spectrum of 2-amino-4-phenylthiazole (**4b**).

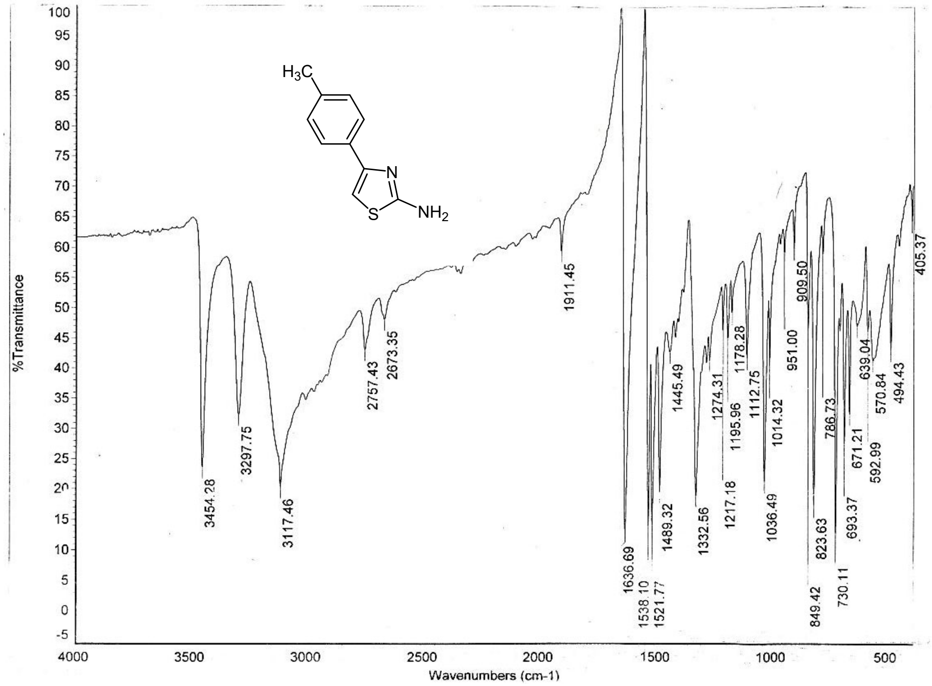

Fig. S5. IR spectrum of 2-amino-4-(4-methylphenyl)thiazole (**4c**).

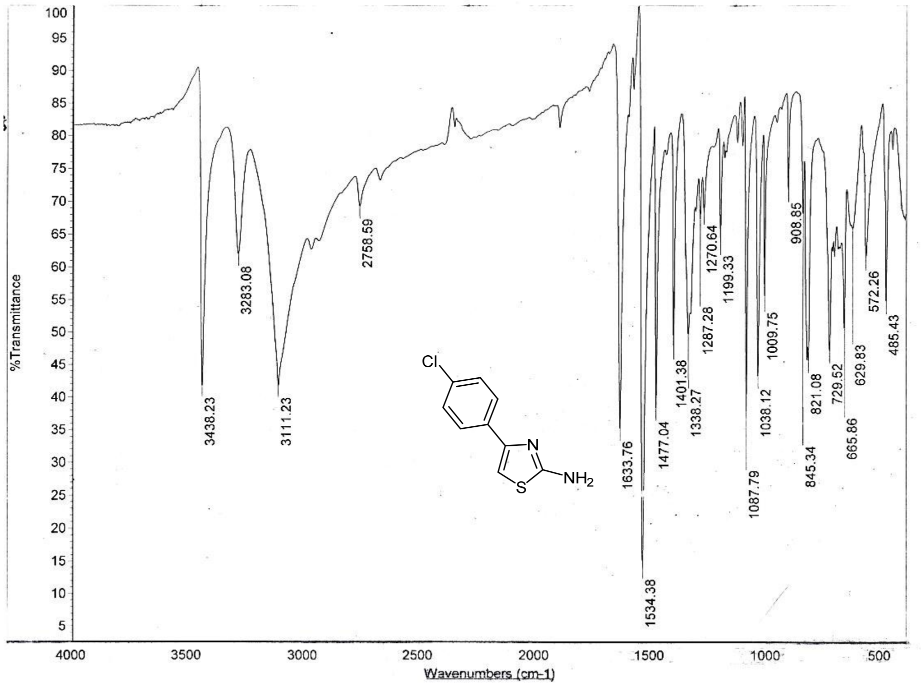

Fig. S6. IR spectrum of 2-amino-4-(4-chlorolphenyl)thiazole (**4d**).

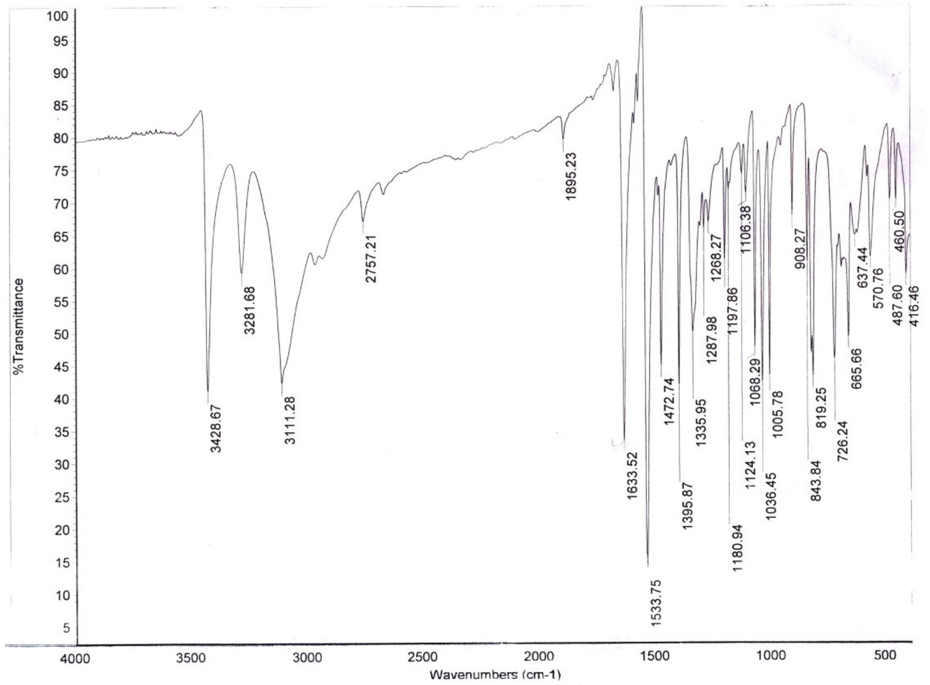

Fig. S7. IR spectrum of 2-amino-4-(4-bromolphenyl)thiazole (**4e**).

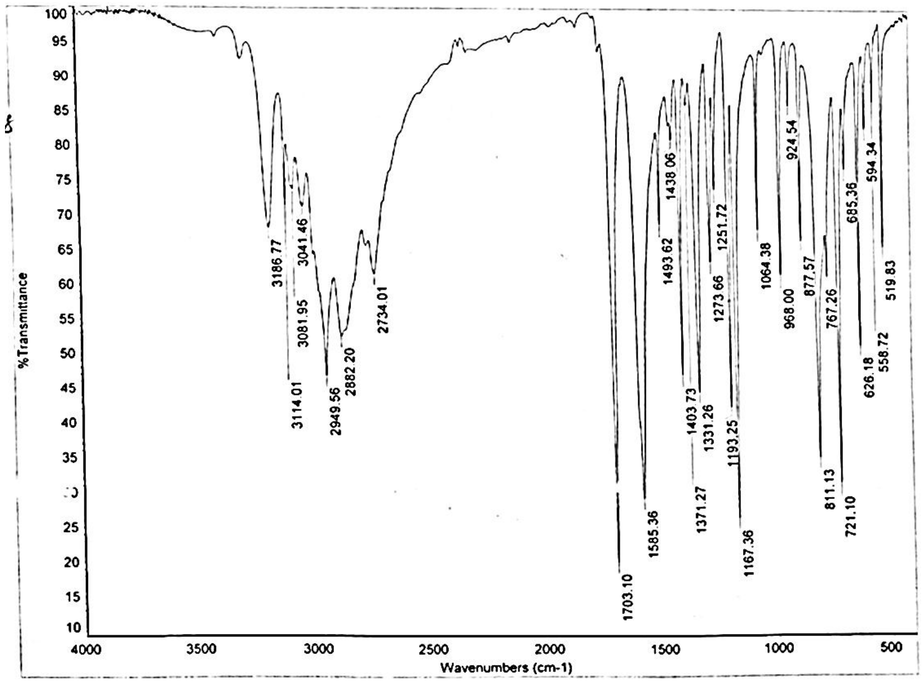

Fig. S8. IR spectrum of 2-chloro-N-(thiazol-2-yl)acetamide (**5a**).

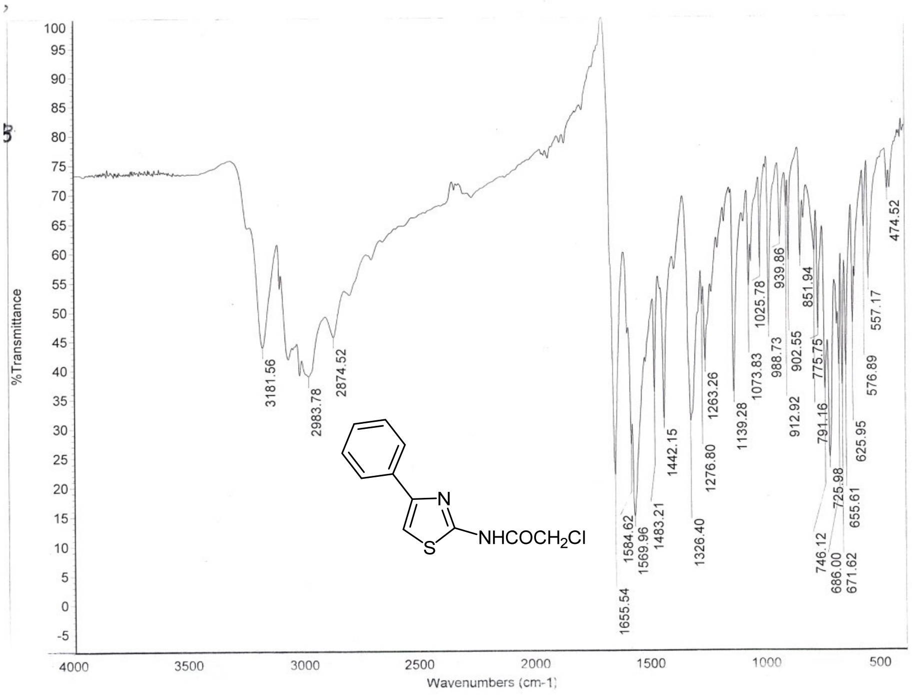

Fig. S9. IR spectrum of 2-chloro-N-(4-phenylthiazol-2-yl)acetamide (**5b**).

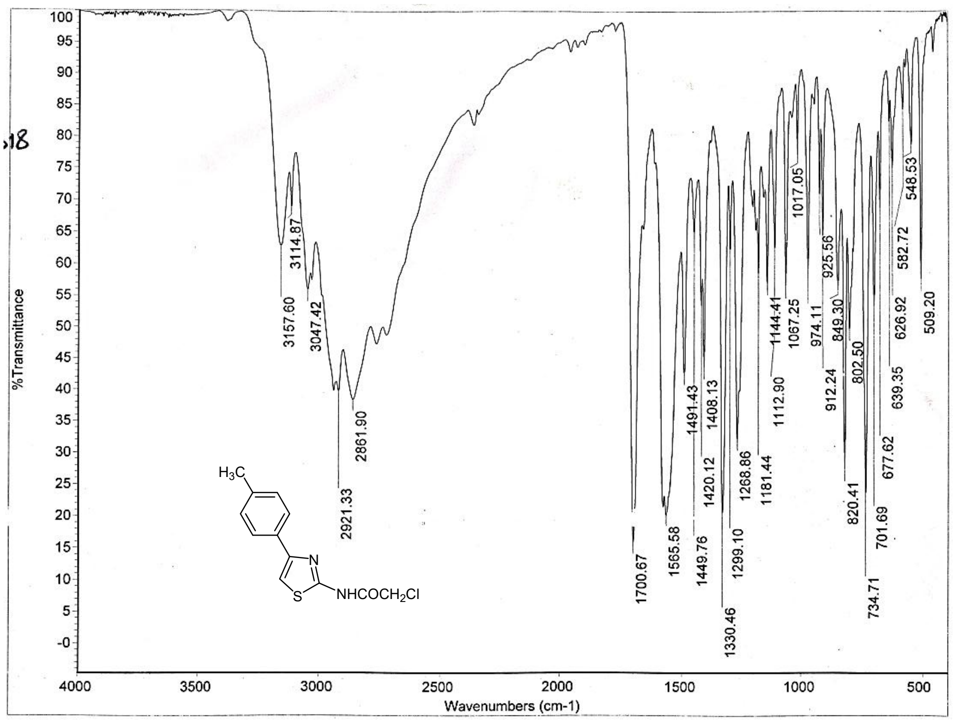

Fig. S10. IR spectrum of 2-chloro-N-(4-methylphenylthiazol-2-yl)acetamide (**5c**).

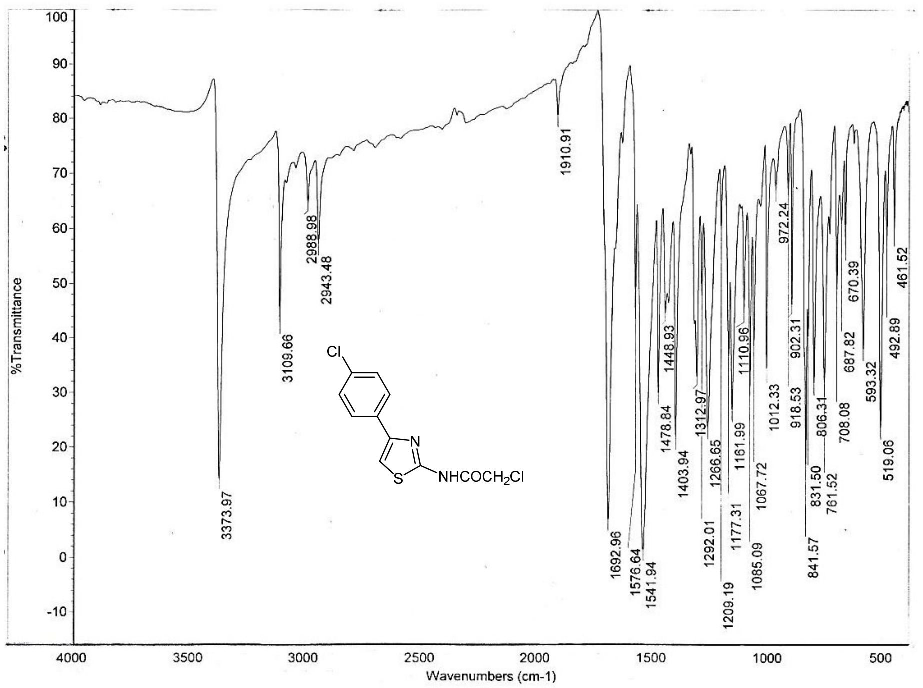

Fig. S11. IR spectrum of 2-chloro-N-(4-chlorophenylthiazol-2-yl)acetamide (**5d**).

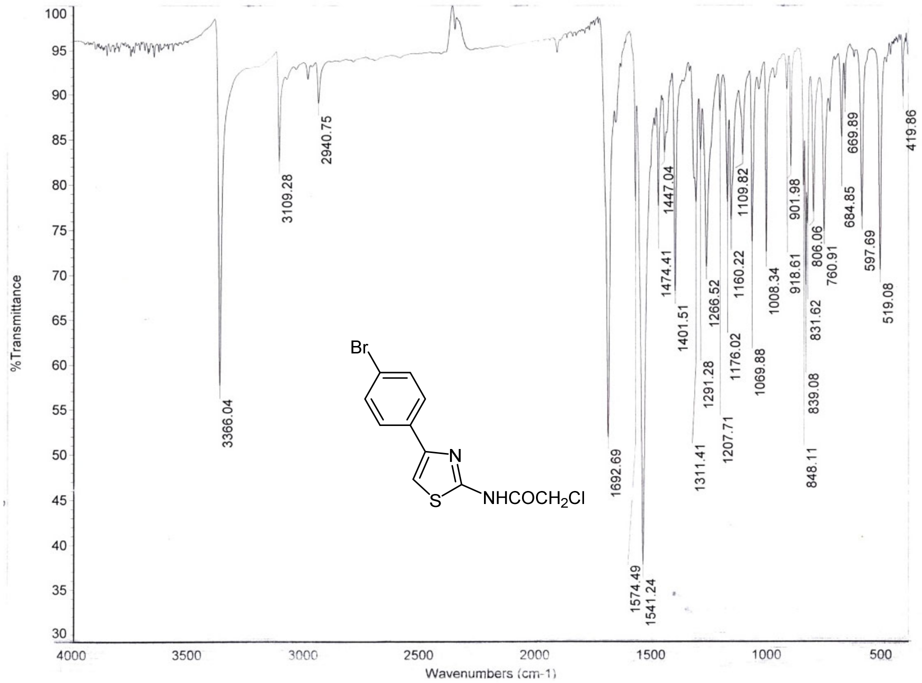

Fig. S12. IR spectrum of 2-chloro-N-(4-bromophenylthiazol-2-yl)acetamide (**5e**).

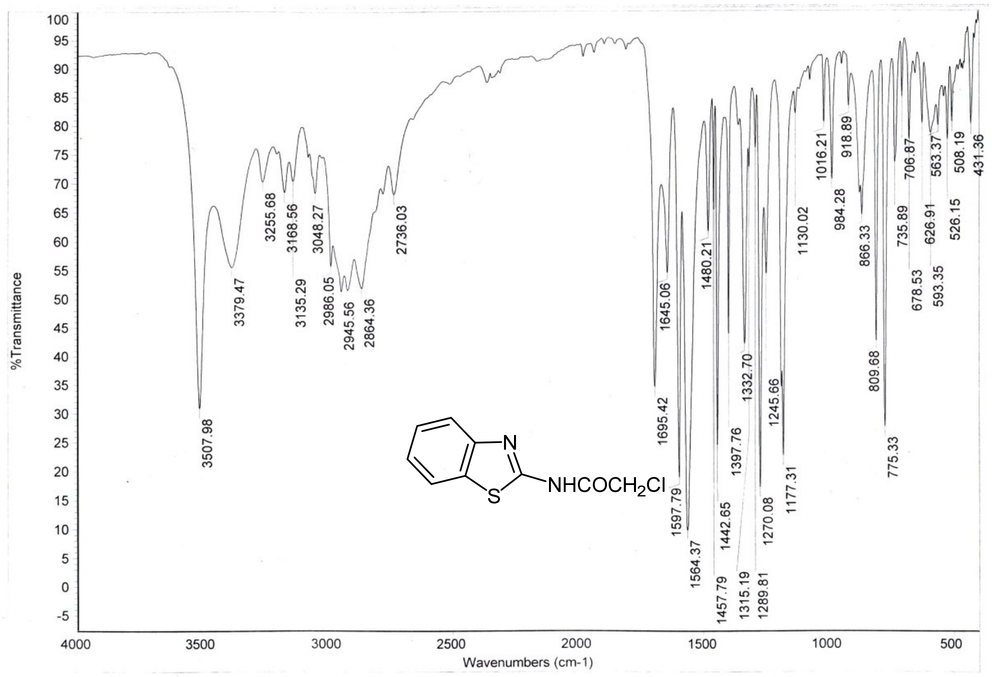

Fig. S13. IR spectrum of 2-chloro-N-(benzo[*d*] thiazol-2-yl)acetamide (**5f**).

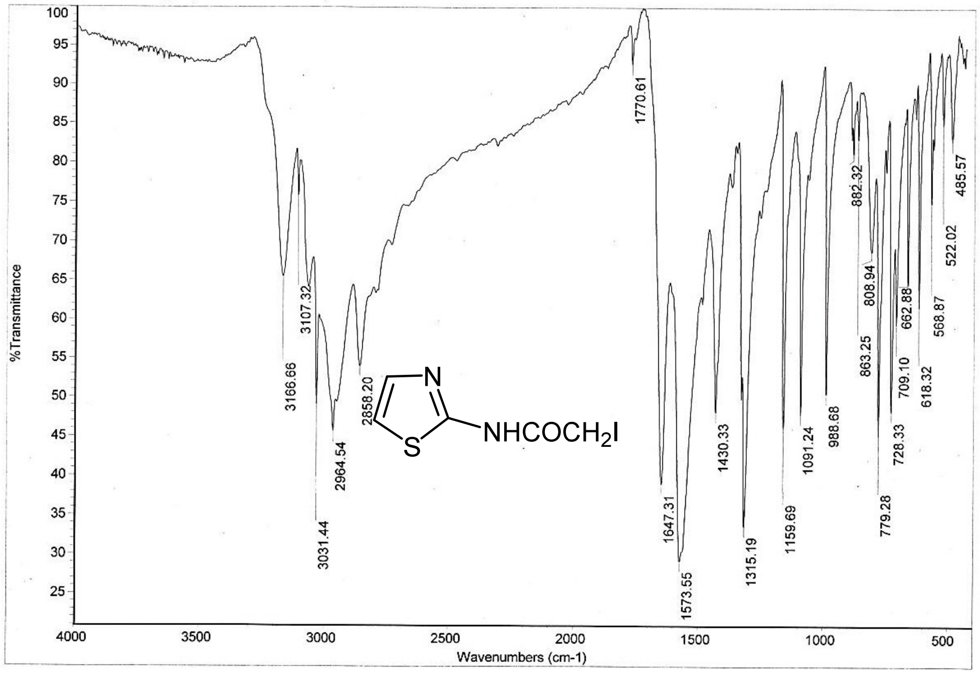

Fig. S14. IR spectrum of 2-iodo-N-(thiazol-2-yl)acetamide (**6a**).

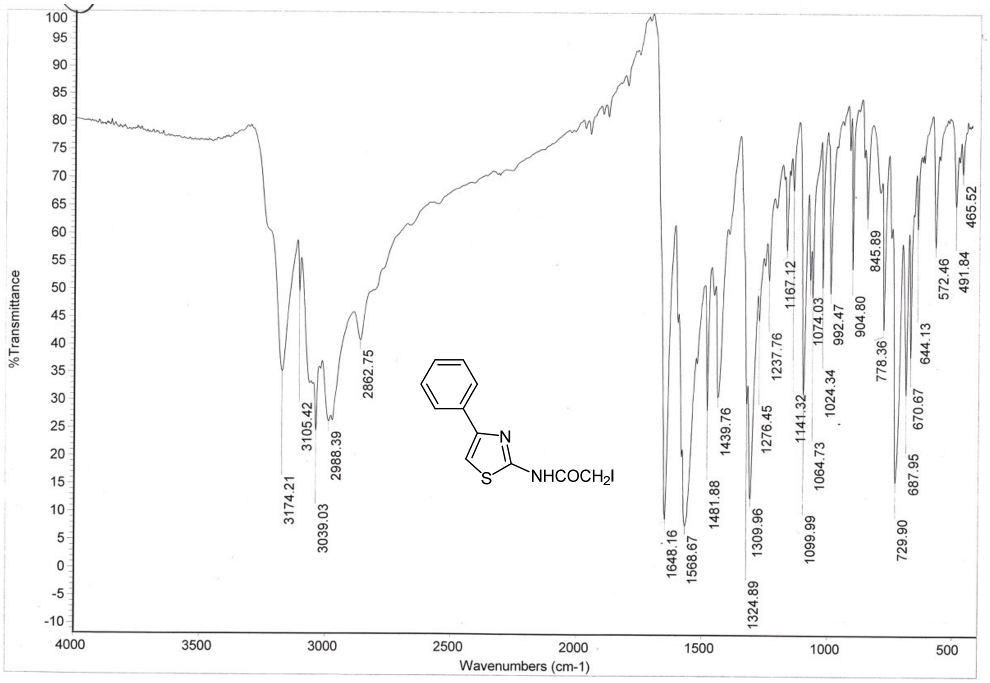

Fig. S15. IR spectrum of 2-iodo-N-(4-phenylthiazol-2-yl)acetamide (**6b**).

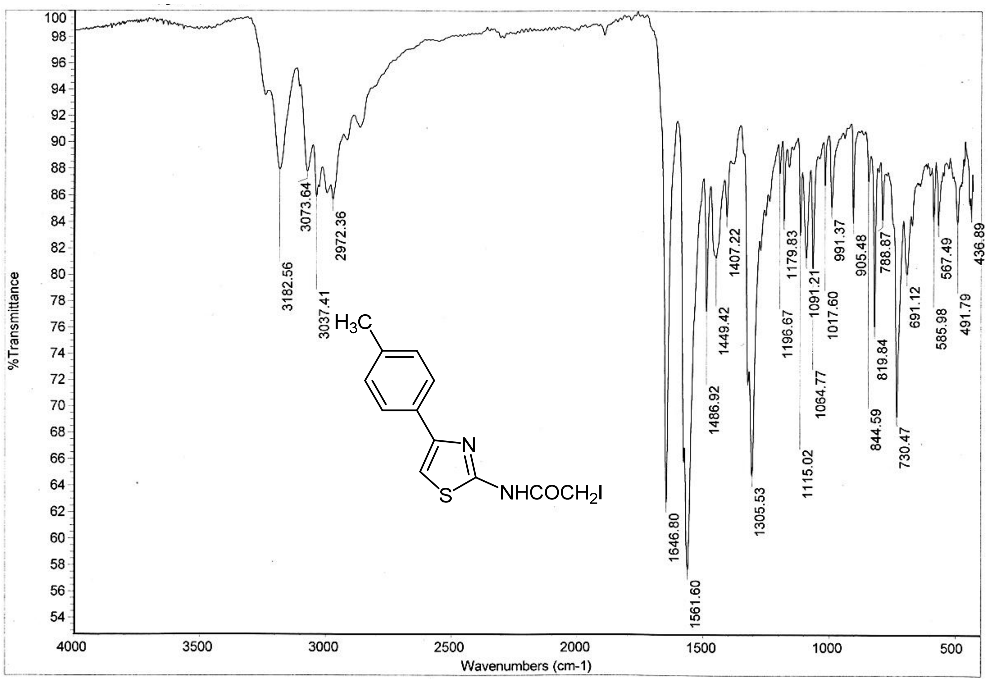

Fig. S16. IR spectrum of 2-iodo-N-(4-methyl phenylthiazol-2-yl)acetamide (**6c**).

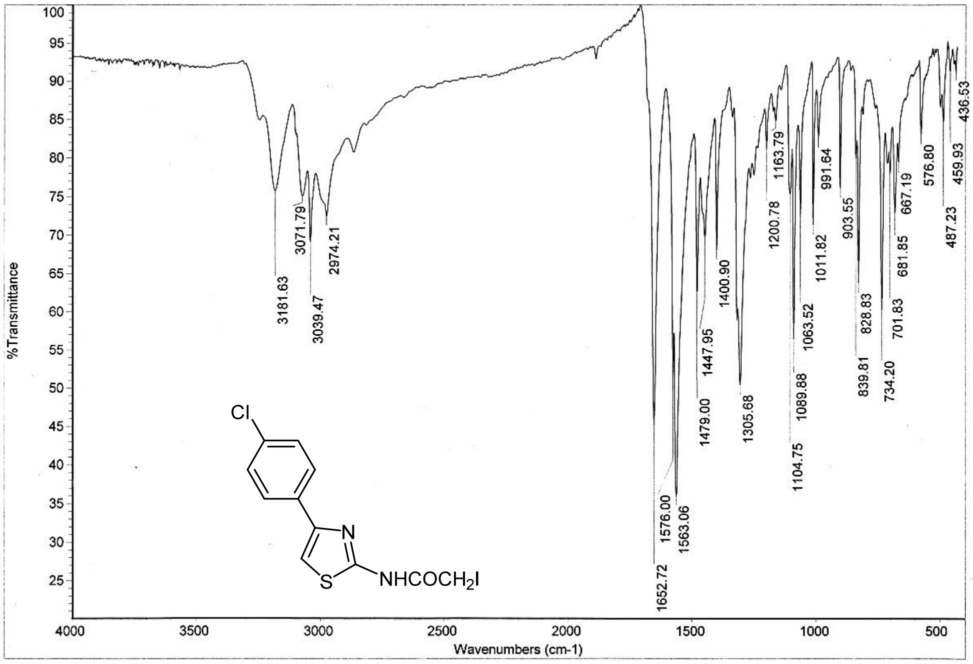

Fig. S17. IR spectrum of 2-iodo -N-(4-chlorophenylthiazol-2-yl)acetamide (**6d**).

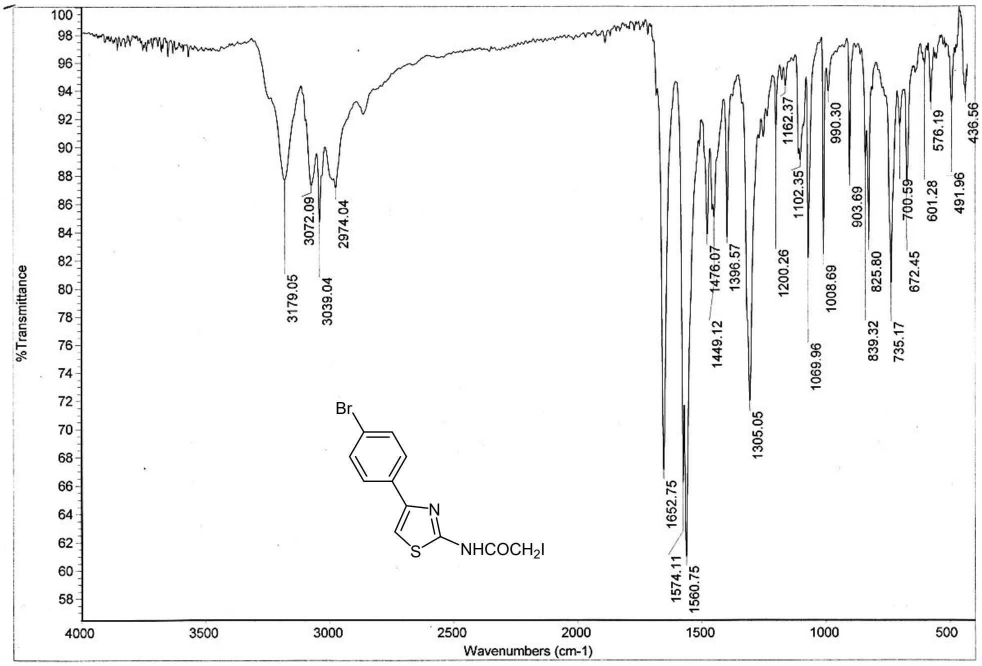

Fig. S18. IR spectrum of 2-iodo -N-(4-bromophenylthiazol-2-yl)acetamide (**6e**).

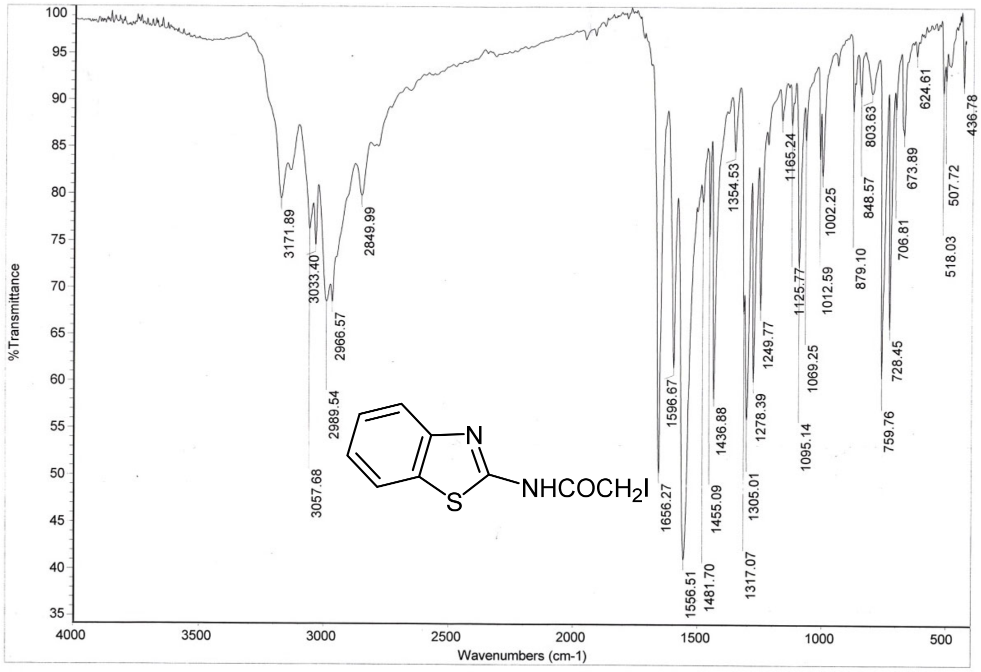

Fig. S19. IR spectrum of 2-iodo-N-(benzo[*d*]thiazol-2-yl)acetamide (**6f**).

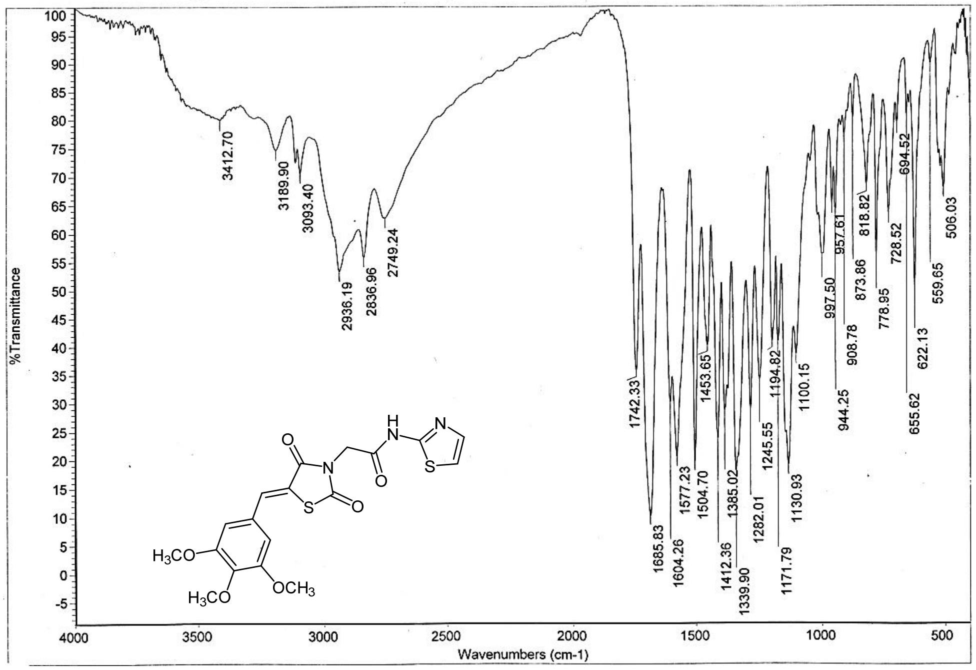

Fig. S20. IR spectrum of (Z)-2-(5-(3,4,5-trimethoxybenzylidene)thiazolidin-2,4-dion-3-yl)-N-(thiazol-2-yl)acetamide (**7a**).

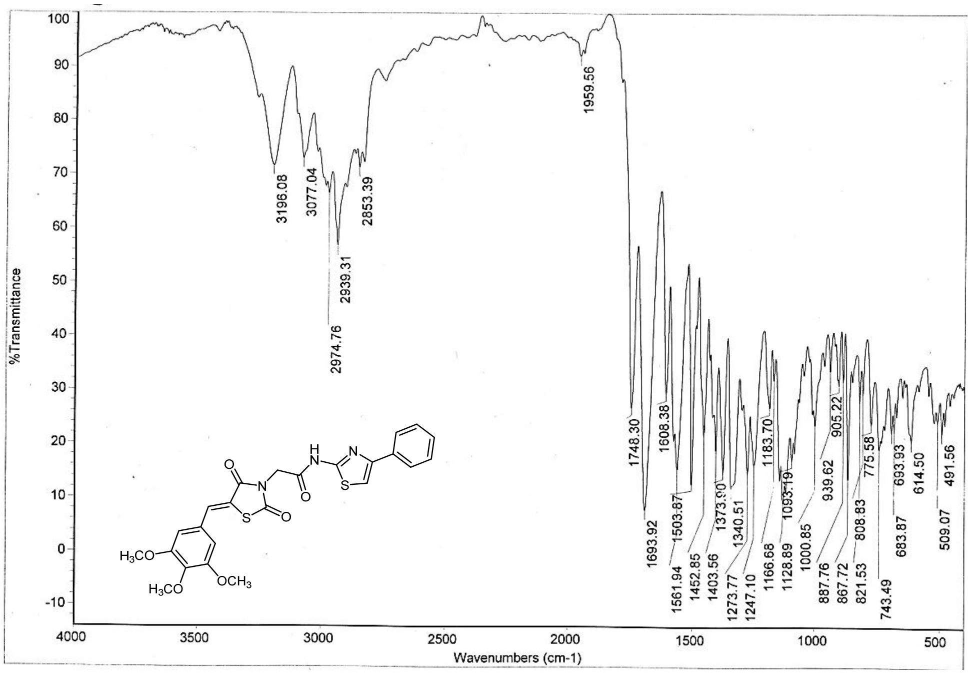

Fig. S21. IR spectrum of (*Z*)-2-(5-(3,4,5-trimethoxybenzlidene)thiazolidine-2,4-dion-3-yl)-N-(4-phenylthiazole-2-yl)acetamide (**7b**).

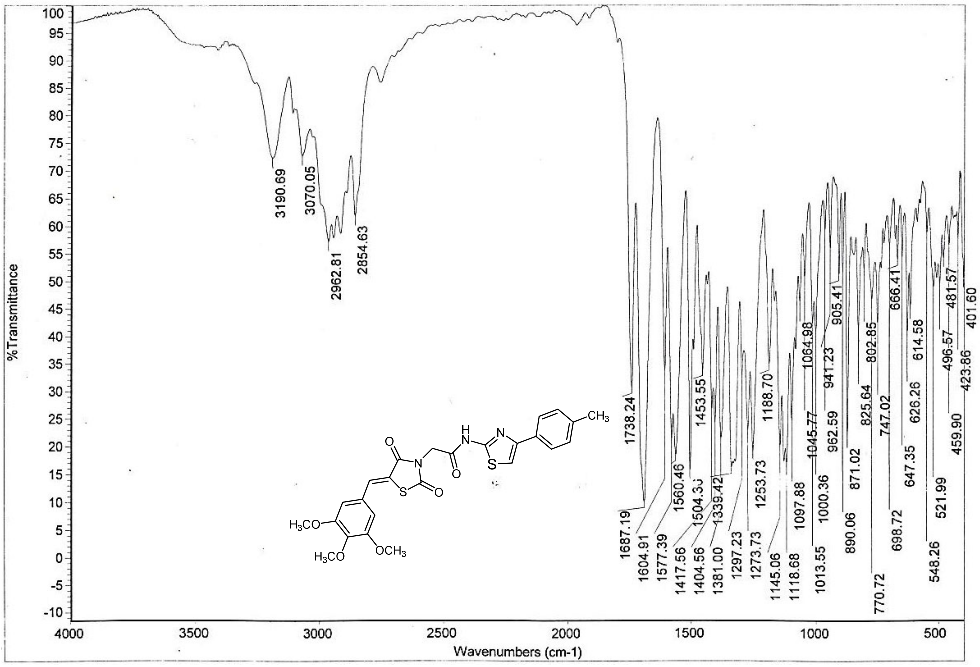

Fig. S22. IR spectrum of (Z)-2-(5-(3,4,5-trimethoxybenzylidene)thiazolidin-2,4-dion-3-yl)-N-(4-(4-methylphenyl)thiazol-2-yl)acetamide (**7c**).

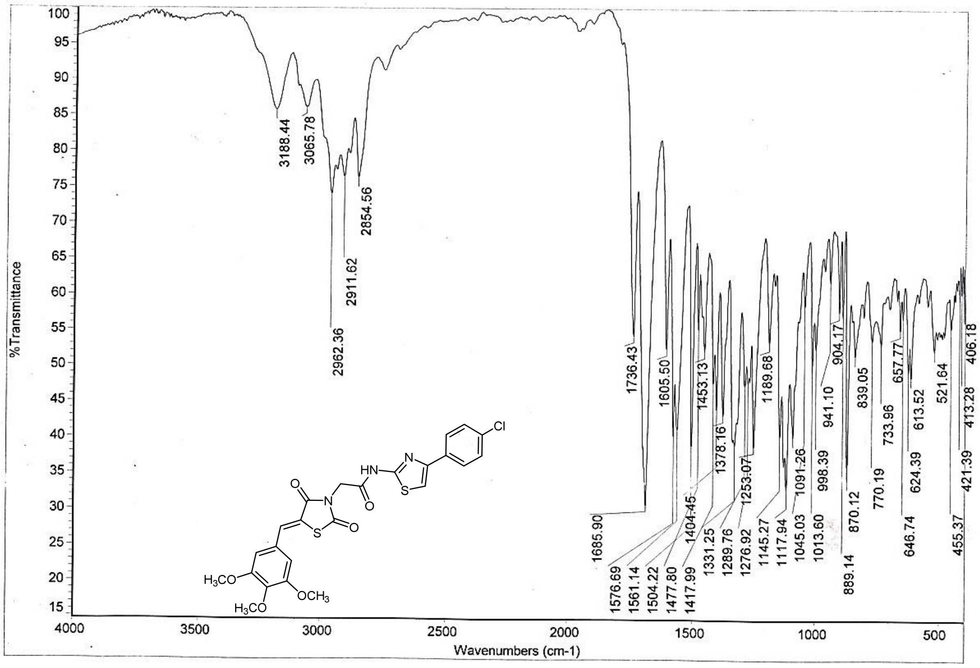

Fig. S23. IR spectrum of (*Z*)-2-(5-(3,4,5-trimethoxybenzylidene)thiazolidin-2,4-dion-3-yl)-N-(4-(4-chlorophenyl)thiazol-2-yl)acetamide (**7d**).

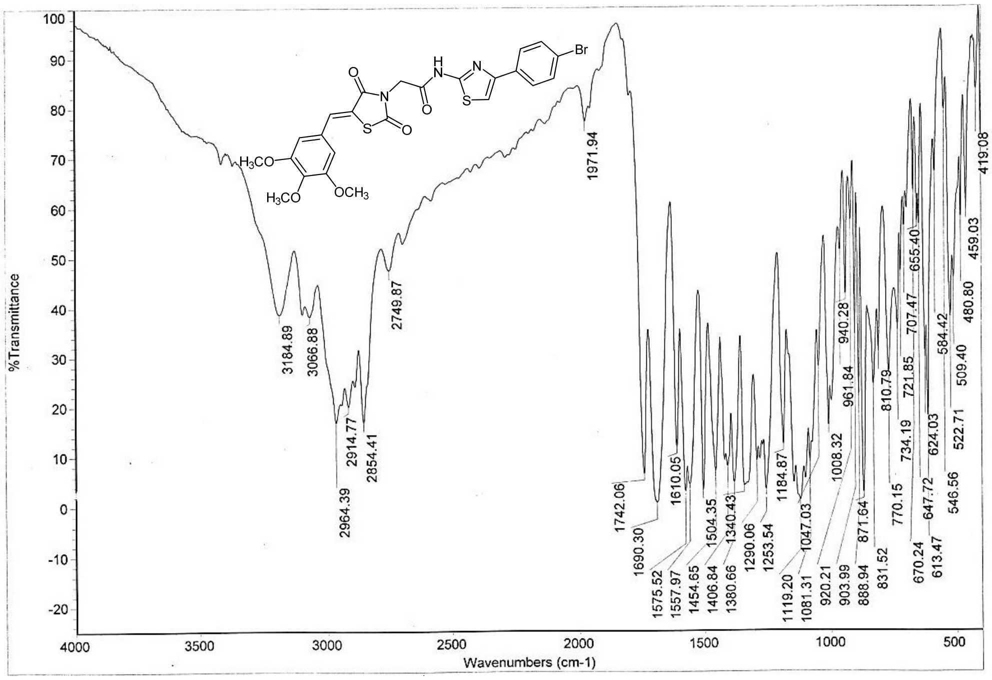

Fig. S24. IR spectrum of (*Z*)-2-(5-(3,4,5-trimethoxybenzylidene)thiazolidin-2,4-dion-3-yl)-N-(4-(4-bromophenyl)thiazol-2-yl)acetamide (**7e**).

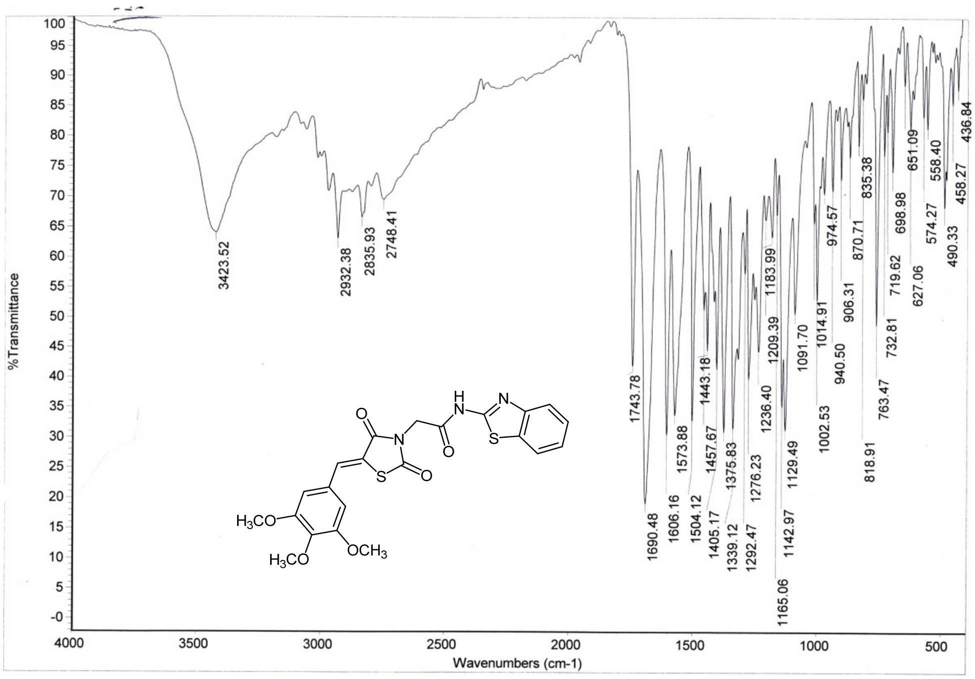

Fig. S25. IR spectrum of (*Z*)-2-(5-(3,4,5-trimethoxybenzylidene)thiazolidin-2,4-dion-3-yl)-N-(benzo[*d*]thiazol-2-yl)acetamide (**7e**).

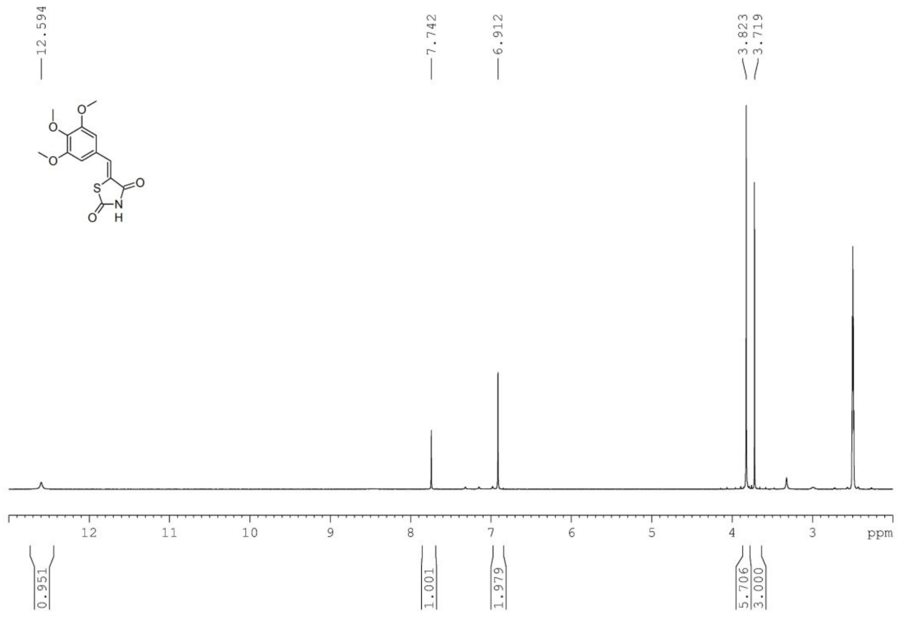

Fig. S26. ^{1}H-NMR spectrum of (*Z*)-5-(3,4,5-Trimethoxybenzylidene) thiazolidine-2,4-dione **(2).**

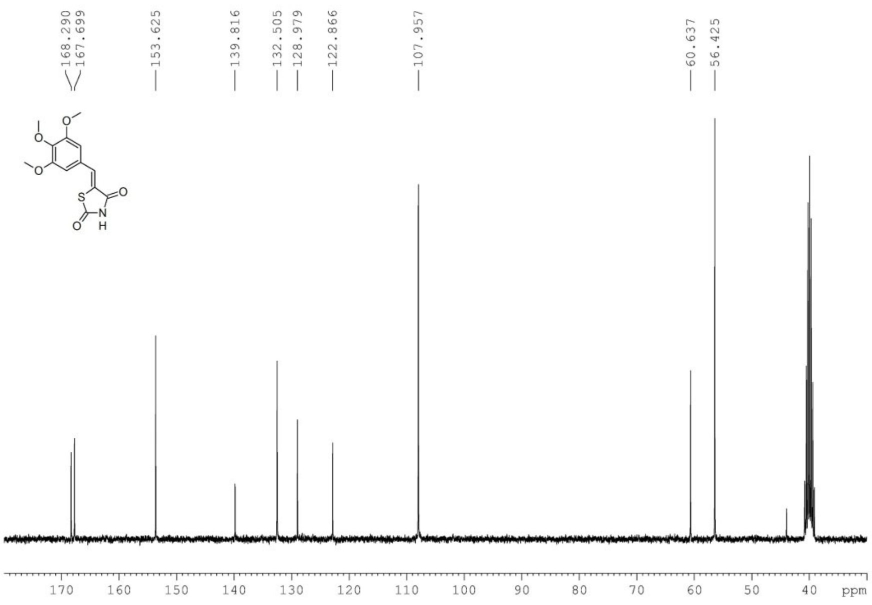

Fig. S27. ^{13}C-NMR spectrum of (*Z*)-5-(3,4,5-Trimethoxybenzylidene) thiazolidine-2,4-dione **(2).**

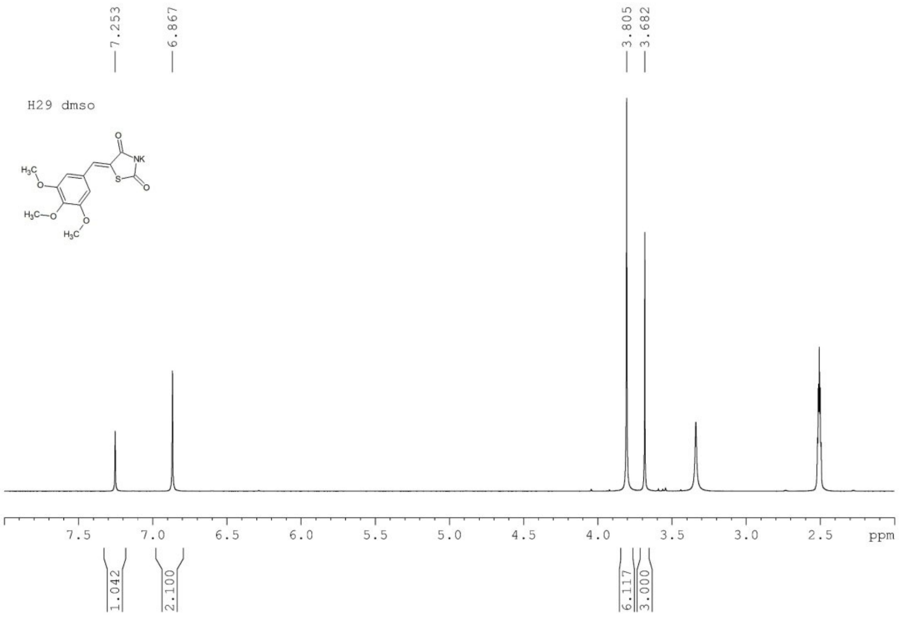

Fig. S28. ^{1}H-NMR spectrum of potassium (Z)-5-(3,4,5-Trimethoxybenzylidene)thiazolidine-2,4-dion-3-ide (**3**).

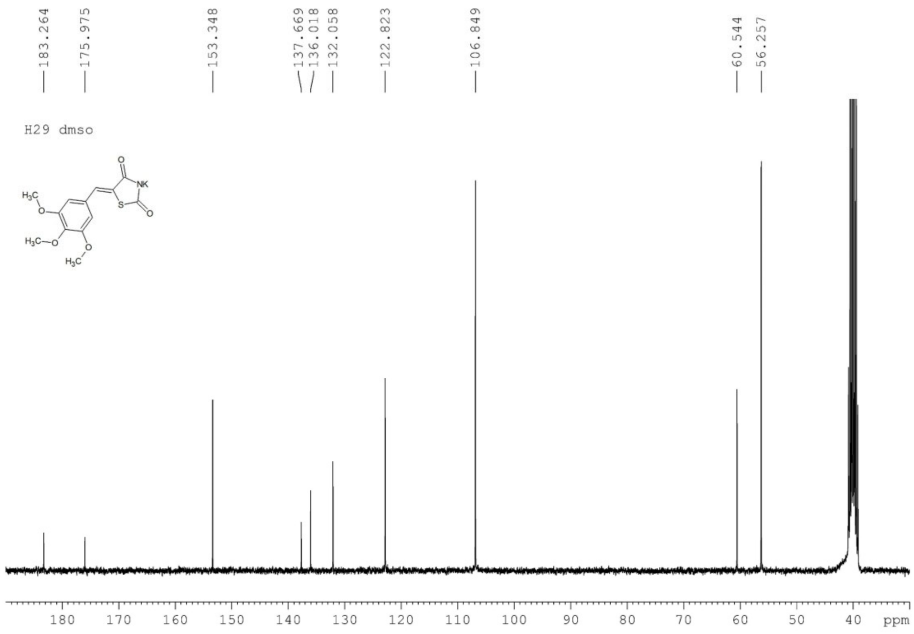

Fig. S29. ^{13}C-NMR spectrum of potassium (Z)-5-(3,4,5-Trimethoxybenzylidene)thiazolidine-2,4-dion-3-ide (**3**).

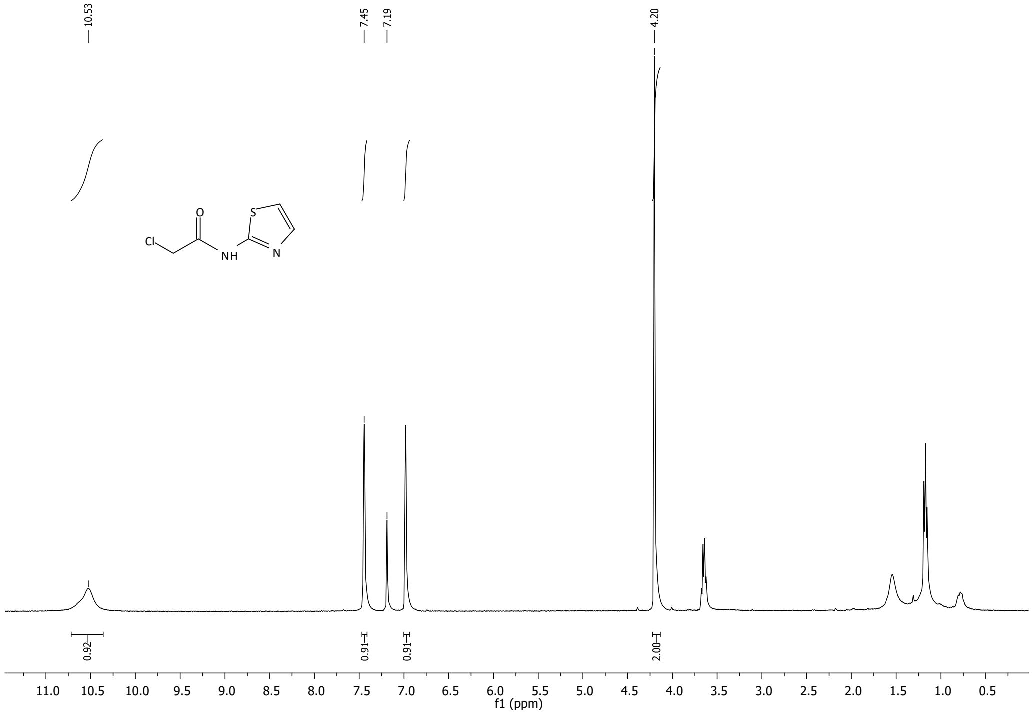

Fig. S30. ^{1}H-NMR spectrum of 2-chloro-N-(thiazol-2-yl)acetamide (**5a**). $CDCl_3$

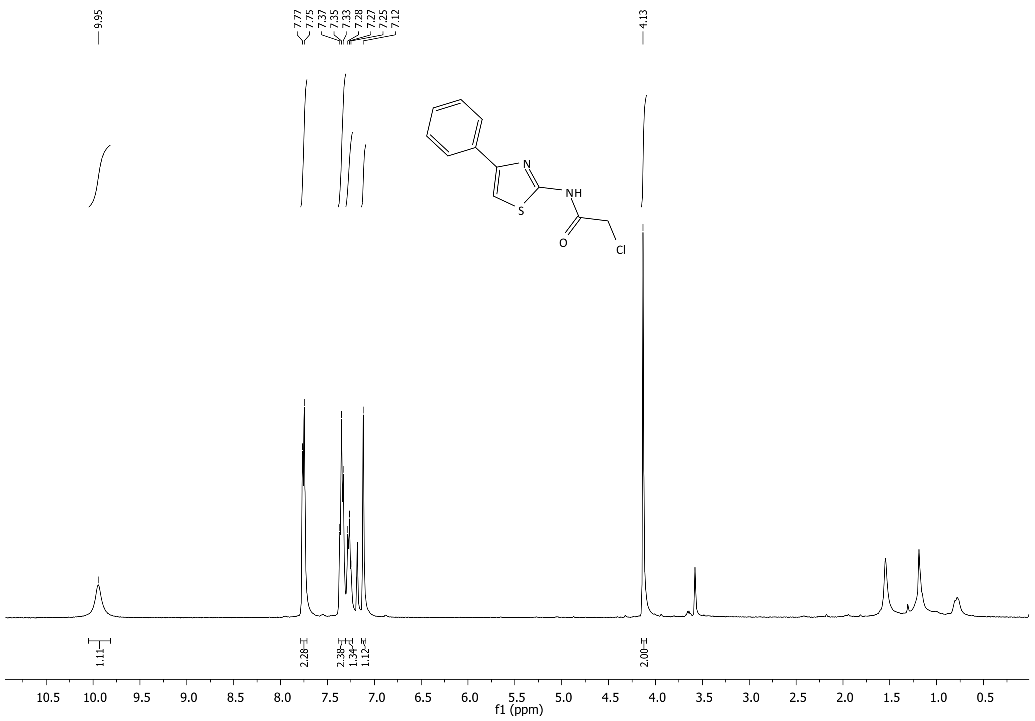

Fig. S31. ^{1}H-NMR spectrum of 2-chloro-N-(4-phenylthiazol-2-yl)acetamide (**5b**).

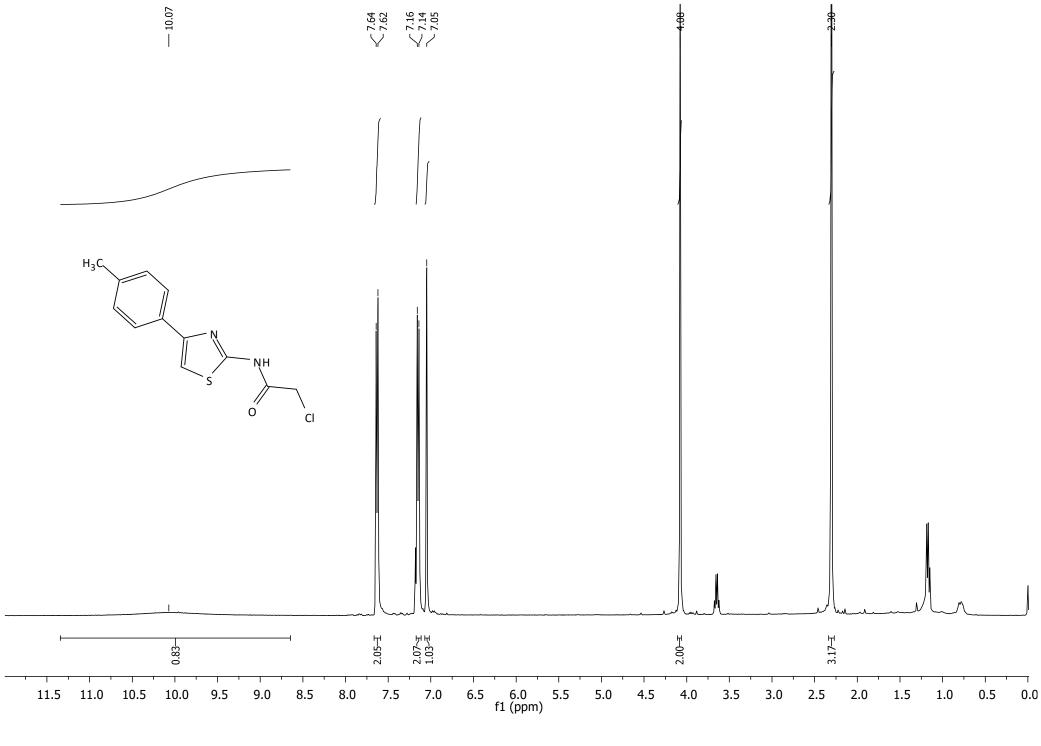

Fig. S32. ^{1}H-NMR spectrum ($CDCl_3$) of 2-chloro-N-(4-methylphenylthiazol-2-yl)acetamide (**5c**).

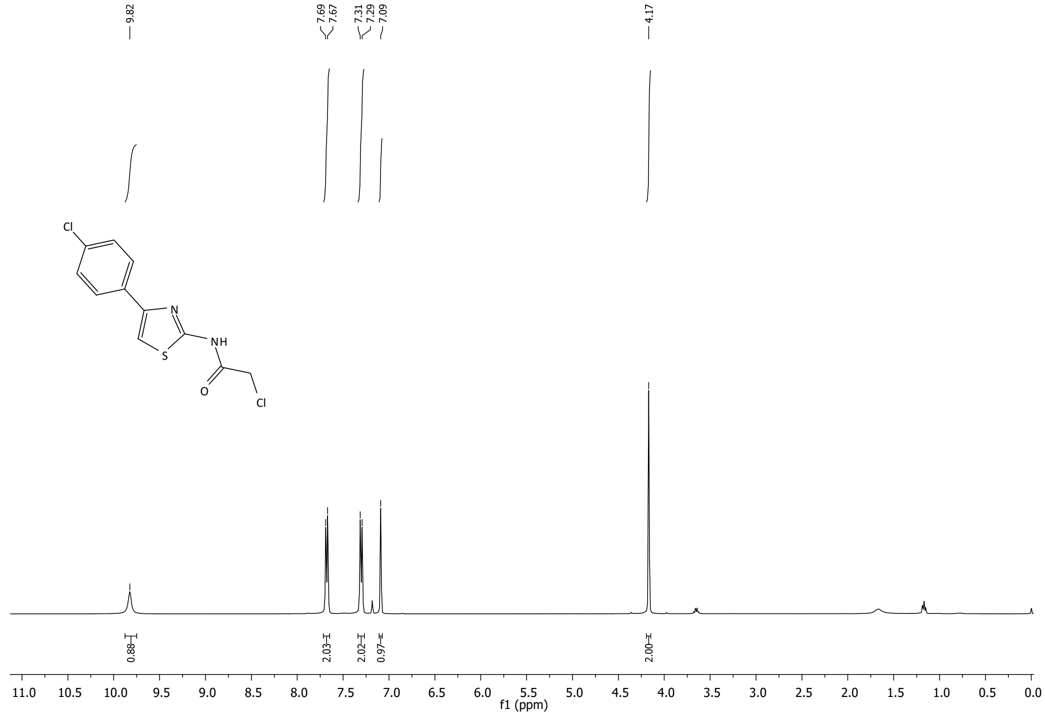

Fig.S33. ^{1}H-NMR spectrum ($CDCl_3$) of 2-chloro-N-(4-chlorophenylthiazol-2-yl)acetamide (**5d**).

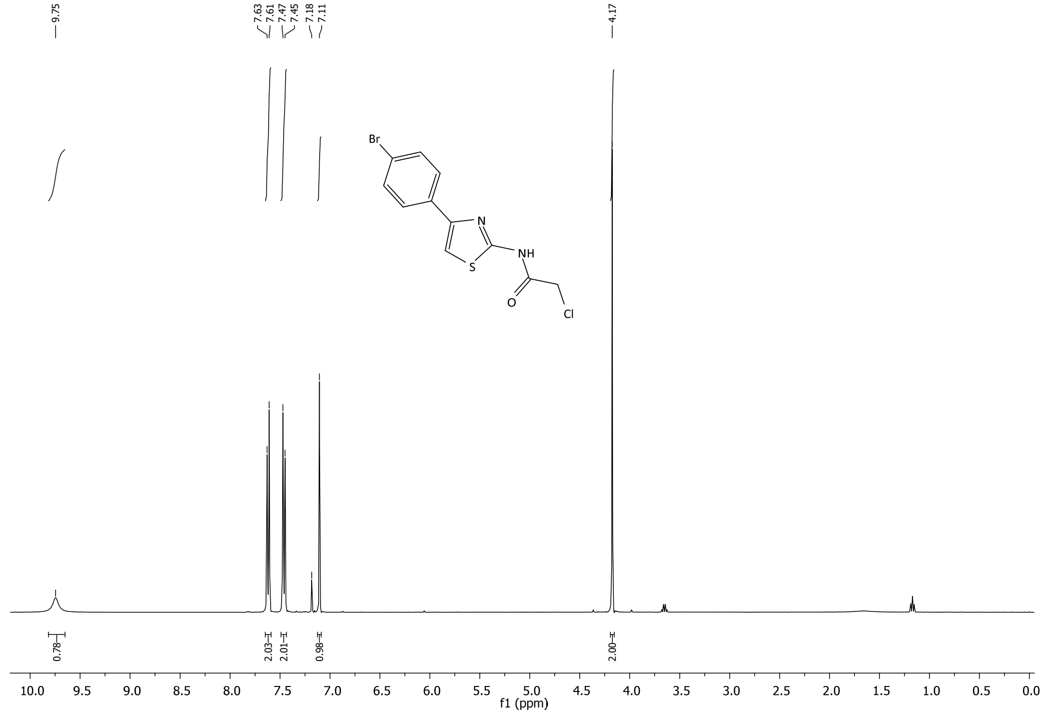

Fig. S34. ^{1}H-NMR spectrum ($CDCl_3$) of 2-chloro -N-(4-bromophenylthiazol-2-yl)acetamide (**5e**).

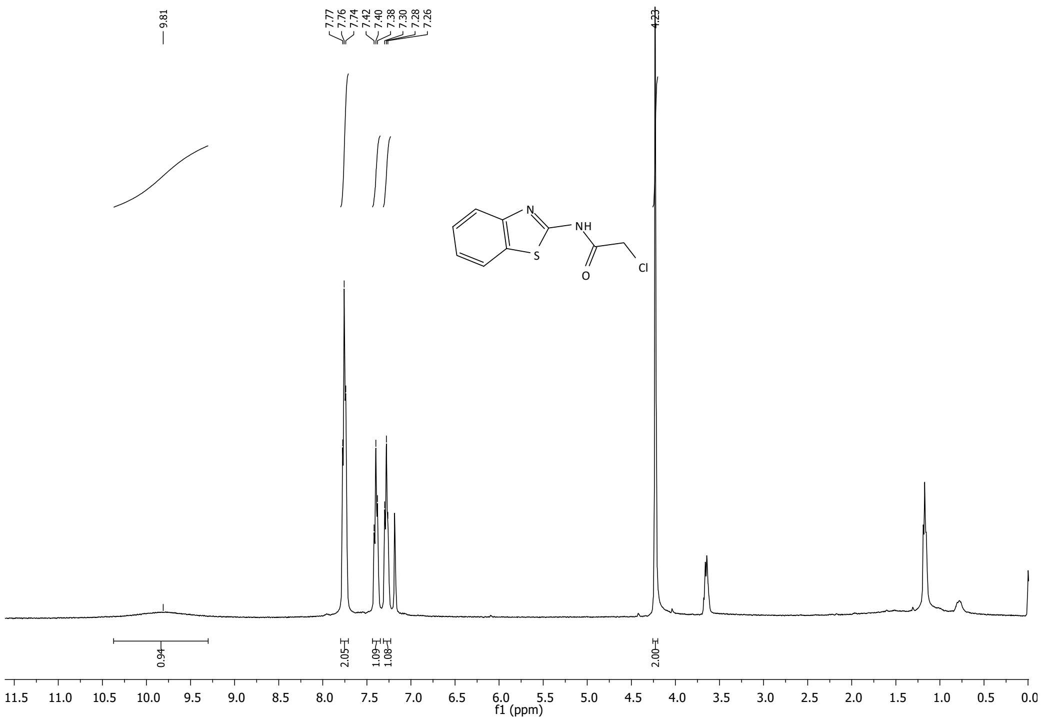

Fig. S35. ^{1}H-NMR spectrum ($CDCl_3$) of 2-chloro-N-(benzo[*d*]thiazol-2-yl)acetamide (**5f**).

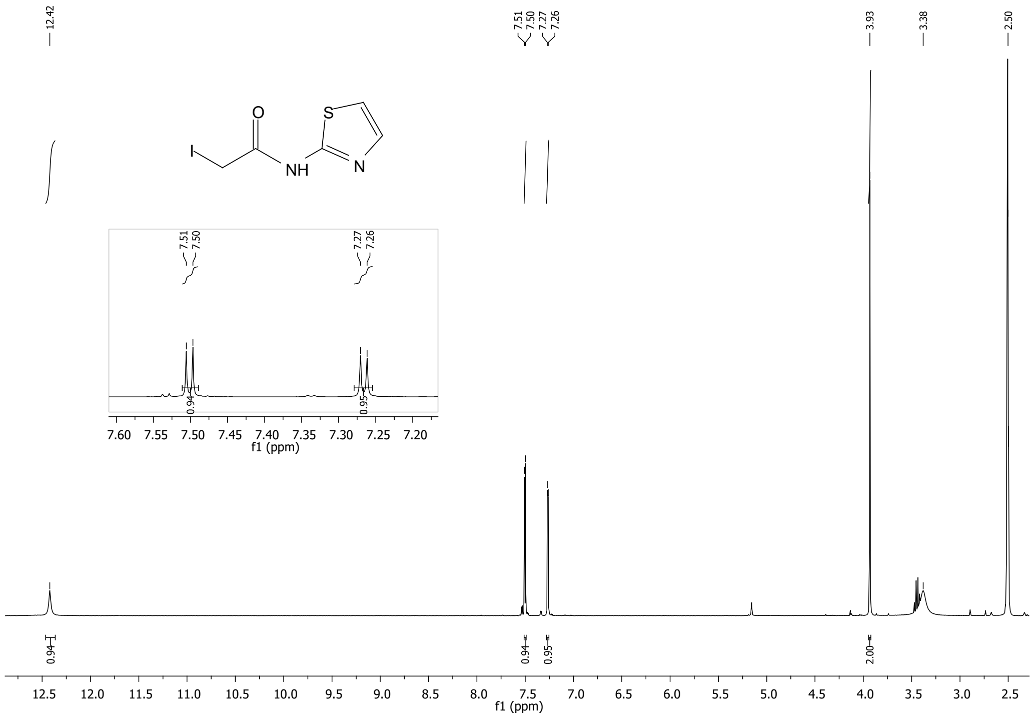

Fig. S36. ^{1}H-NMR spectrum of 2-iodo-N-(thiazol-2-yl)acetamide (**6a**).

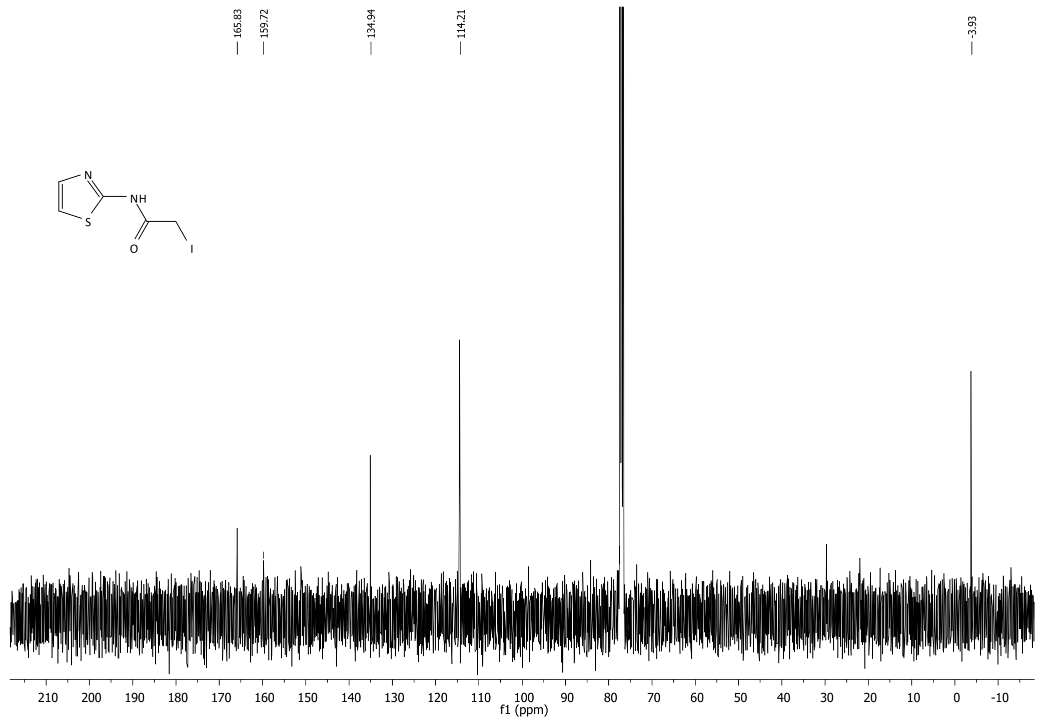

Fig. S37. ^{13}C-NMR spectrum ($CDCl_3$) of 2-iodo-N-(thiazol-2-yl)acetamide (**6a**).

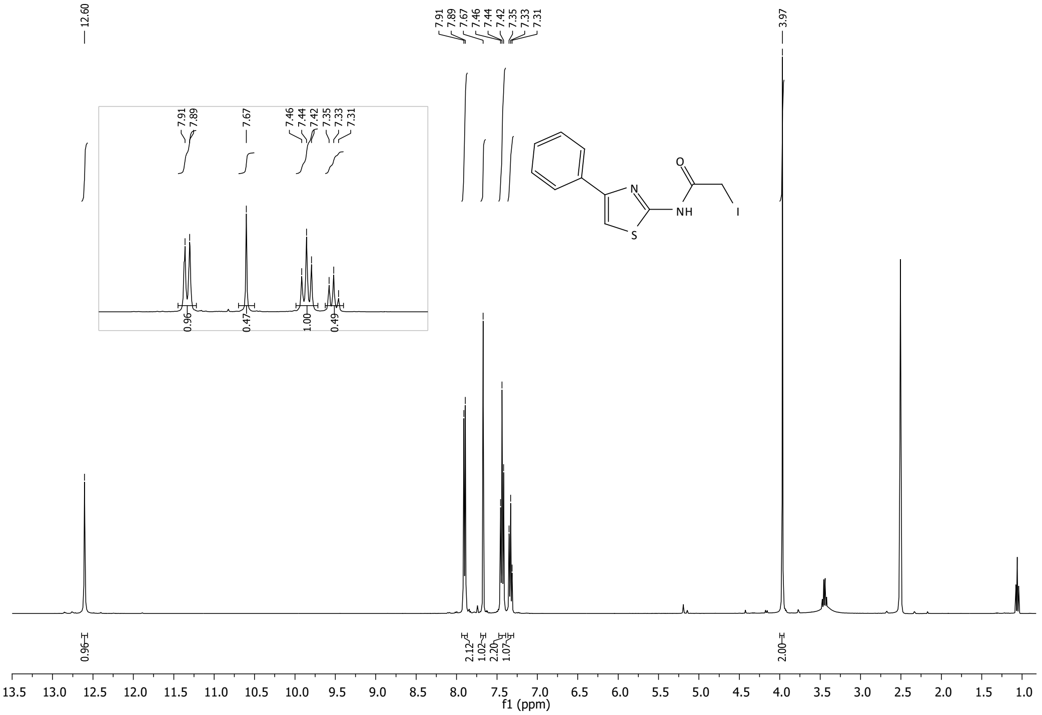

Fig. S38. ^{1}H-NMR spectrum of 2-iodo-N-(4-phenylthiazol-2-yl)acetamide (**6b**).

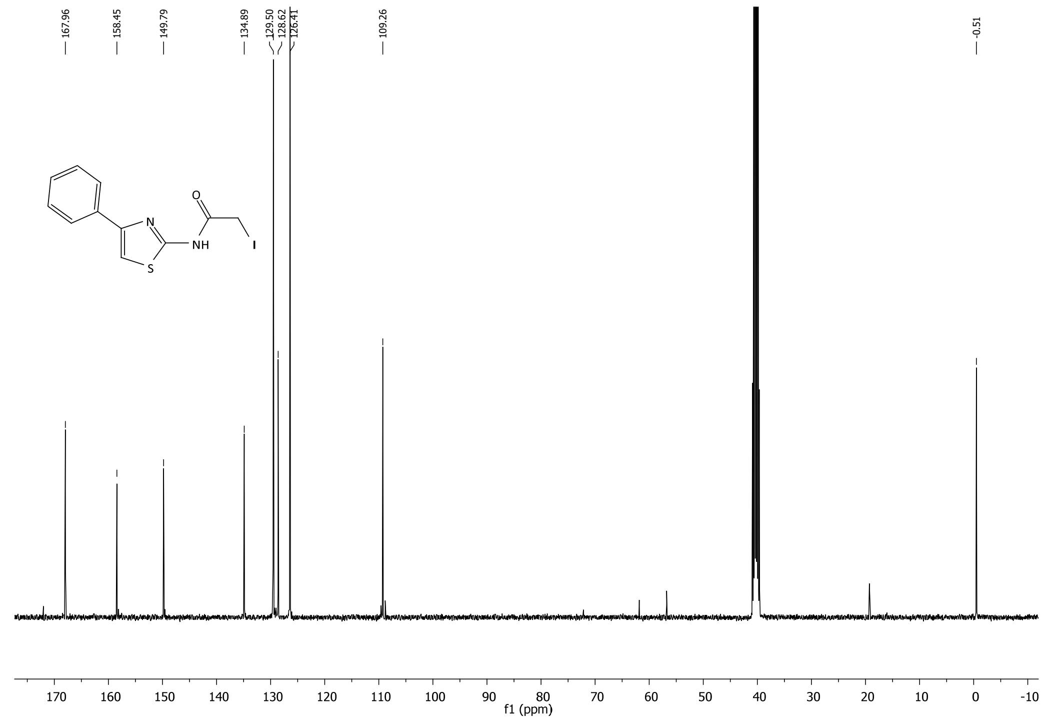

Fig. S39. ^{13}C-NMR spectrum of 2-iodo-N-(4-phenylthiazol-2-yl)acetamide (**6b**).

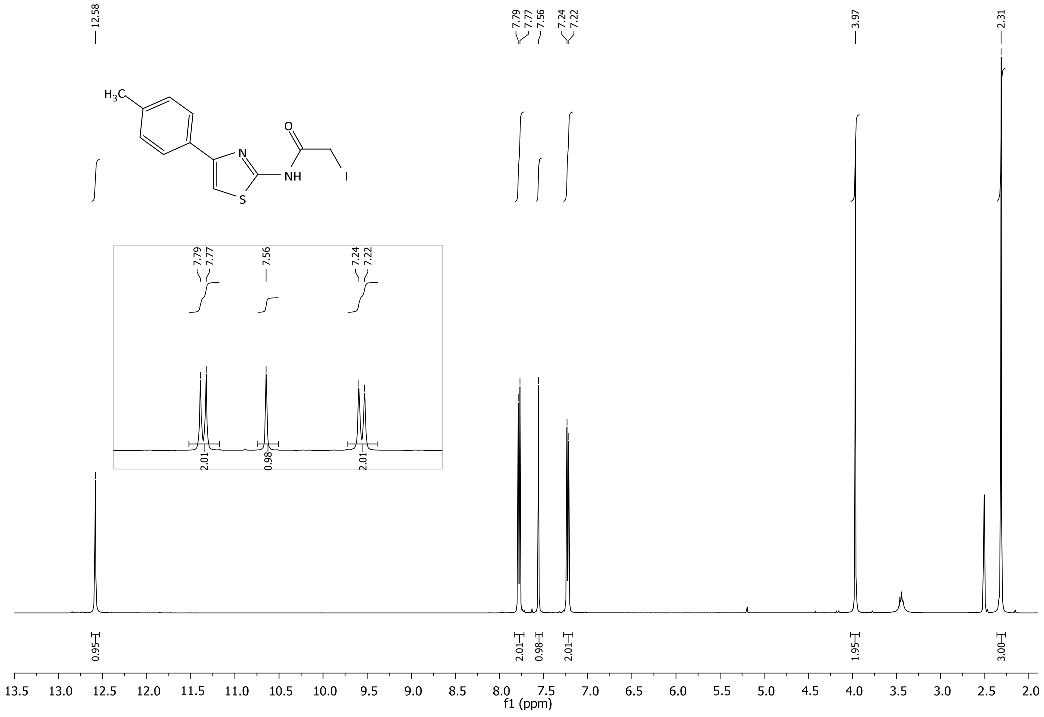

Fig. S40. ^{1}H-NMR spectrum of 2-iodo-N-(4-methylphenylthiazol-2-yl)acetamide (**6c**).

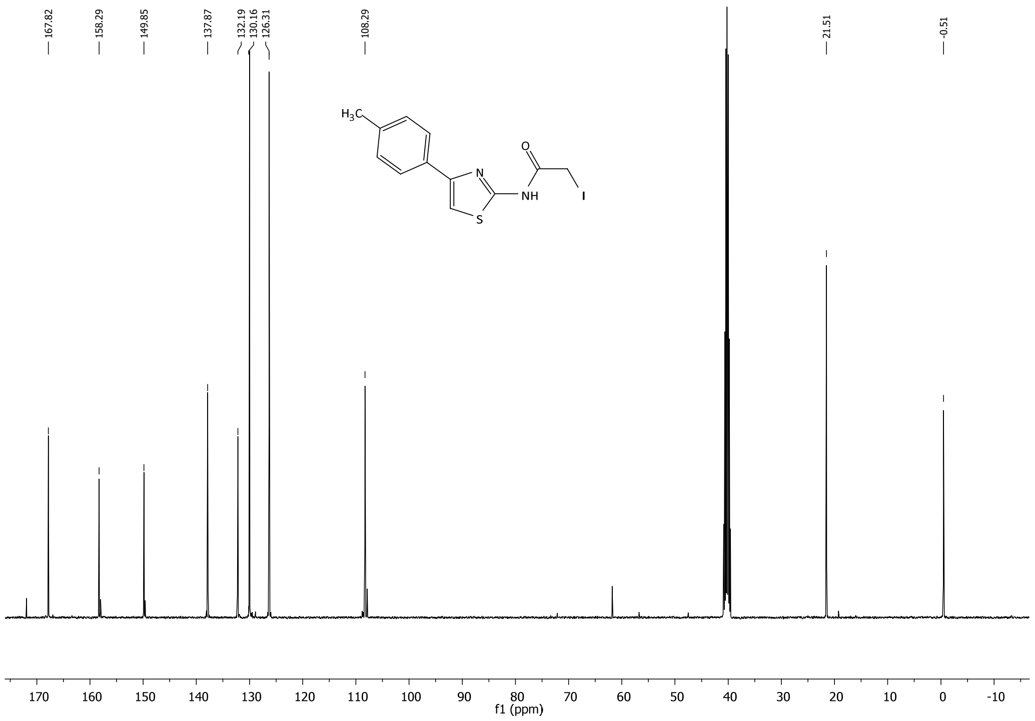

Fig. S41. ^{13}C-NMR spectrum of 2-iodo-N-(4-methyl phenylthiazol-2-yl)acetamide (**6c**).

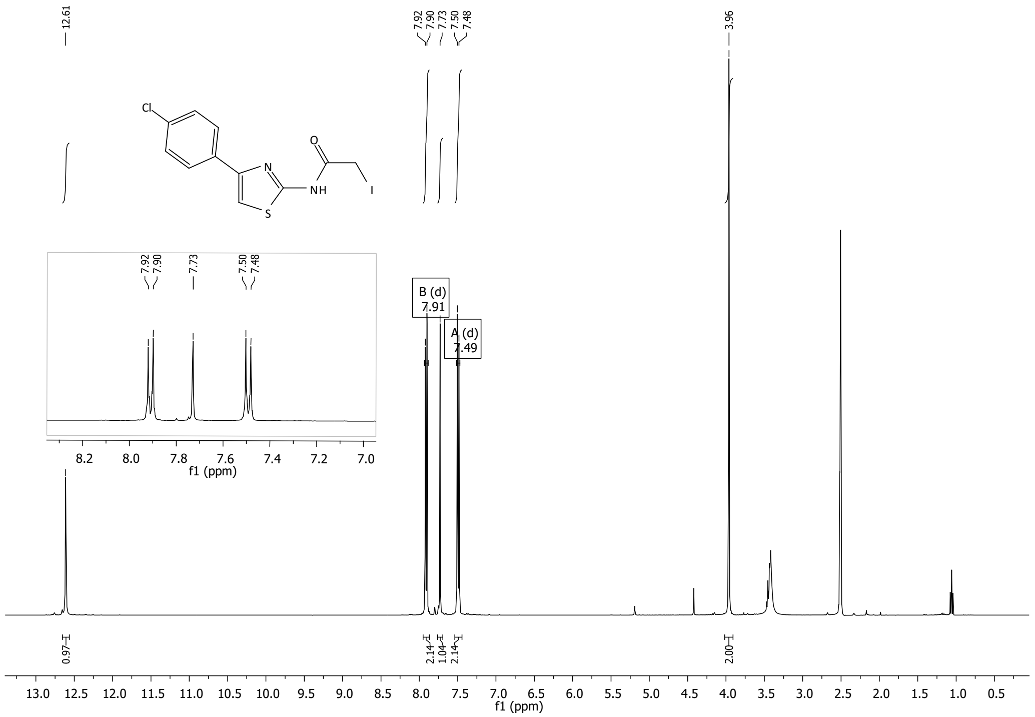

Fig. S42. ^{1}H-NMR spectrum of 2-iodo-N-(4-chlorophenylthiazol-2-yl)acetamide (**6d**).

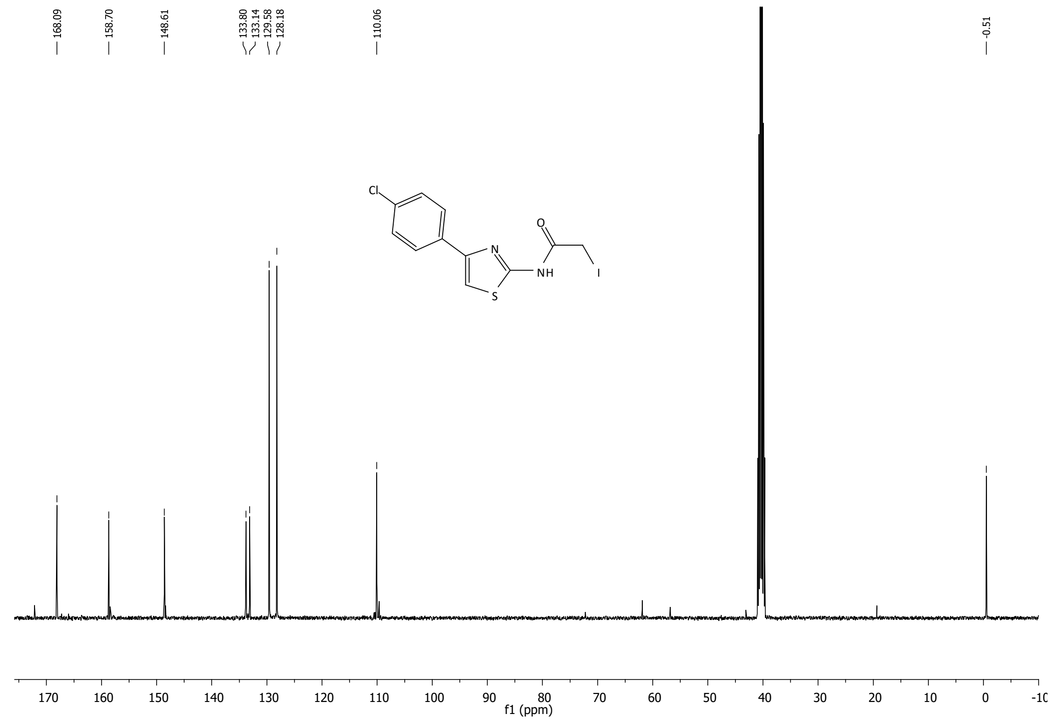

Fig. S43. ^{13}C-NMR spectrum of 2-iodo-N-(4-chlorophenylthiazol-2-yl)acetamide (**6d**).

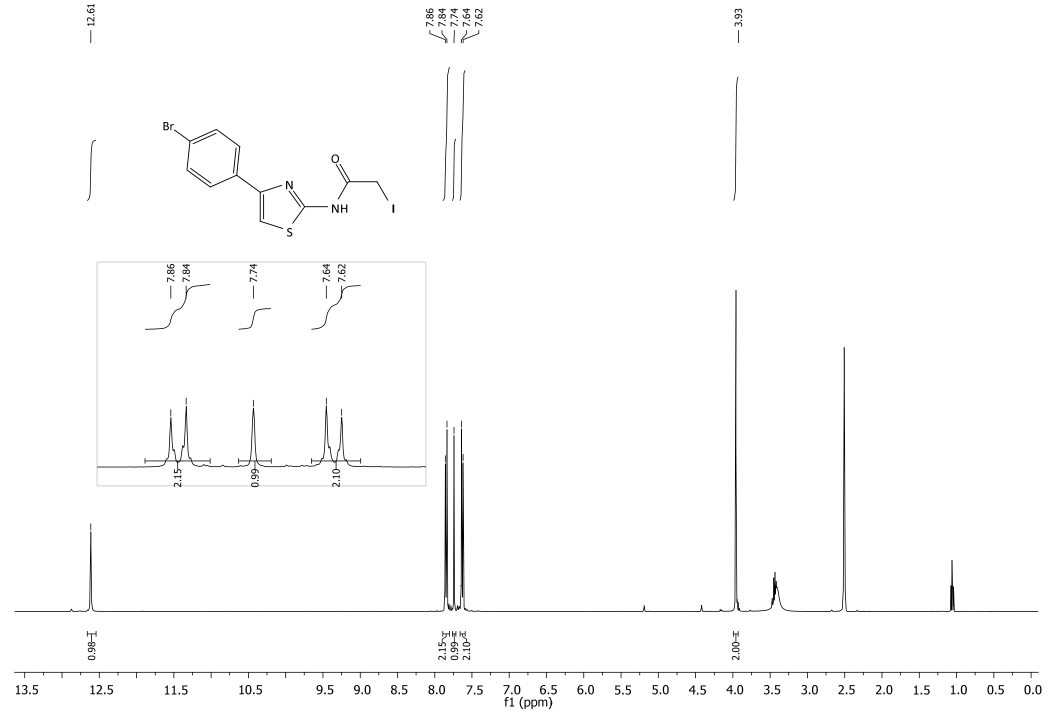

Fig. S44. ^{1}H-NMR spectrum of 2-iodo -N-(4-bromophenylthiazol-2-yl)acetamide (**6e**).

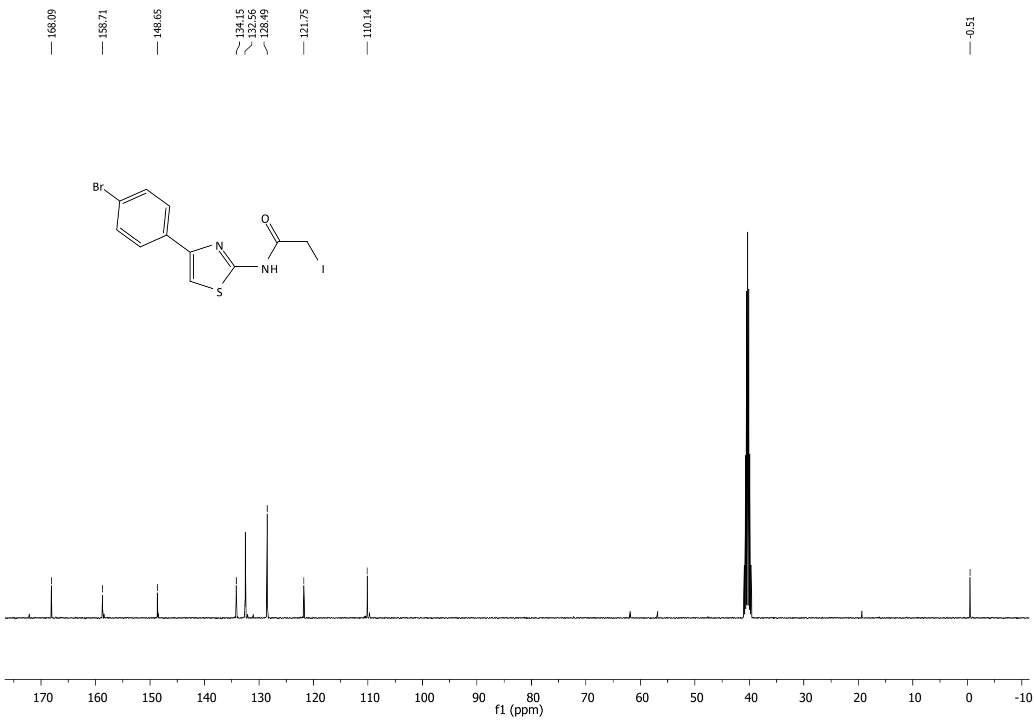

Fig. S45. ^{13}C-NMR spectrum of 2-iodo -N-(4-bromophenylthiazol-2-yl)acetamide (**6e**).

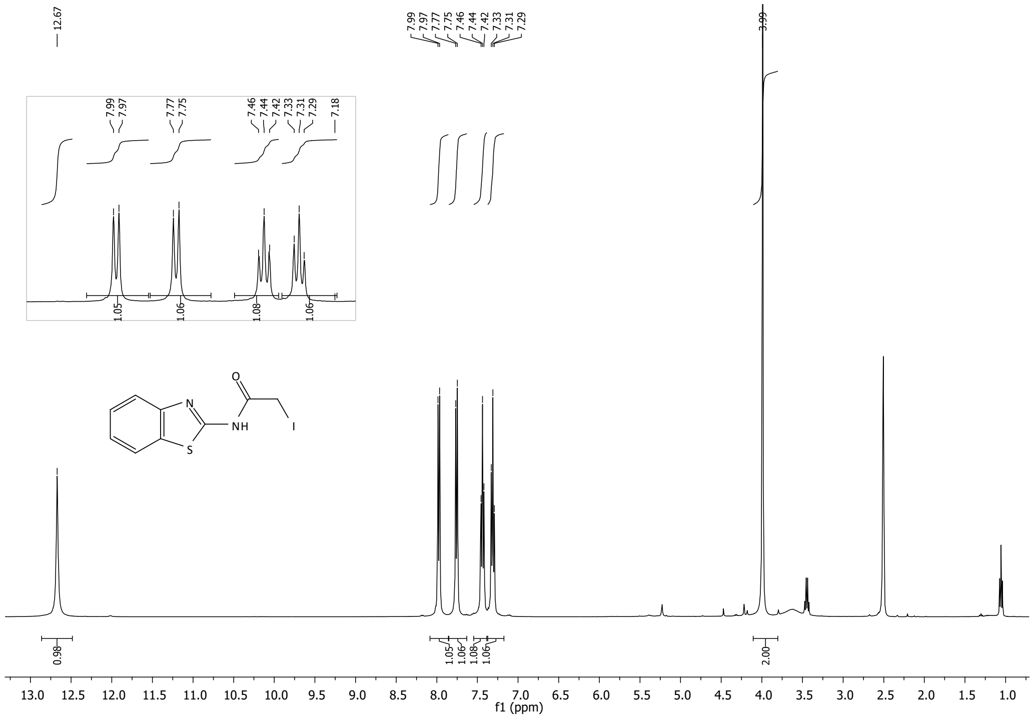

Fig. S46. ^{1}H-NMR spectrum of 2-iodo-N-(benzo[*d*]thiazol-2-yl)acetamide (**6f**).

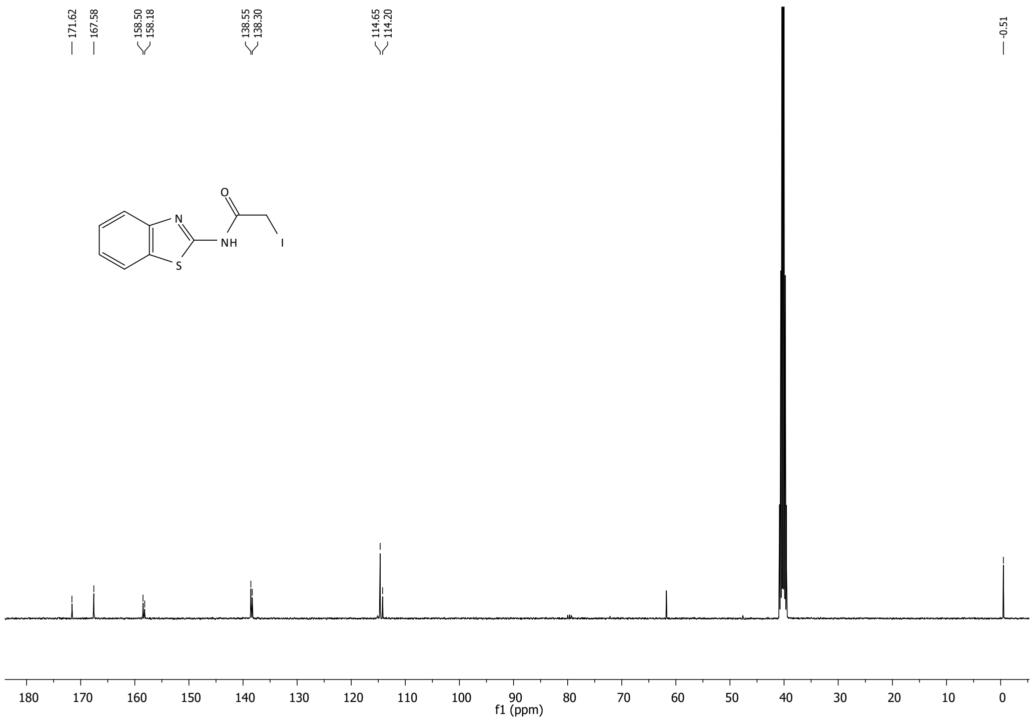

Fig. S47. ^{13}C-NMR spectrum of 2-iodo-N-(benzo[*d*]thiazol-2-yl)acetamide (**6f**).

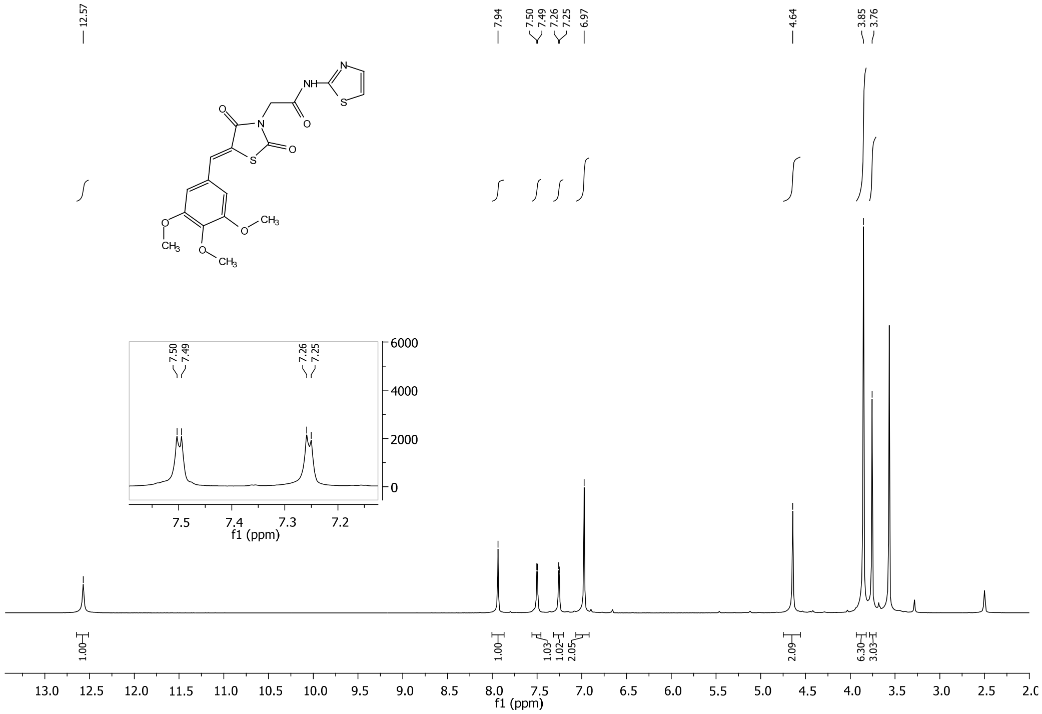

Fig. S48. ^{1}H-NMR spectrum of (*Z*)-2-(5-(3,4,5-trimethoxybenzlidene)thiazolidine-2,4-dion-3-yl)-N-(thiazole-2-yl)acetamide (**7a**).

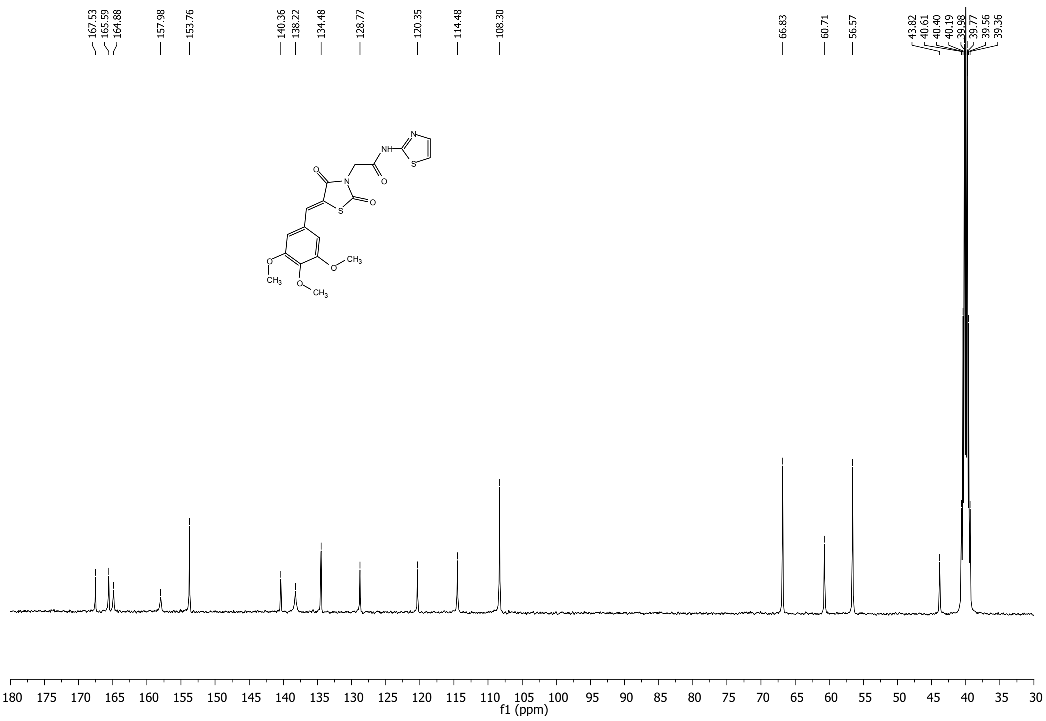

Fig. S49. ^{13}C-NMR spectrum of (*Z*)-2-(5-(3,4,5-trimethoxybenzlidene)thiazolidine-2,4-dion-3-yl)-N-(thiazole-2-yl)acetamide (**7a**).

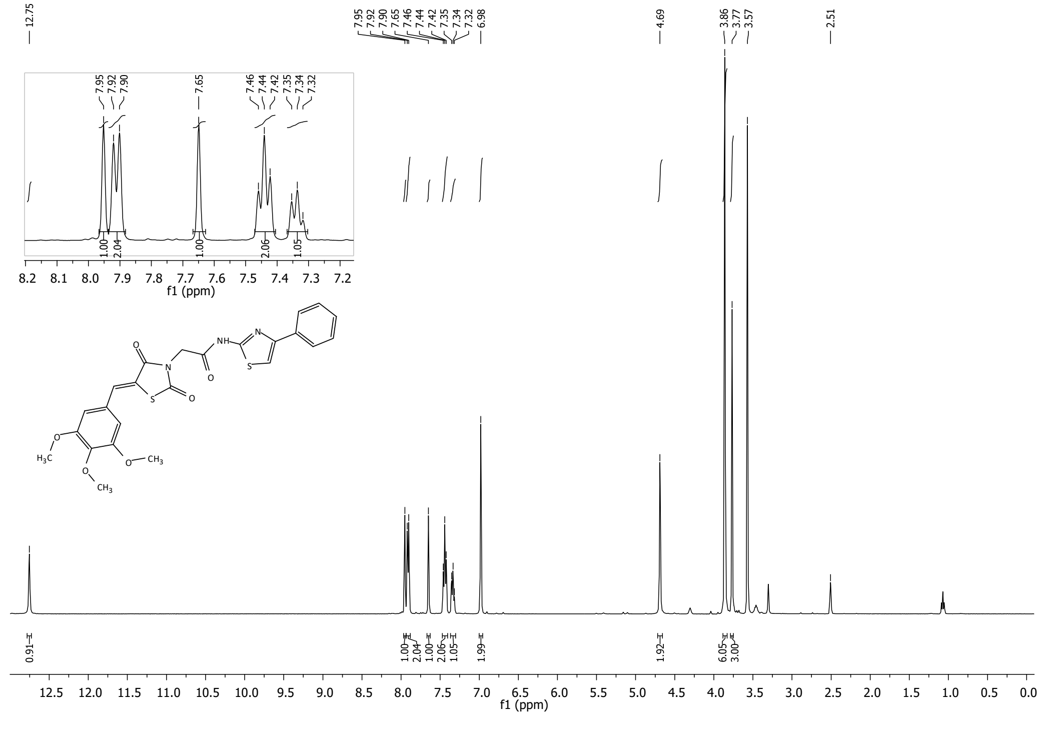

Fig. S50. ^{1}H-NMR spectrum of (*Z*)-2-(5-(3,4,5-trimethoxybenzlidene)thiazolidine-2,4-dion-3-yl)-N-(4-phenylthiazole-2-yl)acetamide (**7b**).

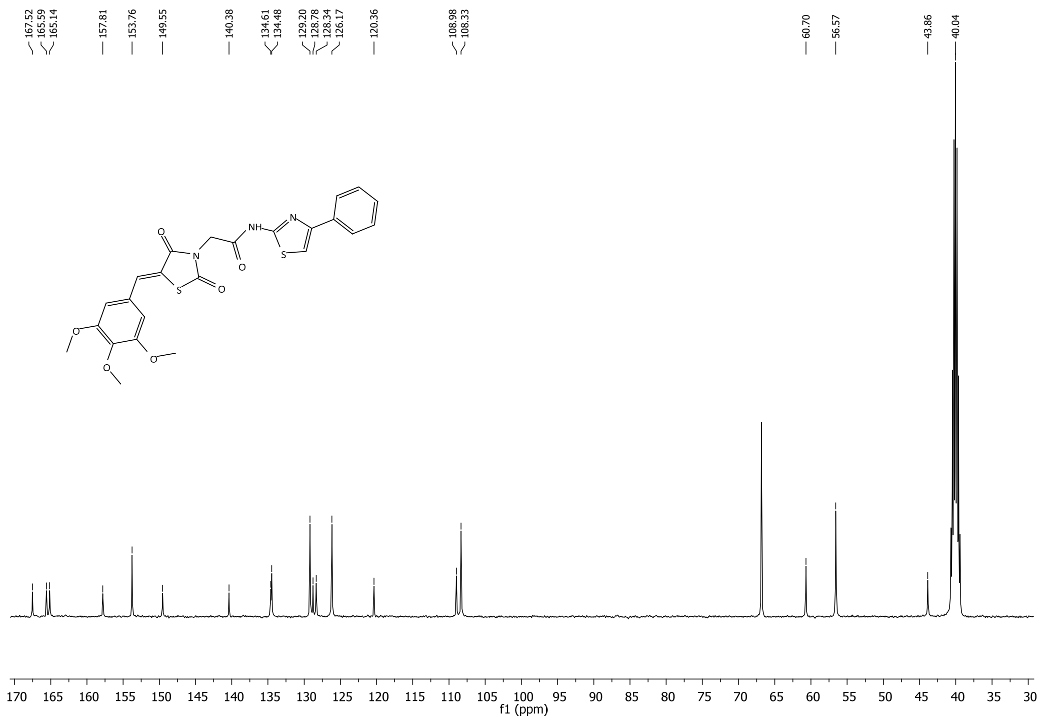

Fig. S51. ^{13}C-NMR spectrum of (*Z*)-2-(5-(3,4,5-trimethoxybenzlidene)thiazolidine-2,4-dion-3-yl)-N-(4-phenylthiazole-2-yl)acetamide (**7b**).

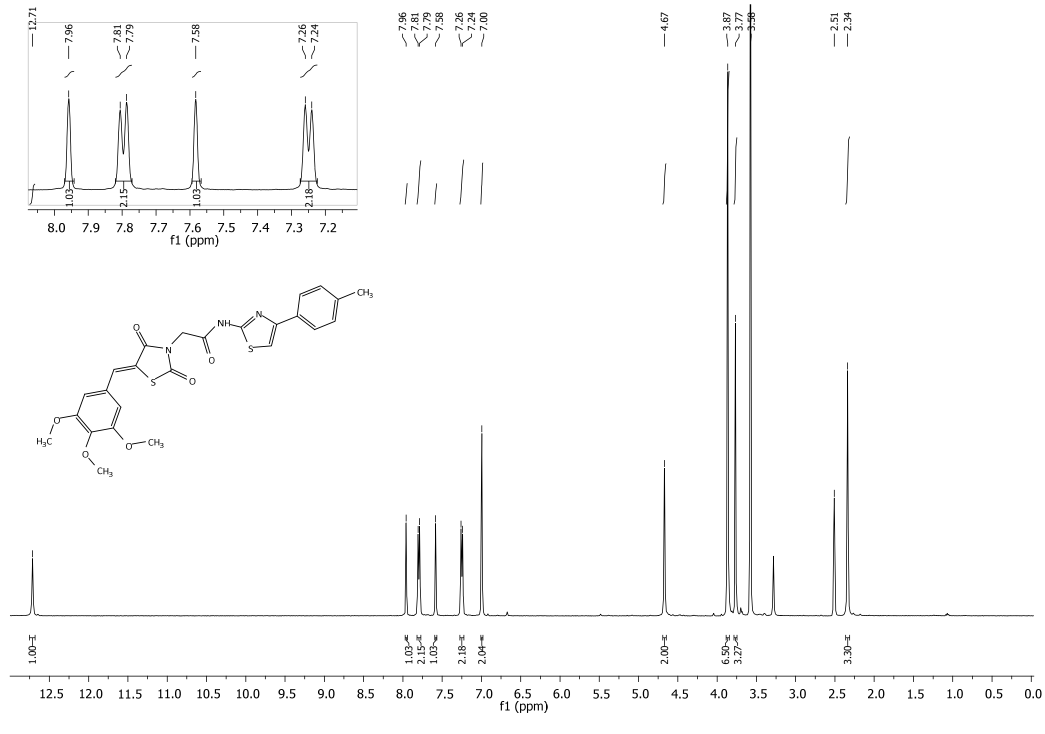

Fig. S52. ^{1}H-NMR spectrum of (Z)-2-(5-(3,4,5-trimethoxybenzylidene)thiazolidin-2,4-dion-3-yl)-N-(4-(4-methylphenyl)thiazol-2-yl)acetamide (**7c**).

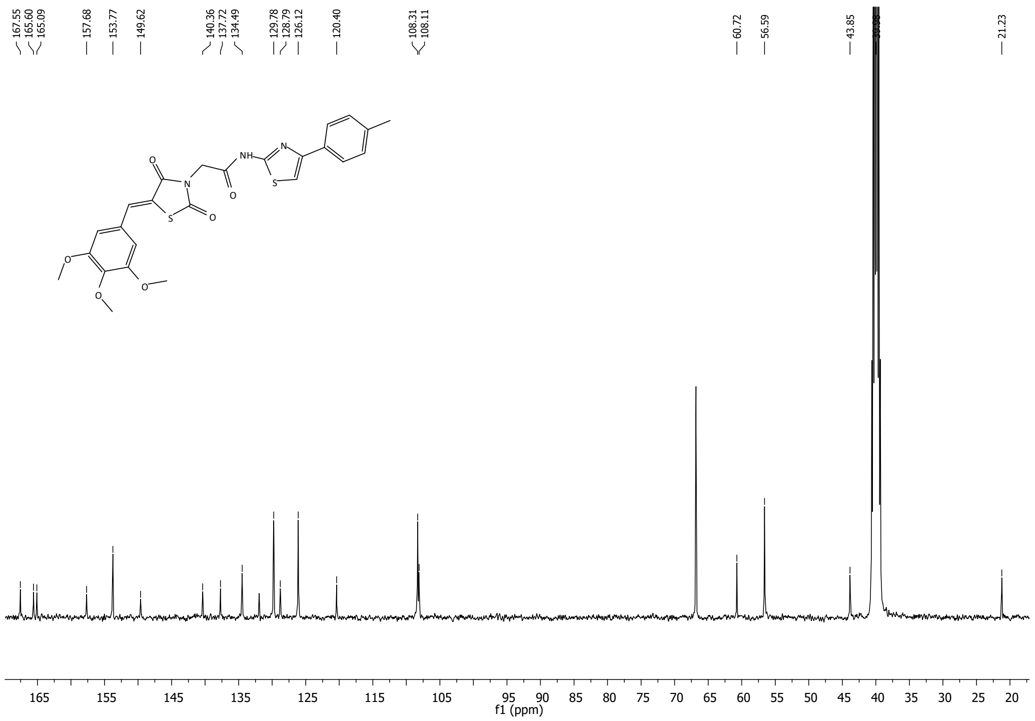

Fig. S53. ^{13}C-NMR spectrum of (Z)-2-(5-(3,4,5-trimethoxybenzylidene)thiazolidin-2,4-dion-3-yl)-N-(4-(4-methylphenyl)thiazol-2-yl)acetamide (**7c**).

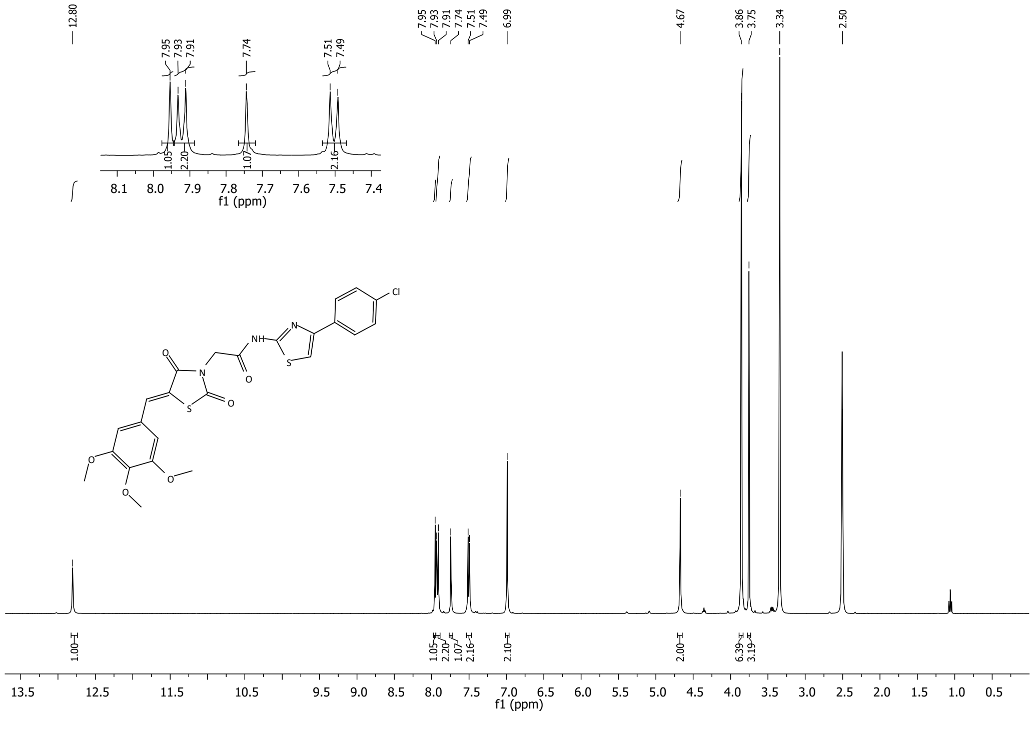

Fig. S54. ^{1}H-NMR spectrum of (*Z*)-2-(5-(3,4,5-trimethoxybenzylidene)thiazolidin-2,4-dion-3-yl)-N-(4-(4-chlorophenyl)thiazol-2-yl)acetamide (**7d**).

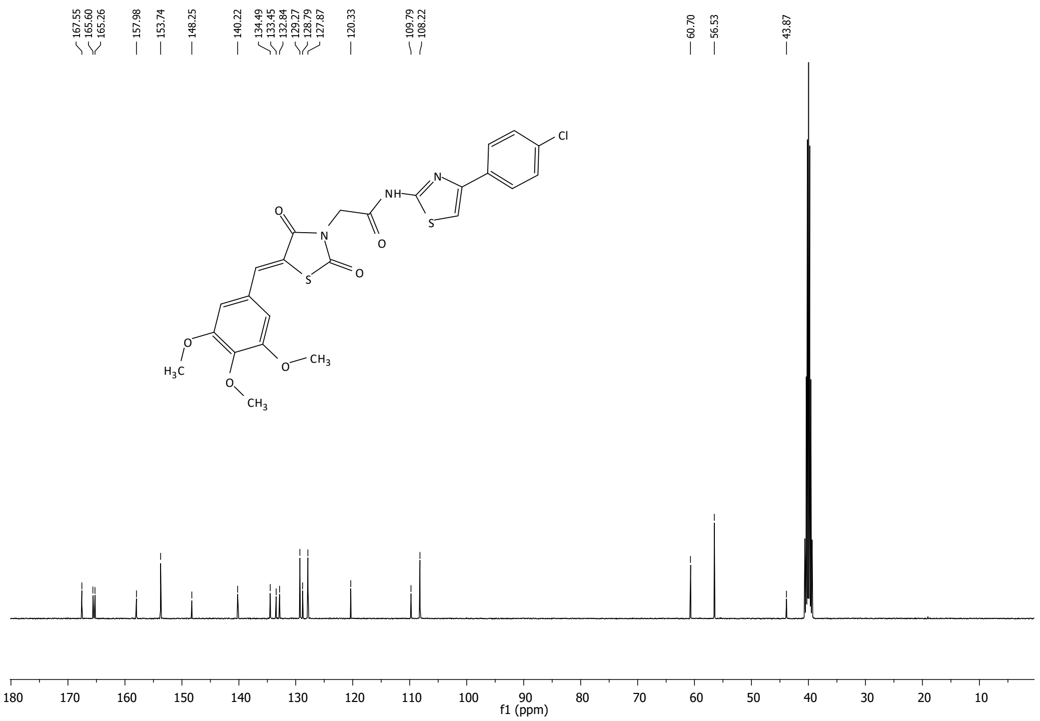

Fig. S55. ^{13}C-NMR spectrum of (*Z*)-2-(5-(3,4,5-trimethoxybenzylidene)thiazolidin-2,4-dion-3-yl)-N-(4-(4-chlorophenyl)thiazol-2-yl)acetamide (**7d**).

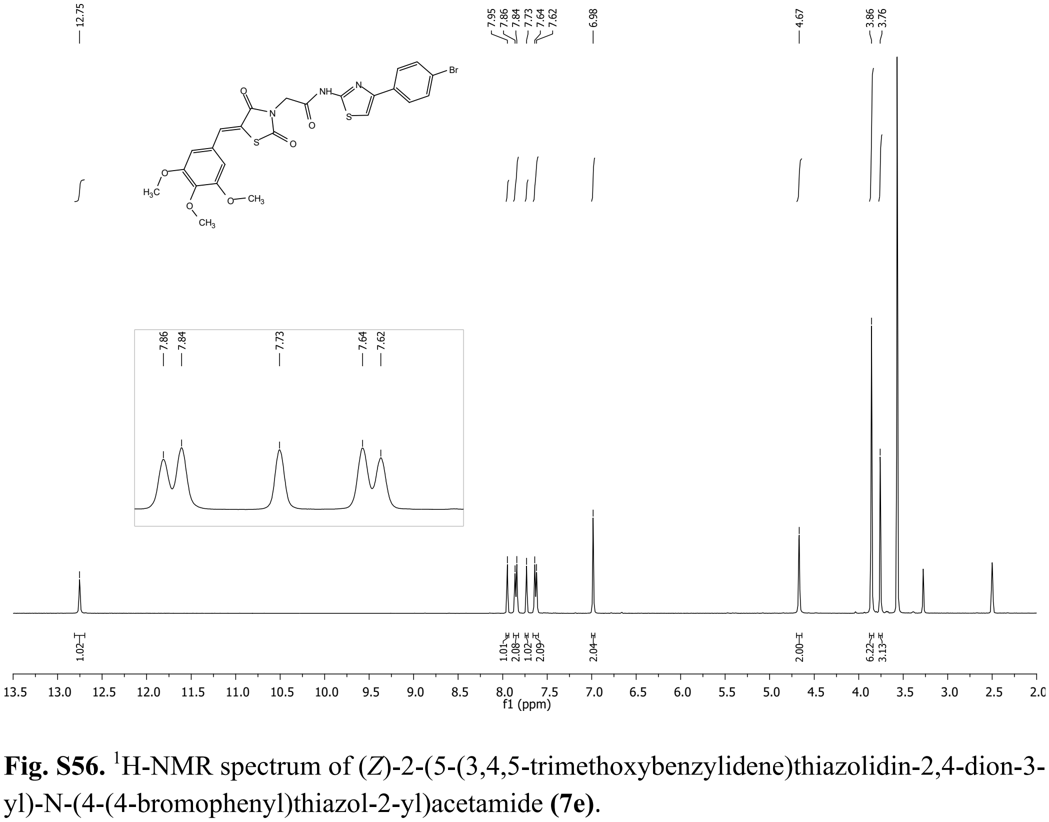

Fig. S56. ^{1}H-NMR spectrum of (*Z*)-2-(5-(3,4,5-trimethoxybenzylidene)thiazolidin-2,4-dion-3-yl)-N-(4-(4-bromophenyl)thiazol-2-yl)acetamide **(7e)**.

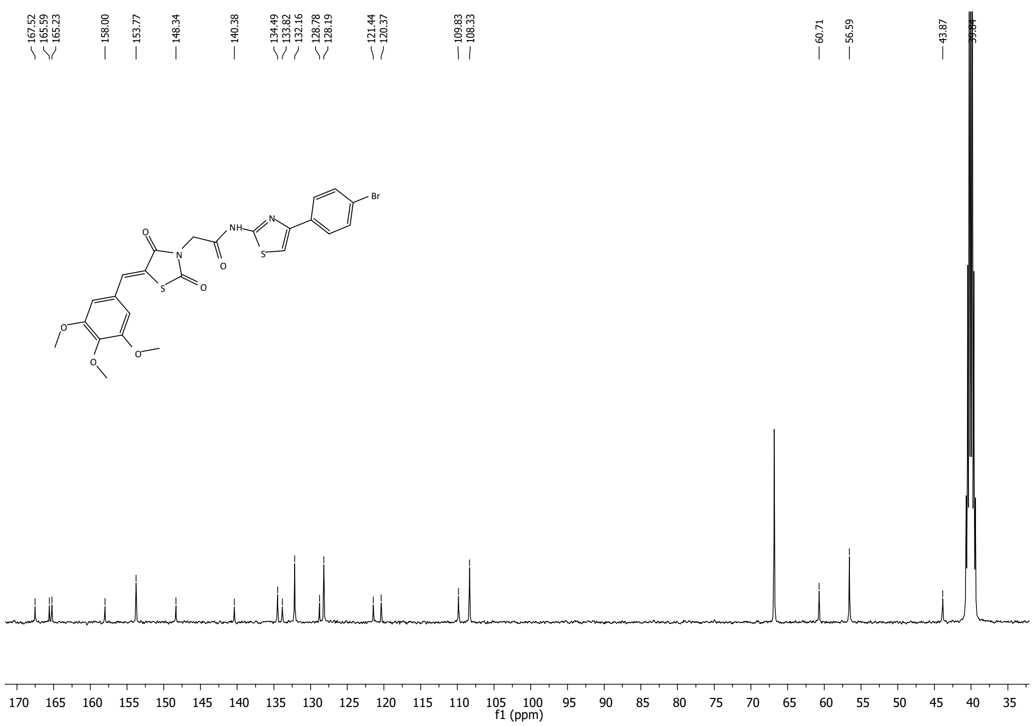

Fig. S57. ^{13}C-NMR spectrum of (*Z*)-2-(5-(3,4,5-trimethoxybenzylidene)thiazolidin-2,4-dion-3-yl)-N-(4-(4-bromophenyl)thiazol-2-yl)acetamide (**7e**).

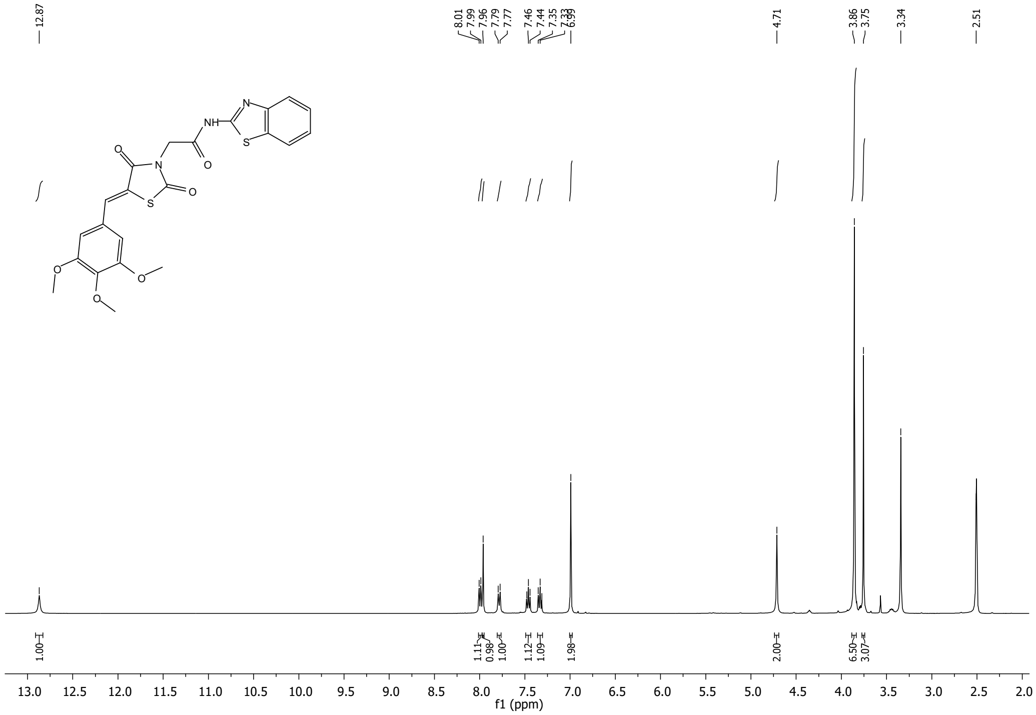

Fig. S58. ^{1}H-NMR spectrum of (*Z*)-2-(5-(3,4,5-trimethoxybenzylidene)thiazolidin-2,4-dion-3-yl)-N-(benzo[*d*]thiazol-2-yl)acetamide (**7f**).

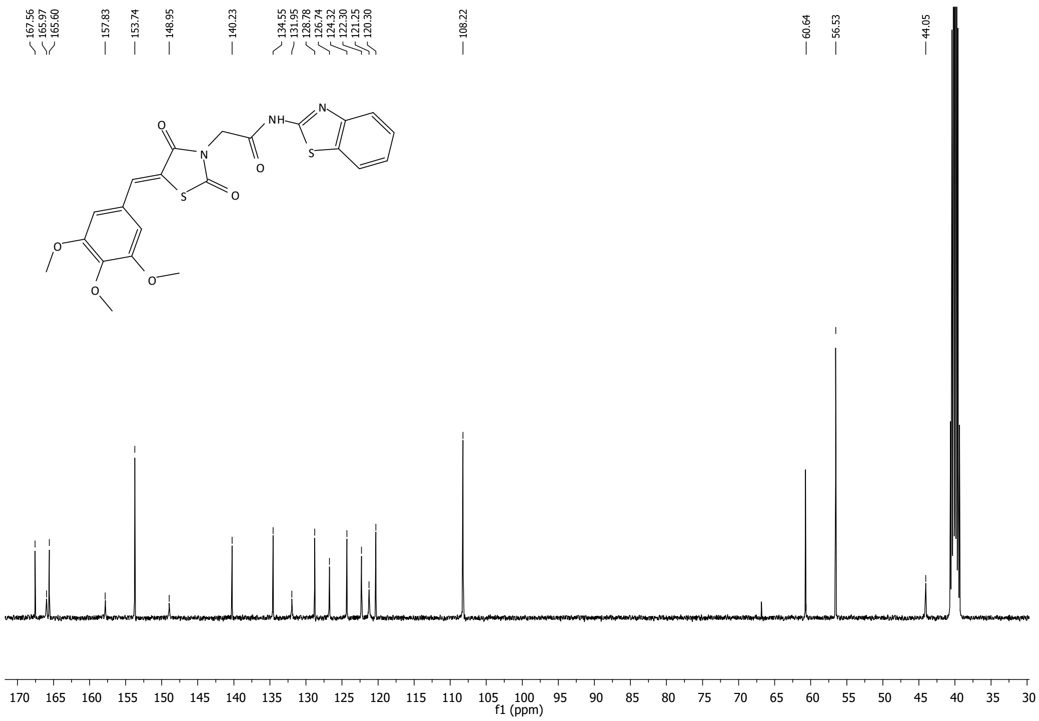
167.56
165.97
165.60
157.83
153.74
148.95
140.23
134.55
131.95
128.78
126.74
124.32
122.30
121.25
120.30
108.22
60.64
56.53
44.05
170 165 160 155 150 145 140 135 130 125 120 115 110 105 100 95 90 85 80 75 70 65 60 55 50 45 40 35 30
f1 (ppm)

Fig. S59.

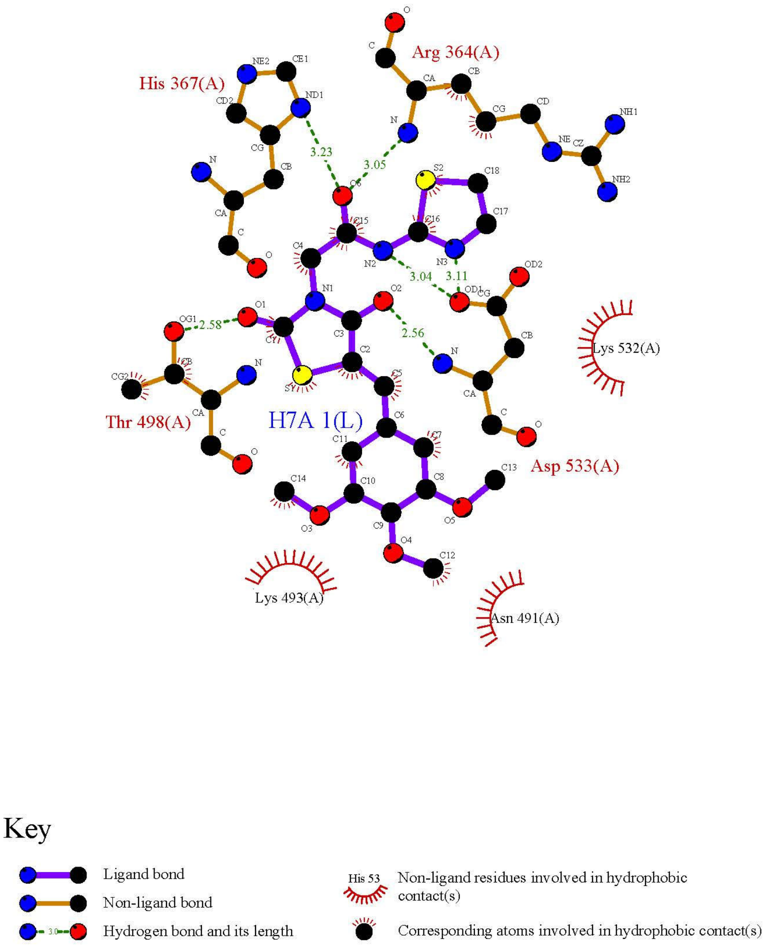

7a-docked

.

Fig. S60.

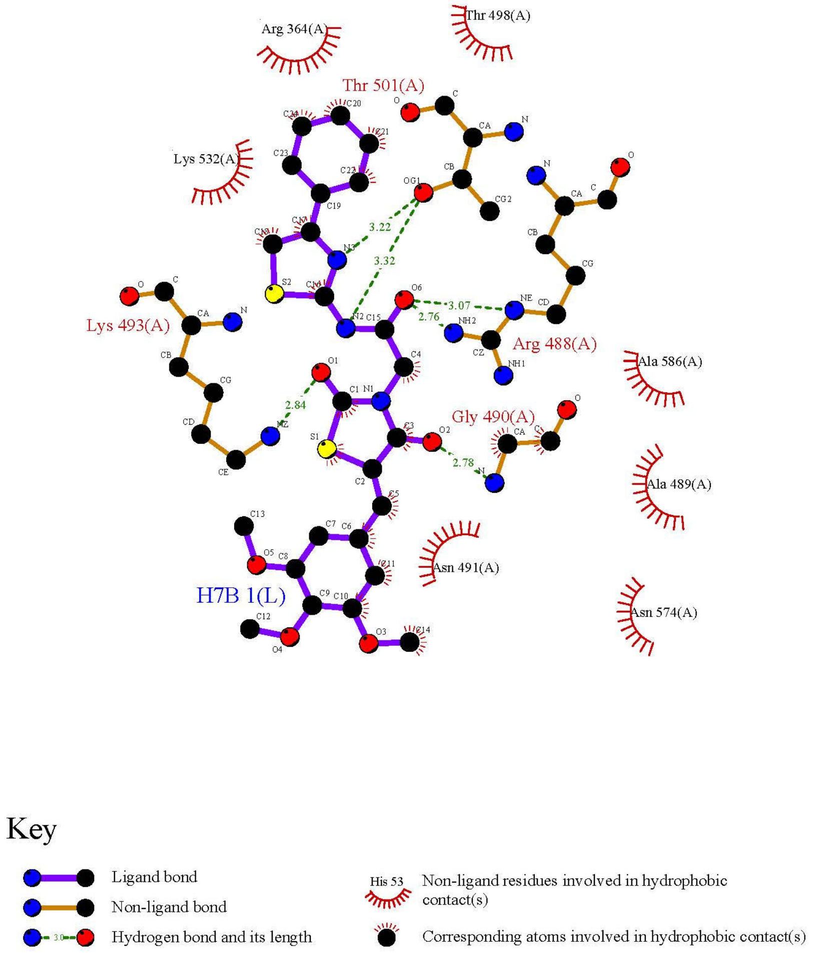

7b-docked

.

Fig. S61.

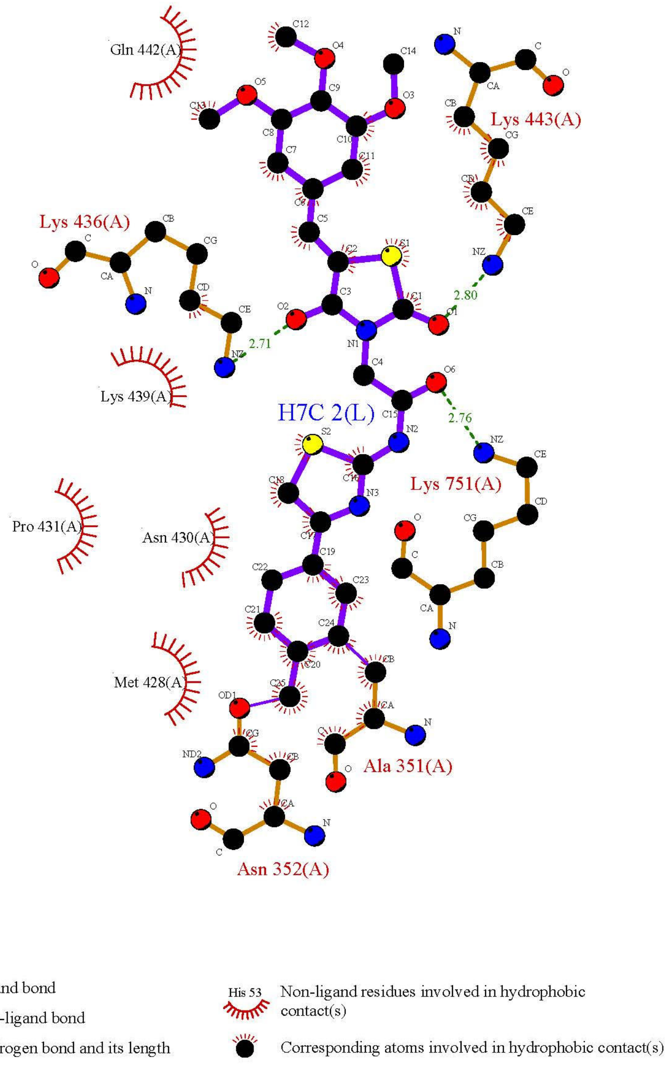

7c-minE

.

Fig. S62.

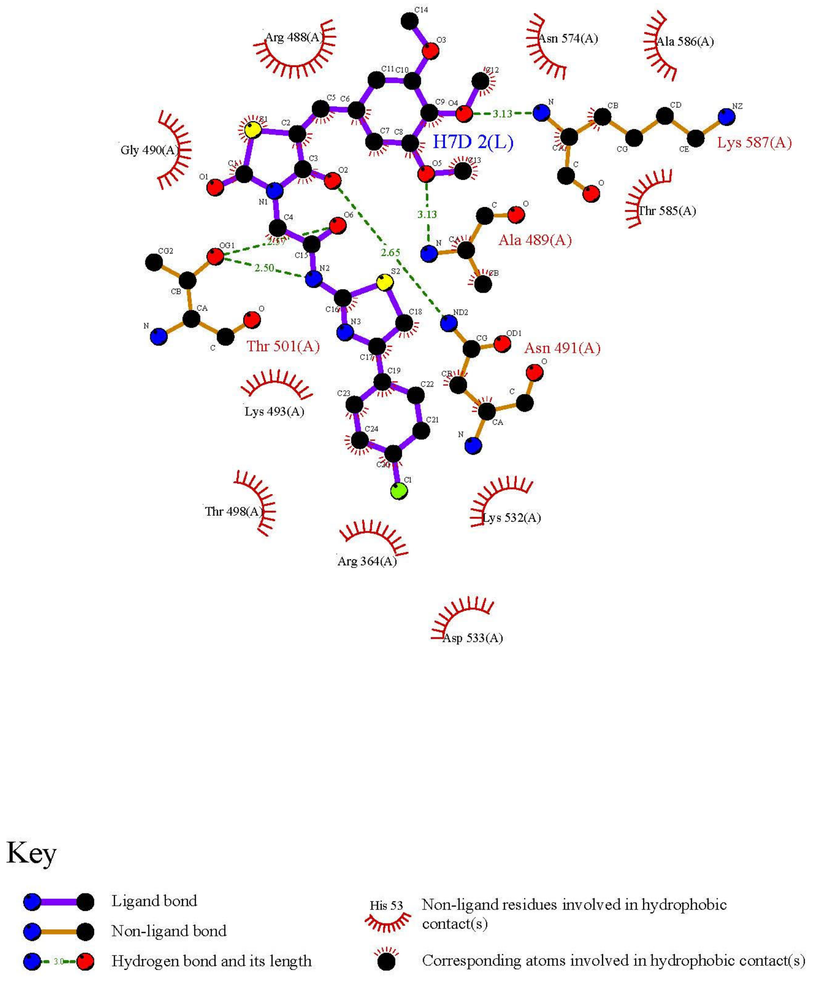

7d-minE

.

Fig. S63.

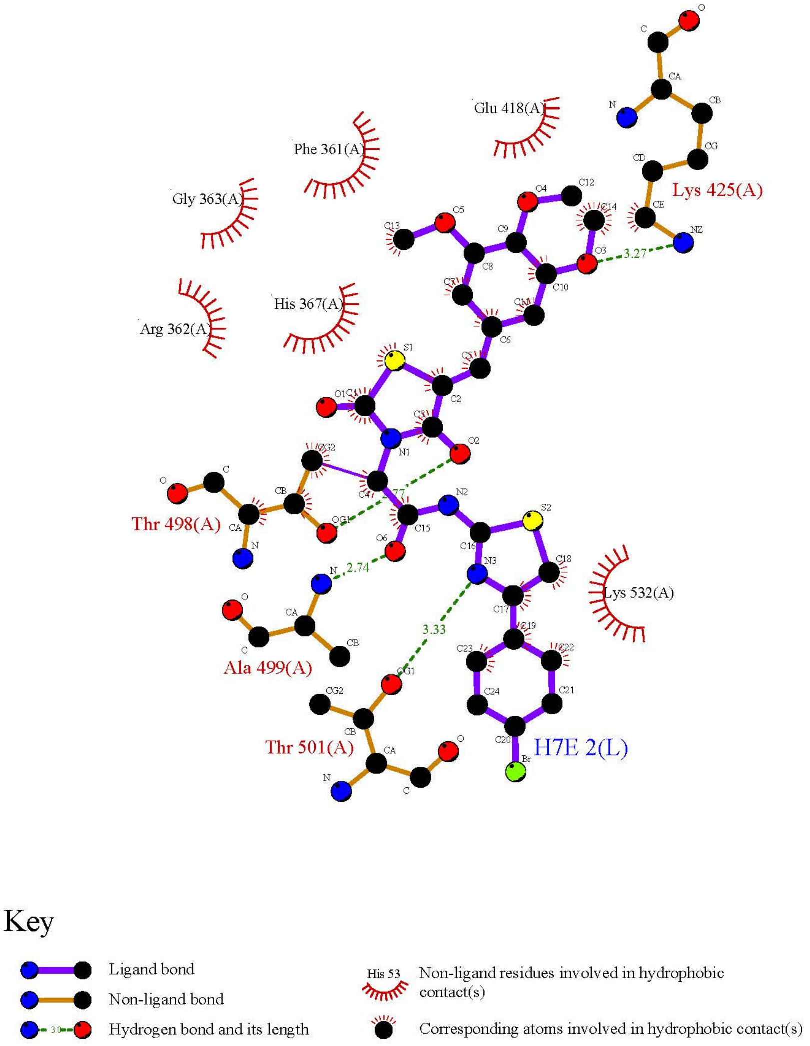

7e-minE

.

Fig. S64.

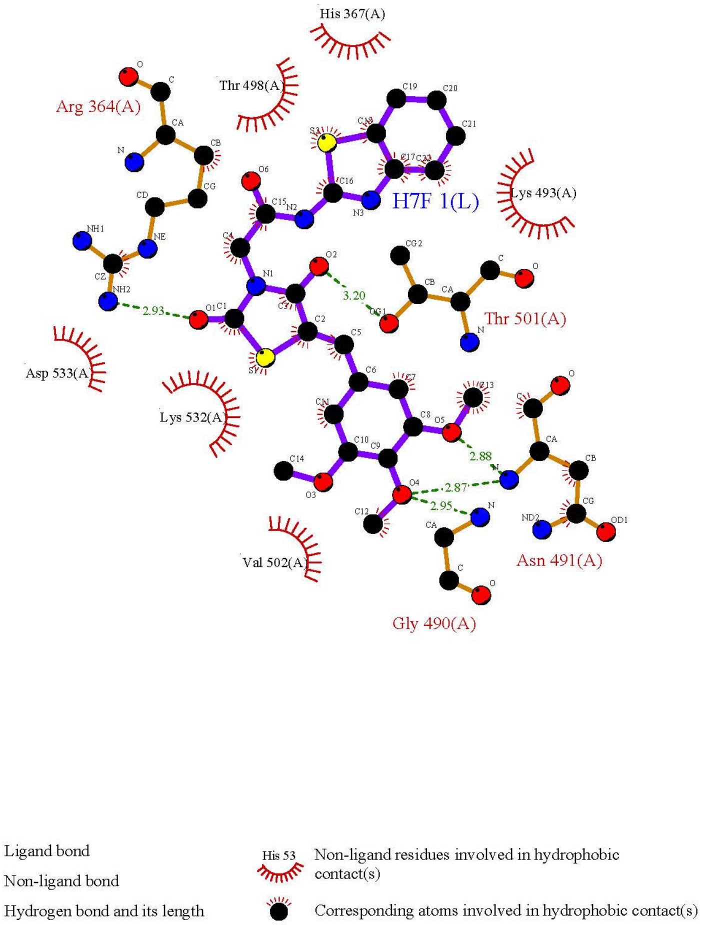

7f-maxpop

.

Fig. S65.

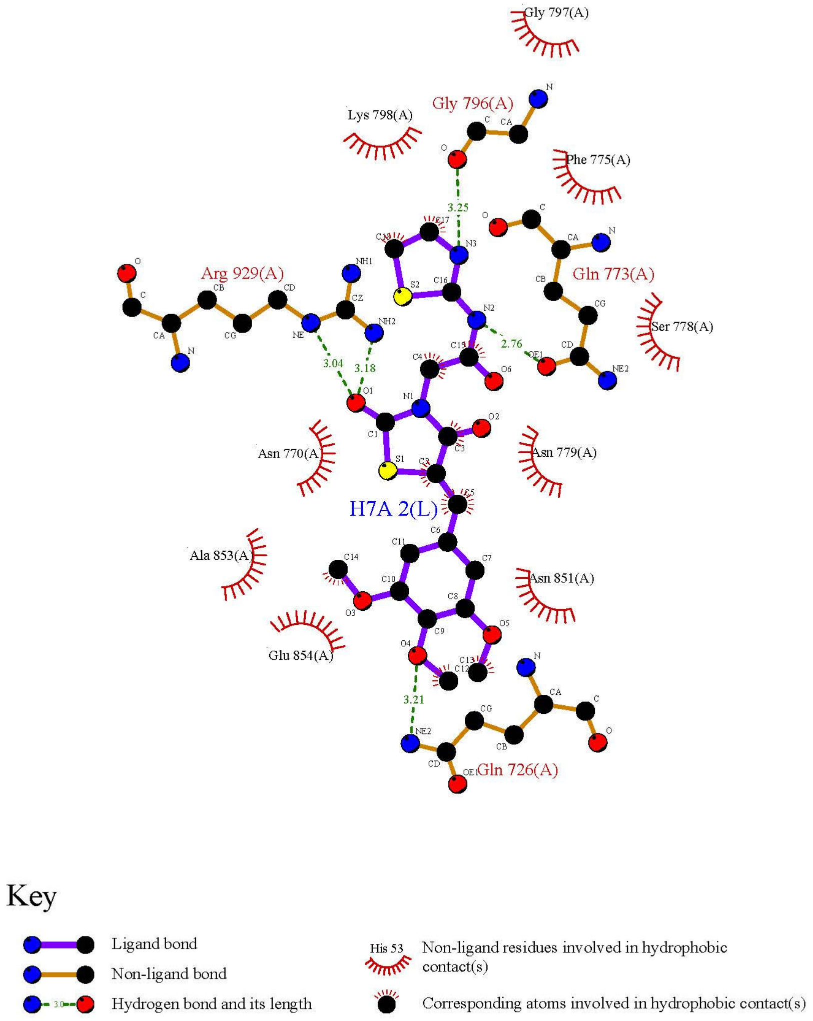

5gwk-7a

.

Fig. S66.

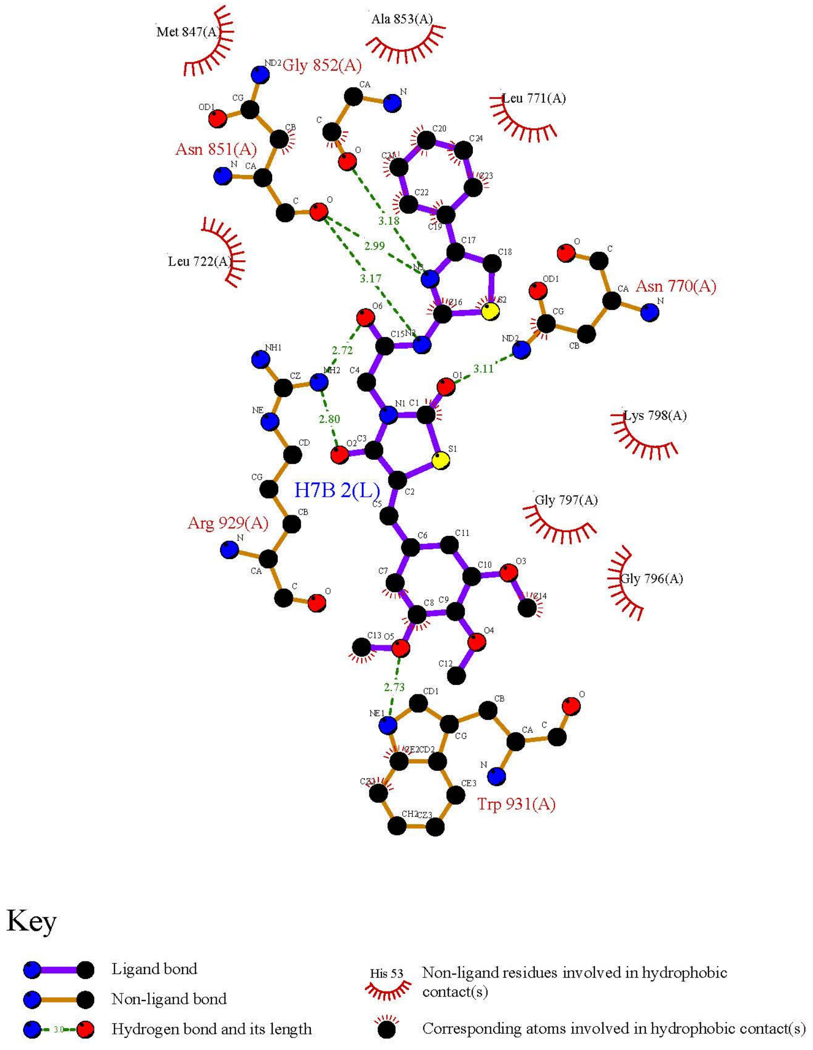

5gwk-7b

.

Fig. S67.

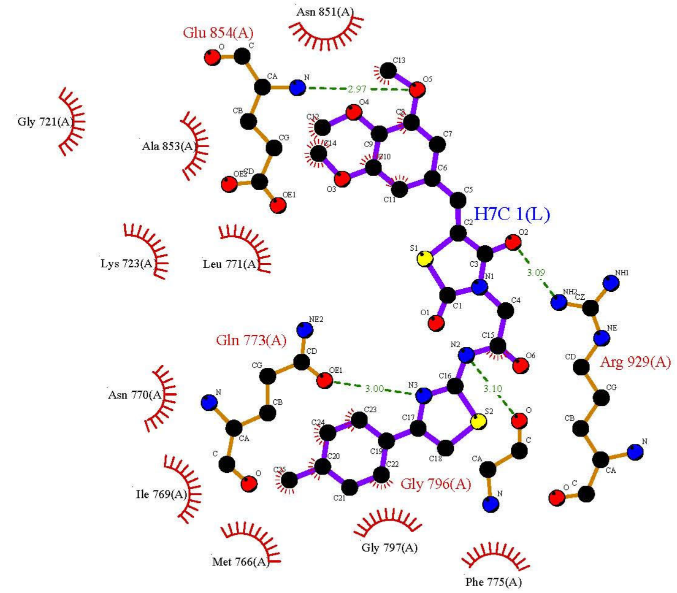

Key

Ligand bond

Non-ligand bond

Hydrogen bond and its length

His 53 Non-ligand residues involved in hydrophobic contact(s)

Corresponding atoms involved in hydrophobic contact(s)

5gwk-7c

.

Fig. S68.

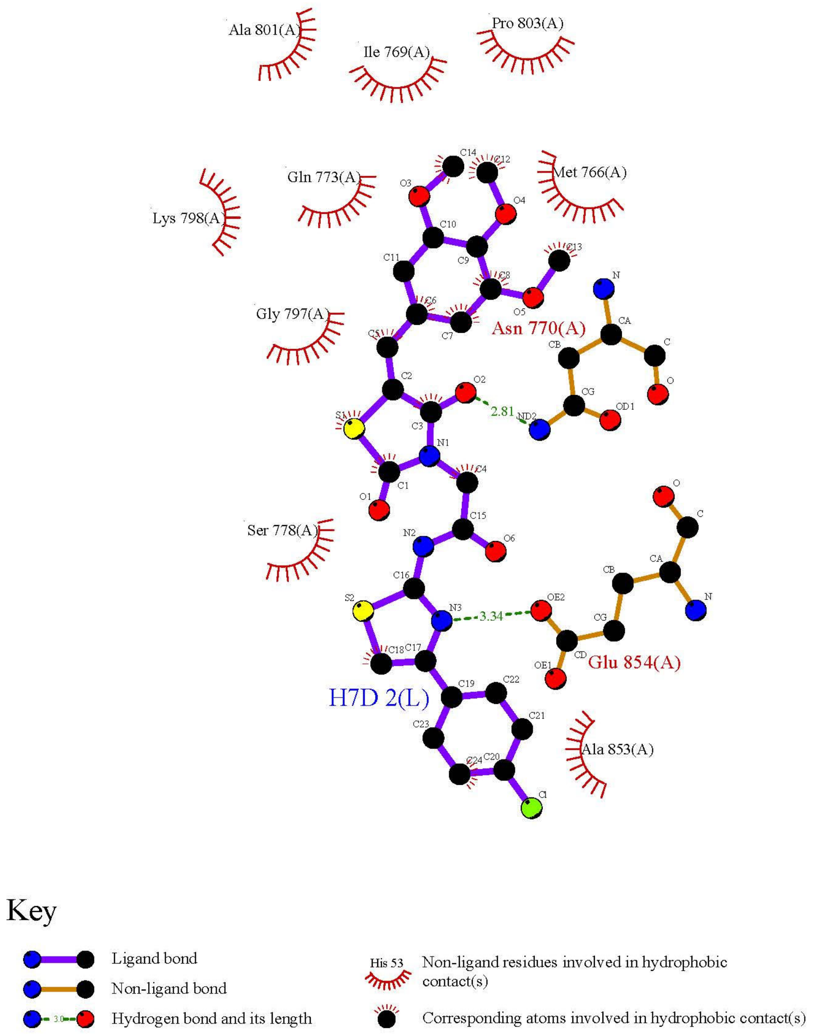

5gwk-7d

.

Fig. S69.

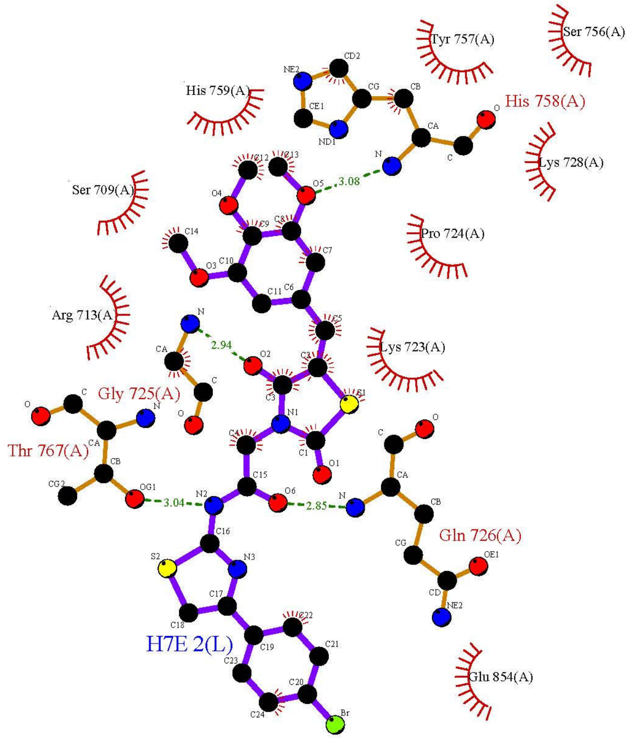

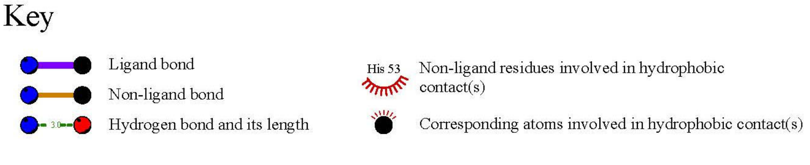

5gwk-7e

.

Fig. S70.

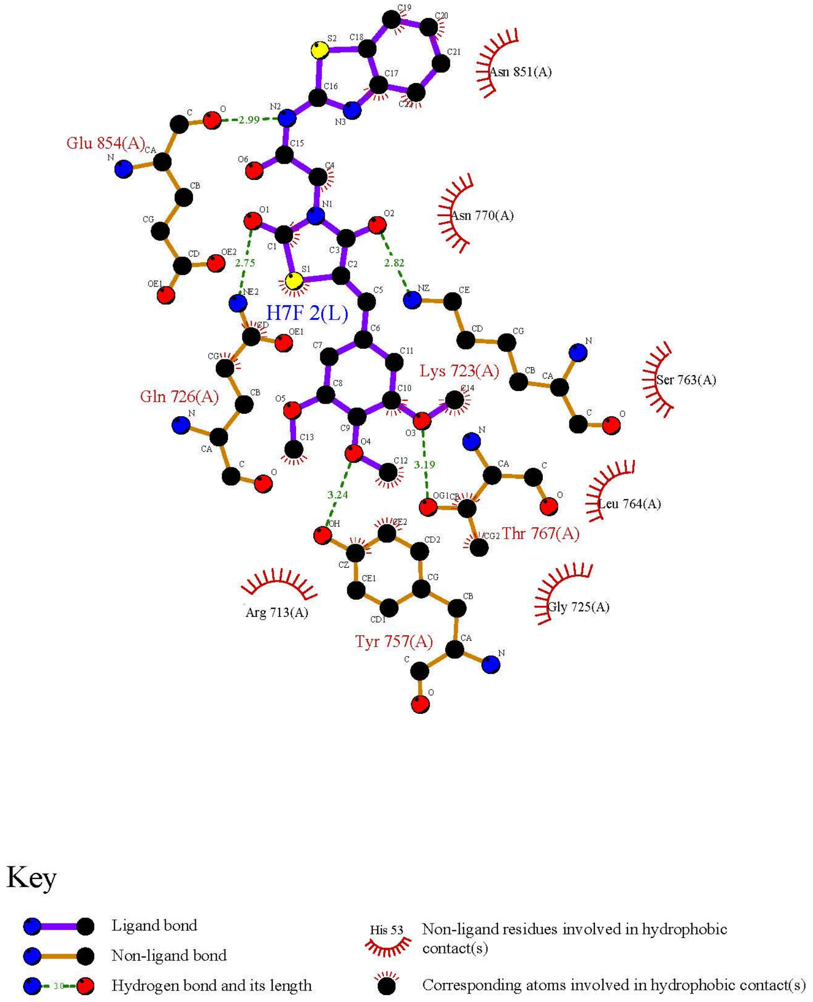

5gwk-7f

.
